# Functional Metagenomics of Spacecraft Assembly Cleanrooms: Presence of Virulence Factors Associated with Human Pathogens

**DOI:** 10.3389/fmicb.2016.01321

**Published:** 2016-09-09

**Authors:** Mina Bashir, Mahjabeen Ahmed, Thomas Weinmaier, Doina Ciobanu, Natalia Ivanova, Thomas R. Pieber, Parag A. Vaishampayan

**Affiliations:** ^1^Biotechnology and Planetary Protection Group, Jet Propulsion Laboratory, California Institute of TechnologyPasadena, CA, USA; ^2^Division of Endocrinology and Diabetology, Medical University of GrazGraz, Austria; ^3^Department of Biological Sciences, California State Polytechnic UniversityPomona, CA, USA; ^4^Division of Computational Systems Biology, Department of Microbiology and Ecosystem Science, University of ViennaVienna, Austria; ^5^Department of Energy, Joint Genome InstituteWalnut Creek, CA, USA

**Keywords:** cleanroom, pathogens, indoor environments, microbiome, spacecraft, virulence factors, *Acinetobacter*, functional metagenomics

## Abstract

Strict planetary protection practices are implemented during spacecraft assembly to prevent inadvertent transfer of earth microorganisms to other planetary bodies. Therefore, spacecraft are assembled in cleanrooms, which undergo strict cleaning and decontamination procedures to reduce total microbial bioburden. We wanted to evaluate if these practices selectively favor survival and growth of hardy microorganisms, such as pathogens. Three geographically distinct cleanrooms were sampled during the assembly of three NASA spacecraft: The Lockheed Martin Aeronautics' Multiple Testing Facility during DAWN, the Kennedy Space Center's Payload Hazardous Servicing Facility (KSC-PHSF) during Phoenix, and the Jet Propulsion Laboratory's Spacecraft Assembly Facility during Mars Science Laboratory. Sample sets were collected from the KSC-PHSF cleanroom at three time points: before arrival of the Phoenix spacecraft, during the assembly and testing of the Phoenix spacecraft, and after removal of the spacecraft from the KSC-PHSF facility. All samples were subjected to metagenomic shotgun sequencing on an Illumina HiSeq 2500 platform. Strict decontamination procedures had a greater impact on microbial communities than sampling location Samples collected during spacecraft assembly were dominated by *Acinetobacter* spp. We found pathogens and potential virulence factors, which determine pathogenicity in all the samples tested during this study. Though the relative abundance of pathogens was lowest during the Phoenix assembly, potential virulence factors were higher during assembly compared to before and after assembly, indicating a survival advantage. Decreased phylogenetic and pathogenic diversity indicates that decontamination and preventative measures were effective against the majority of microorganisms and well implemented, however, pathogen abundance still increased over time. Four potential pathogens, *Acinetobacter baumannii, Acinetobacter lwoffii, Escherichia coli* and *Legionella pneumophila*, and their corresponding virulence factors were present in all cleanroom samples. This is the first functional metagenomics study describing presence of pathogens and their corresponding virulence factors in cleanroom environments. The results of this study should be considered for microbial monitoring of enclosed environments such as schools, homes, hospitals and more isolated habitation such the International Space Station and future manned missions to Mars.

## Introduction

Detection of signs of life on other planets is of particular interest for many of NASA's planetary missions. In order not to mistake earthborn microorganisms for unknown potential extraterrestrial life, planetary missions are subject to internationally accepted standards of planetary protection, established by the Committee of Space Research (COSPAR) (National Research Council, 2006). Jet Propulsion Laboratory's Planetary Protection Group has undertaken huge efforts (NASA Policy Directive (NPD) 8020.7G, 1999) to avoid inadvertent contamination of other planets with earthborn organisms, and to minimize the bioburden on spacecraft. Spores are of particular interest, given their high resistance to multiple sterilization techniques, including radiation (Venkateswaran et al., 2003; La Duc et al., 2007; Vaishampayan et al., 2012).

All spacecraft parts undergo extensive cleaning and sterilization steps, such as exposure to dry heat, vaporized hydrogen peroxide, radiation and alcohol on surfaces. Additional protocols to reduce the influx of particulate matter include daily vacuuming and mopping of floors, HEPA air filtration, regular replacement of tacky mats at all entry points, and strict gowning procedures. These precautions are routinely taken but with high frequency and stringency during the spacecraft assembly. All personnel that enter the cleanroom are required to put on cleanroom garments. This includes a full body suit, hair and beard nets, facemasks, additional head covering, gloves, shoe covers, and cleanroom boots. These are necessary measures since humans are the major source of contamination in these environments (La Duc et al., 2004; Probst et al., 2013). To monitor contamination levels, cleanrooms are regularly sampled for biological activity, particularly when spacecraft parts are being assembled and cleaned (La Duc et al., 2007; Vaishampayan et al., 2010a).

Multiple sterilization methods are chosen, because there is no known method that can eradicate all microbes, which is still compatible with spacecraft components. Only very resistant microorganisms, such as spores, pathogens, and extremophiles, can overcome these strict decontamination procedures (Ghosh et al., 2009; Derecho et al., 2014). Some microorganisms are even able to survive the harsh conditions of interstellar travel. Researchers placed spore-forming bacteria, isolated from cleanroom environment, outside the International Space Station for 18 months along with exposure to simulated Mars-like conditions, including atmospheric pressure and selective UV-radiation and some of them were still able to survive (Vaishampayan et al., 2012).

Our goal was to elucidate whether decontamination measures lead to selection of hardy microorganisms, including pathogens, in the cleanrooms and therefore posing a potential threat to human health. Pathogens might thrive in these environments perhaps due to their selective phenotypic characteristics, metabolic capabilities and reduced competition for scarce nutrients and niches. We were particularly interested in human pathogens, given that humans are the main source of contamination in cleanrooms (La Duc et al., 2004; Probst et al., 2013), and also because they are exposed to these constantly-evolving microbes. Most studies aiming at determining the microbiome of cleanrooms (La Duc et al., 2009; Sandle, 2011; Vaishampayan et al., 2013; Mahnert et al., 2015; Moissl-Eichinger et al., 2015), other indoor environments (Adams et al., 2015) or even the International Space Station (Checinska et al., 2015) have used 16S rRNA amplicon sequencing. 16S rRNA amplicon sequencing is often used to screen for potential pathogens (Case et al., 2007; Stadlbauer et al., 2015; Bashir et al., 2016). However, the lack of discriminability does not allow differentiating between potential and true pathogens. Previous functional metagenomic studies investigated pathogens in other indoor environments (Tringe et al., 2008; Afshinnekoo et al., 2015), but this is the first study, which focuses on the detection of pathogens as well as virulence factors in cleanrooms.

Three geographically distinct cleanrooms were sampled during the assembly of three NASA spacecraft: Phoenix in Cape Canaveral, Florida, DAWN in Fort Worth, Texas, and Mars Science Laboratory (Curiosity) in Pasadena, California. Sample sets from Phoenix mission were collected from the cleanroom at three time points: before arrival of the spacecraft, during the assembly and testing of the Phoenix spacecraft, and after removal of the spacecraft from the facility. All samples were subjected to whole metagenome shotgun sequencing on an Illumina HiSeq 2500 platform. We screened for pathogens and virulence factors, which determine pathogenicity. Clinically relevant pathogens were identified by searching taxonomic classification and potential virulence factors were identified by comparing reads to the Microbial virulence database.

## Materials and methods

### Sample collection and processing

Multiple samples were collected from the floor of the Kennedy Space Center's Payload Hazardous Servicing Facility (KSC-PHSF), where the Phoenix spacecraft was assembled. Sample sets were collected from the KSC-PHSF surfaces at three time points: before arrival of the Phoenix spacecraft (10 samples; PHX-B), during the assembly and testing of the Phoenix spacecraft (8 samples; PHX-D), and after removal of the spacecraft from the KSC-PHSF facility (10 samples; PHX-A). 10 samples from the Lockheed Martin Aeronautics' Multiple Testing Facility (LMA-MTF) floor were collected during the DAWN spacecraft assembly. Samples were collected from the Ground Support Equipment (GSE) at Jet Propulsion Laboratory's spacecraft assembly facility (JPL-SAF) during the Mars Science Laboratory (2 samples; MSL) spacecraft assembly. These three cleanroom facilities were certified at ISO 8 (3,520,000 particles >0.5 μm m^−3^) level and maintained according to the standard cleaning practices. Each sample was collected from 1 m^2^ of the cleanroom floor or GSE by a wet surface sampling technique using Biological Sampling Kits (BiSKits, QuickSilver Analytics, Abingdon, Md.) and polyester wipes, respectively. Samples from each sampling event were concentrated using Amicon Ultra-15 centrifugal filter tube (Millipore, Jaffrey, NH, Ultracel-50 membrane) as described earlier (La Duc et al., 2009). DNA was extracted from each concentrated sample using bead beating and an automated DNA extraction instrument (Autolyser A-2 DNA, Axcyte Genomics, Menlo Park, CA) and pooled equimolar, as described earlier (Vaishampayan et al., 2010b). DNA samples were archived at −80°C until further use. Negative controls such as field control (sampling devise control), reagent control (during DNA extraction) at each step were collected. None of the negative controls had a sufficient DNA concentration for library preparation and were thus not included in further downstream analysis.

### Metagenomic sequencing

Sample processing was performed in a sodium hypochlorite (bleach) treated laminar flow hood in an ultra-clean environment. Operators were using single-use lab-coats, bleached gloves, hairnets, and booties. Due to low DNA concentrations, samples were subject to multiple displacement amplification (MDA) (Dean et al., 2002). Each sample was divided into 1 ml aliquots, which were amplified via MDA using Repli-g single-cell whole genome amplification kit (Qiagen part #150345). All plastic ware and water were ultraviolet (UV) treated in a Stratalinker 2400 UV Crosslinker (Stratagene, La Jolla, CA) with 254-nm UV for 30–90 min on ice (Woyke et al., 2011). This represents a UV dose range of 5.7–17.1 J/cm2, calculated by measuring the distance from inside the tubes to the light bulb (4 cm). Buffer and enzyme come pre-cleaned and don't require UV-radiation. MDA reaction was prepared following manufacturer protocol for single cells, scaling reaction volume down to 15 μl final volume and addition of Syto13 dye for real-time monitoring. MDA reaction was stopped when sample amplification reached saturation.

Amplified fractions of each sample were combined, and this pooled DNA product (100 μl) was sheared using a Covaris E210 instrument (Covaris, Woburn, MA) set to: 10% duty cycle, intensity 5, and 200 cycles per burst for 1 min. The concentration and fragment size of each sheared product was determined using Agilent 2100 Bioanalyzer (Agilent Technologies, Santa Clara, CA) in accordance with the manufacturer's recommended conditions. The sheared DNA was end-repaired, A-tailed, and ligated to Illumina adaptors according to standard Illumina PE protocols (Illumina, San Diego, CA). The concentration of the resulting Illumina-indexed libraries was again determined using Agilent 2100 Bioanalyzer (Agilent Technologies, Santa Clara, CA). Libraries were pooled and normalized to a final concentration of 400 mM each, and the primary bands corresponding to the sizes were gel-purified and dissolved in 30 μl TE. One flow-cell was generated from a pooled library, which was subsequently subjected to sequencing in an Illumina HiSeq2500 instrument (2 × 250 bp), in accordance with manufacturer-provided protocols. The raw sequence data are available within IMG/M (https://img.jgi.doe.gov/cgi-bin/mer/main.cgi) and NCBI's short read archive under the accession number SRP077843.

### Sequence data analysis

We started with a total of 15,001,132 paired reads for PHX-B, 14,654,014 for PHX-D, and 22,355,430 for PHX-A before quality filtering and pairing. For MSL and DAWN we had 57,892,216 and 2,899,364 reads, respectively (Table 1).

**Table 1:** **Data statistics: number of reads per sample starting with raw reads coming from the sequencer until final taxonomic and functional classification**.

**Sequences**	**PHX-B**	**PHX-D**	**PHX-A**	**DAWN**	**MSL**
Paired raw reads	15,001,132	14,654,014	22,355,430	2,899,364	57,892,216
Passed quality filter	10,760,642	11,889,258	16,338,684	166,392	34,615,498
KEGG assignment	13,360	24,916	298,350	557	664,699
With Taxonomic classification	174,622	1,328,890	2,903,271	17,306	7,652,616
Metabolic diversity	145.8	188.5	42.9	119.8	5.5
Observed genera	396	36	104	82	25

FastQC v0.10.1 (Andrews, 2010) was used to determine the base quality throughout the 250 bp HiSeq-generated paired-end reads. PEAR v0.9 (Zhang et al., 2014; default parameters) was used to merge paired reads. Unmerged forward and reverse reads were retained. Merged and unmerged reads were processed using prinseq-lite v0.20.3 (Schmieder and Edwards, 2011) with the following parameters: “-min_len 100 -trim_qual_right 20 -trim_qual_left 20 -trim_left 8.” Adapter sequences and overrepresented sequences were identified with FastQC and removed using Cutadapt v1.1 (Martin, 2011). PhiX174 and a JGI-standard collection of potential contaminant genomes (Supplementary Table 1) were removed by mapping trimmed high-quality reads using BBMap short read aligner v31.18 (Bushnell, 2014) to the respective genomes. Any reads matching any of these contaminant genomes were removed from the dataset.

To generate the human DNA sequence free dataset, all remaining high-quality reads were mapped with BBMap short read aligner against the human genome GRCh38 (including mitochondrial DNA). All positive matches were removed from the dataset.

Both, datasets including and excluding human DNA sequences were compared to NCBI non-redundant database using DIAMOND BLASTX v0.7.1 (Buchfink et al., 2014) with default parameters. Results were imported to MEGAN v5.10.5 (Huson et al., 2007; minimal bit score of 80%; “minscore 80”) for taxonomic binning, functional assignments to KEGG functions, and generation of rarefaction curves (phylogenetic diversity on genus level). After removal of unassigned and unclassified reads, taxonomy and KEGG pathways (Kanehisa and Goto, 2000; Kanehisa et al., 2014) were visualized using Krona Tools v2.4 (Ondov et al., 2011). Taxonomic and metabolic diversity calculations were done in QIIME 1.9.1 (Caporaso et al., 2011) with all samples subsampled to the smallest sample size observed.

Potential virulence factors were identified by comparing contaminant- and human-DNA-sequence-free reads to the Microbial Virulence Database MvirDB (Zhou et al., 2007) using DIAMOND BLASTX (Buchfink et al., 2014) with a 80% sequence similarity cut-off and maximum of target sequences of one. Sequences which passed these criteria were extracted and compared to NCBI non-redundant database using DIAMOND BLASTX (Buchfink et al., 2014) with a maximum of target sequences of one for virulence factor validation (Data Sheet 1 in Supplementary Material). Classified sequences were searched for clinically relevant pathogens (http://www.bode-science-center.com/center/relevant-pathogens-from-a-z.html accessed on Dec 1 2015; Supplementary Table 2).

## Results

### Phylogenetic diversity of cleanroom samples

**Figure 1:**
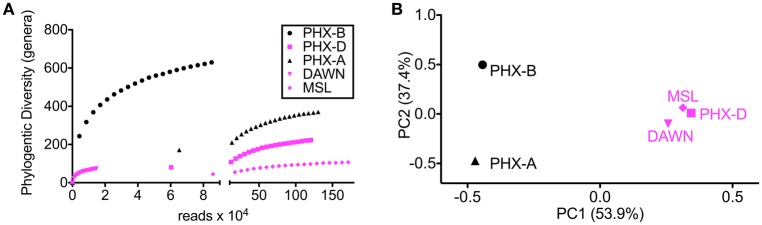
**Diversity is lower during assembly. (A)** Rarefaction curves of samples taken during assembly (PHX-D, DAWN, MSL) show less genera at the same sample size compared to samples taken before (PHX-B) and after (PHX-A) assembly. **(B)** Principal coordinates analysis on genus taxonomic level based on a Bray-Curtis dissimilarity matrix. Samples taken during spacecraft assembly show a similar community profile although the sampling locations were hundreds of miles apart.

**Figure 2:**
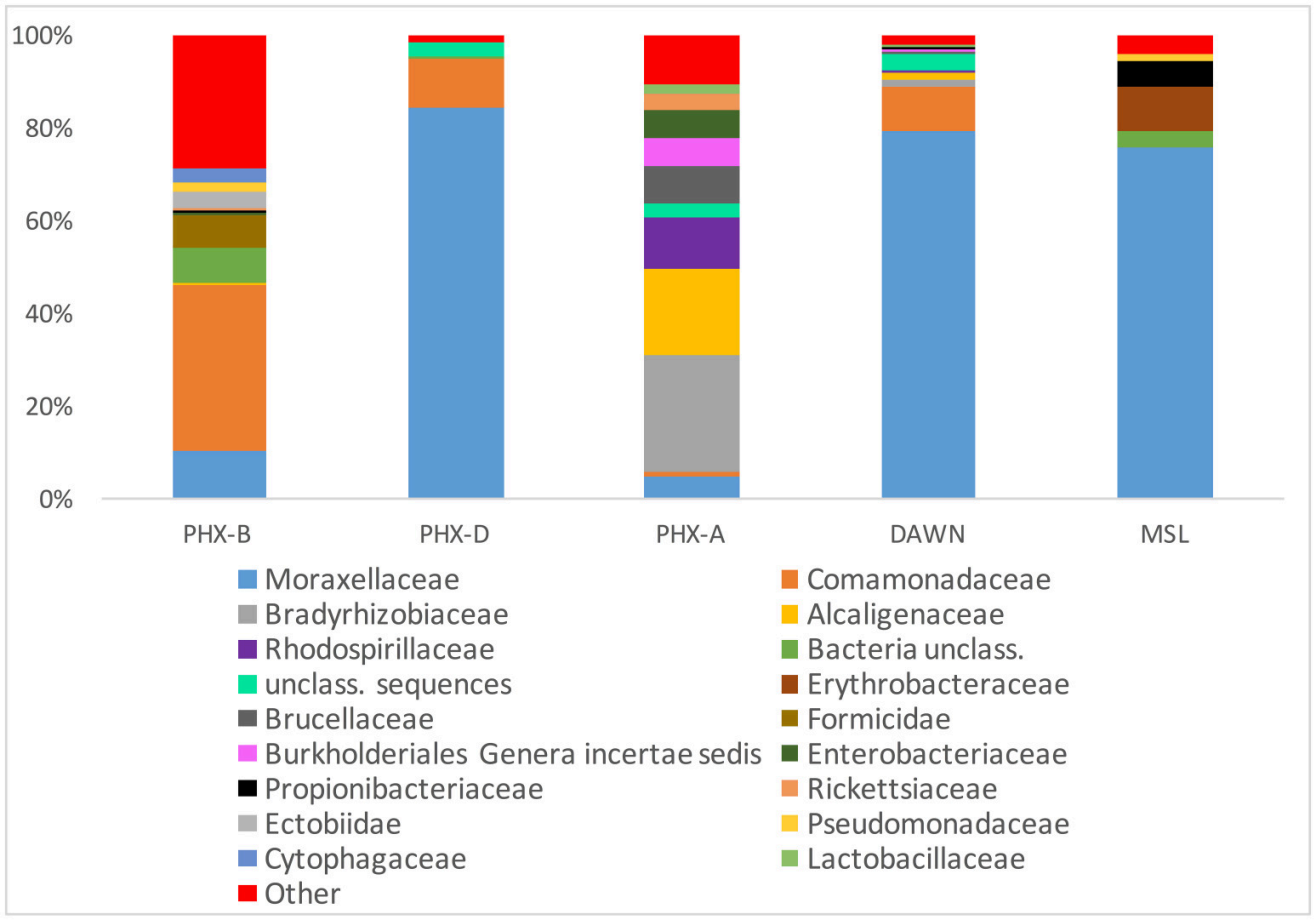
**Moraxellaceae dominate cleanroom during spacecraft assembly**. Relative abundance of taxa at family level. Human, viral and archaeal impact was minimal. All taxa with a collective abundance of equal or less than 2% from all samples were combined in “Other”. See Presentation S1 in Supplementary Material for more details and lower taxonomic levels.

Alpha rarefaction curves indicate, besides sufficient sampling efforts, that diversity is drastically lower during the actual spacecraft assemblies (PHX-D, DAWN, and MSL) compared to before or after. This confirms that the very strict gowning, cleaning and sterilization procedures were well executed, and highly effective as previously described (Ghosh et al., 2010). MSL had the highest sampling depth but lowest bioburden (Figure 1A) as GSE undergo stringent cleaning procedures and are exposed to less handling and human contact compared to the cleanroom floors. Interestingly, microbial community profiles during active spacecraft assembly (PHX-D, DAWN, MSL) were more similar to each other than to samples from one location (Figure 1B). *Moraxellaceae* was the dominating family, making 83, 73, and 62% of all classified sequences for PHX-D, DAWN, and MSL, respectively. The majority of all *Moraxellaceae*, 94% to 100%, were *Acinetobacter* spp. (Figure 2 and Presentation S2 in Supplementary Material), making it the most dominating taxa during spacecraft assembly.

In general, bacteria were the most dominant kingdom present in all tested cleanrooms with 63 to >99% of all classified sequences. Archaea and viruses on the other hand accounted for less than 0.1% relative abundance combined (Figure 2 and Presentation S2 in Supplementary Material). Surprisingly, the amount of potentially human DNA was minimal. Only 0.04–2% of all sequences were classified as primates: PHX-B 2%, PHX-D 0.08%, PHX-A 0.2, DAWN 0.05, and MSL 0.2% (Presentation S2 in Supplementary Material).

In PHX-B eukaryotes made 36% of all classified sequences. Most of these sequences (22% of total; 60% of eukaryotes) belong to the class of arthropods, such as insects and arachnids. In all other samples less than 0.1% of all classified sequences were arthropods. Probably arthropod sequences originated from free DNA associated with dust particles, given that no living spiders or insects are present in any cleanrooms. In MSL all eukaryotic sequences were assigned to craniate. Fungi were also not prominent in our cleanrooms. The fungal abundance ranged from 0.0008% (MSL) to 1% (PHX-B) (Presentation S2 in Supplementary Material).

### Metabolic diversity during spacecraft assembly

**Figure 3:**
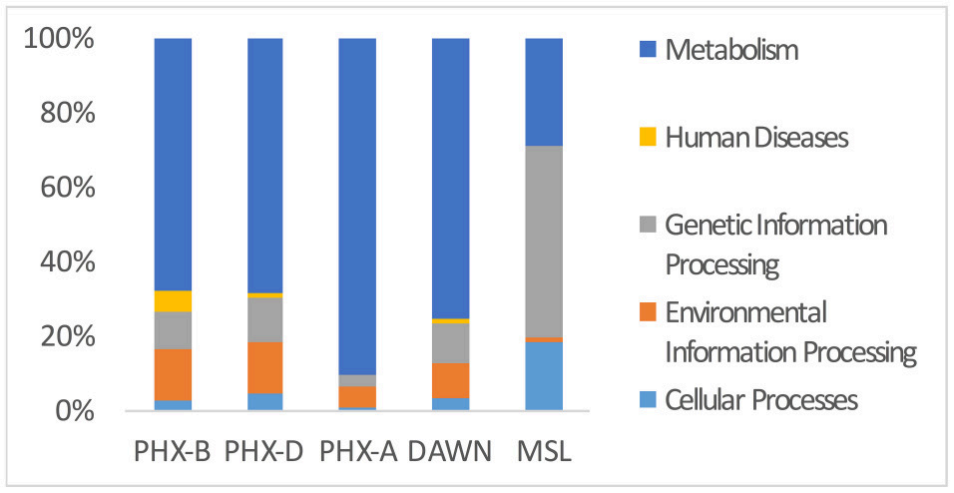
**Metabolic genes cover majority of functional classifications**. KEGG Pathway analysis of PHX-B, PHX-D, PHX-A, DAWN and MSL. During assembly of PHX-D and DAWN, we see that a bigger fraction of all classified sequences have been assigned to metabolism. See Presentation S2 in Supplementary Material for more details.

Functional assignment resulted in 13,360 KEGG orthologous (KO) for PHX-B, 24,916 KOs for PHX-D, 298,350 KOs for PHX-A, 557 for DAWN and 664,699 for MSL (Table 1 and Data Sheet 1 in Supplementary Material). Figure 3 indicates that the majority of the functional classification was assigned to metabolism (PHX-B 67%, PHX-D 67%, PHX-A 90%, DAWN 75%, MSL 29%; Figure 3 and Data Sheet 1 in Supplementary Material). Although the percentage of sequences assigned to metabolism did not differ much across samples (Figure 3 and Data Sheet 1 in Supplementary Material), we saw a higher metabolic diversity during assembly compared to before or after spacecraft assembly samples (Table 1). We also found that the metabolism of pantothenate and coenzyme A is higher during assembly (PHX-D 4%, DAWN 2%) compared to PHX-B or PHX-A, 0.2% respectively (Data Sheet 1 in Supplementary Material). Nevertheless, no function associated with pantothenate and coenzyme A was found in MSL during assembly. Fifty-two percent of all functional classification from MSL was assigned to Holliday junction DNA helicase RuvB (Genetic Information Processing; Replication and Repair). In PHX-B, 9% were assigned to Genetic Information Processing, 12%, in PHX-D 3%, in PHX-A 11% in DAWN, and 52% in MSL (Figure 3).

### Potential pathogens in cleanroom samples

After taxonomic classification we selectively screened the classified binned sequences of all our samples for clinically relevant pathogens (Supplementary Table 2). In total we found 48 different human pathogens in all cleanrooms, responsible for various diseases, from gastrointestinal, to the nervous system. Twenty nine different pathogens were detected in PHX-B, 18 in PHX-D, 33 in PHX-A, 10 in DAWN and 11 in MSL (Table 2). Strikingly, four pathogens, namely *Acinetobacter baumannii, Acinetobacter lwoffii, Escherichia coli* and *Legionella pneumophila*, were detected in all cleanrooms, even though they are geographically separate. Besides these four pathogens, present in all samples, we found pathogens that were exclusive to KSC-PHSF, during all three time points, namely, *Bacillus cereus, Burkholderia pseudomallei, Enterobacter cloacae, Enterococcus faecalis, Listeria monocytogenes, Pseudomonas aeruginosa, Staphylococcus aureus* and *Staphylococcus epidermidis*. In case of PHX-D, 83% of all classified reads were identified as *Acinetobacter* spp. (Presentation S2 in Supplementary Material), which is listed as a clinically relevant pathogen (Supplementary Table 2). Additionally, we found a decreased pathogen diversity during the actual spacecraft assembly in KSC-PHSF, while pathogen abundance almost triples over time (PHX-B 1.52, PHX-D 2.34, and PHX-A 4.26%; Table 2).

**Table 2:** **Pathogen diversity is lowest during assembly: pathogens found in the different cleanroom samples**.

	**Pathogen**	**PHX-B (%)**	**PHX-D (%)**	**PHX-A (%)**	**DAWN (%)**	**MSL (%)**
Urinary tract infection (catheter-associated or otherwise)	Acinetobacter baumannii^*^	0.2	2	0.5	2	0.8
	Acinetobacter lwoffii^*^	0.1	0.3	0.001	0.2	0.0006
	*Bacillus cereus*	0.1	0.0002	0.005	–	–
	*Brevundimonas diminuta*	0.007	0.02	–	0.02	–
	*Burkholderia cepacia*	0.02	–	–	–	–
	*Enterobacter aerogenes*	–	–	0.004	–	–
	*Enterobacter cloacae*	0.01	0.0002	0.0002	–	–
	*Enterococcus faecalis*	0.0006	0.00008	0.05	0.01	–
	*Enterococcus faecium*	0.0006	–	–	–	–
	Escherichia coli^*^	0.06	0.004	3	0.2	0.004
	*Klebsiella pneumoniae*	0.003	–	0.0004	–	0.00004
	*Providencia stuartii*	–	–	0.00003	–	–
	*Pseudomonas aeruginosa*	0.2	0.0002	0.0004	–	0.2
	*Serratia marcescens*	–	0.002	–	–	–
	*Staphylococcus haemolyticus*	0	–	0.001	–	–
	*Staphylococcus saprophyticus*	–	–	0.003	–	–
	*Stenotrophomonas maltophilia*	0.3	0.003	–	–	0.0004
	Abundance (%)	1.0012	2.3297	3.5650	2.4300	1.0050
	Number of pathogens	13	10	12	5	6
Pneumonia, respiratory disease or infections	Acinetobacter baumannii^*^	0.2	2	0.5	2	0.8
	*Brevundimonas diminuta*	0.007	0.02	–	0.02	–
	*Burkholderia cepacia*	0.02	–	–	–	–
	*Chlamydia psittaci*	–	–	0.00003	–	–
	*Coxiella burnetii*	0.001	–	–	–	–
	*Enterobacter aerogenes*	–	–	0.004	–	–
	*Klebsiella pneumoniae*	0.003	–	0.0004	–	0.00004
	Legionella pneumophila^*^	0.0006	0.0005	0.07	0.006	0.0005
	*Serratia marcescens*	–	0.002	–	–	–
	*Streptococcus pneumoniae*	–	–	0.0001	–	–
	*Streptococcus pyogenes*	–	–	0.0003	–	–
	*Histoplasma capsulatum*	0.002	–	–	–	–
	Abundance (%)	0.2336	2.0225	0.5748	2.0260	0.8005
	Number of pathogens	7	4	7	3	3
Meninges, central and peripheral nervous system	*Bacillus cereus*	0.1	0.0002	0.005	–	–
	*Burkholderia pseudomallei*	0.006	0.00008	0.02	–	–
	*Clostridium botulinum*	–	–	0.0004	–	–
	*Haemophilus influenzae*	–	–	0.004	–	–
	*Leptospira interrogans*	–	–	0.0001	–	–
	*Neisseria meningitidis*	–	–	0.3	0.02	0.00008
	*Polyomavirus*	0.009	–	–	–	–
	*Streptococcus pyogenes*	–	–	0.0003	–	–
	Abundance (%)	0.1150	0.0003	0.3298	0.0200	0.0001
	Number of pathogens	3	2	7	1	1
Cardiovascular (sepsis, endocarditis)	*Candida parapsilosis*	–	–	0.00003	–	–
	*Enterococcus hirae*	0.0006	–	–	–	–
	Escherichia coli^*^	0.06	0.004	3	0.2	0.004
	*Haemophilus influenzae*	–	–	0.004	–	–
	*Neisseria meningitidis*	–	–	0.3	0.02	0.00008
	*Pseudomonas aeruginosa*	0.2	0.0002	0.0004	–	0.2
	*Serratia marcescens*	–	0.002	–	–	–
	*Staphylococcus capitis*	0.0006	–	0.001	–	–
	*Staphylococcus epidermidis*	0.002	0.0005	0.04	0.006	–
	*Staphylococcus lugdunensis*	–	–	0.00003	–	–
	*Streptococcus pneumoniae*	–	–	0.0001	–	–
	*Streptococcus pyogenes*	–	–	0.0003	–	–
	*Trichinella spiralis*	0.02	–	–	–	–
	Abundance (%)	0.2826	0.0067	3.3458	0.2260	0.2041
	Number of pathogens	6	4	10	3	3
Gastrointestinal (gastroenteritis, stomach ulcers, diarrhea)	*Campylobacter coli/jejuni*	0.2	–	–	–	–
	*Corynebacterium ulcerans*	0.0006	–	–	–	–
	*Helicobacter pylori*	–	0.00008	–	–	–
	*Listeria monocytogenes*	0.002	0.00008	0.02	–	–
	*Providencia stuartii*	–	–	0.00003	–	–
	*Vibrio cholerae*	–	0.002	0.2	0.02	0.00008
	Abundance (%)	0.2026	0.00216	0.22003	0.02	0.00008
	Number of pathogens	2	3	3	1	1
Skin, wound, or surgical opening	*Burkholderia pseudomallei*	0.006	0.00008	0.02	–	–
	*Candida parapsilosis*	–	–	0.00003	–	–
	*Enterobacter cloacae*	0.01	0.0002	0.0002	–	–
	*Pediculus humanus corporis*	0.2	–	–	–	0.0007
	*Staphylococcus aureus*	0.07	0.003	0.03	–	0.0002
	*Staphylococcus haemolyticus*	0.002	0.0005	0.04	0.006	–
	*Staphylococcus lugdunensis*	–	–	0.00003	–	–
	*Streptococcus pyogenes*	–	–	0.0003	–	–
	Abundance (%)	0.288	0.00378	0.09056	0.006	0.0009
	Number of pathogens	5	3	7	0	2
Typhus	*Orientia tsutsugamushi*	0.0006	–	–	–	–
	*Rickettsia prowazekii*	0.0006	–	0.0005	–	–
Glanders, Malleus	*Burkholderia mallei*	–	–	0.01	0.006	–
Gonorrhea	*Neisseria gonorrhoeae*	–	–	0.0005	–	–
Pathogens associated with more than two diseases	*Alcaligenes faecalis*	–	0.0005	0.0002	–	–
	Peritonis, meningitis, otitis media, appendicitis, blood stream infection					
	*Bacillus anthracis*	–	–	0.0001	–	–
	Anthrax (pulmonary, cutaneous, and gastrointestinal)					
	*Trichinella spiralis*	0.02	–	–	–	–
	Aedema, urticaria, meningitis, encephalitis, myocarditis, and pneumonia					
Number of pathogens		29	18	33	10	11
Total pathogen abundance (%)		1.5168	2.3364	4.2623	2.4880	1.0066

−…Not present.

* …Pathogens found in all cleanroom samples.

### Pathogens and corresponding virulence factors in cleanrooms

Virulence factors are features, which distinguish pathogens from commensals or symbionts (Das et al., 2011). We found that the fraction of sequences identified as potential virulence factors increased over time in case of Phoenix (Table 3), although overall diversity was lower during assembly (Figure 1A). DAWN had approximately half the virulence factor fraction compared to Phoenix, but MSL, which was sampled from GSEs, had approximately 20 times less potential virulence factors compared to Phoenix.

**Table 3:** **Accumulation of virulence factors over time: total number of virulence factors and hits normalized to hits per million reads found in cleanrooms**.

**Sample**	**Total MvirDB hits**	**MvirDB hits/Mio reads**
PHX-B	252	23
PHX-D	5662	476
PHX-A	12703	777
DAWN	39	234
MSL	615	18

To evaluate if we could find pathogens and their corresponding virulence factors, we identified potential virulence factors that are specifically associated with the pathogens found in our samples. We found 14 different potential virulence factors, which correspond to the classified pathogens in PHX-B, 48 for PHX-D and 41 for PHX-A. Nine different virulence factors were found to correspond with the classified pathogens in DAWN, and 6 were found for the MSL mission.

We were particularly interested in detecting potential virulence factors of the four pathogens, *A. baumannii, A. lwoffii, E. coli*, and *L. pneumophila*, which were found in all geographically separated cleanrooms. We found a *Acinetobacter* sp. specific aminoglycoside 6′-N-acetyltransferase lv and tetA, which are kanamycin B and tetracycline resistance genes, respectively, in PHX-D. Moreover, we found adeABC, which is an *A. baumannii* specific multidrug efflux pump and beta-lactamase TEM-1 (Supplementary Table 3).

We found that the abundance of potential virulence factors with associated pathogens increased over time (Table 4). Although, virulence factors diversity did not change over time, we observed a trend toward increased pathogens with associated virulence factors (Table 4, pathogenic diversity). Again, MSL had the smallest pathogenic diversity.

**Table 4:** **Virulence factors with their corresponding pathogens**.

	**PHX-B**	**PHX-D**	**PHX-A**	**DAWN**	**MSL**
Sum	24	2867	5458	16	501
Sum norm.	2	241	334	96	15
Virulence diversity	14	48	41	9	6
Virulence diversity norm.	1	4	2	54	0.2
Pathogenic diversity^*^	3	6	11	3	2

norm: normalized to counts per million reads.

* Number of pathogens with ≥1 corresponding virulence factors.

## Discussion

In this study, we demonstrated for the first time the presence of pathogens and their corresponding virulence factors in spacecraft assembly cleanrooms. Our approach allowed us not only to prove the presence of pathogens in the spacecraft assembly cleanrooms, but also their associated potential virulence factors. Most studies investigating the cleanroom microbiome have only used 16S rRNA amplicon sequencing (Vaishampayan et al., 2013; Mahnert et al., 2015). For example, the archived samples from KSC-PHSF during the Phoenix mission used in this study have previously been described using a cultivation based (Ghosh et al., 2010) and cultivation independent technique (Vaishampayan et al., 2010a). On one hand, cultivation based techniques offer a very limited insight into the wide spectrum of microbial diversity, given that most microorganisms are not cultivable, while 16S rRNA amplicon sequencing on the other hand shows a more broad picture, but does not allow a reliable phylogenetic classification below genus level and does not provide any information regarding virulence factors and potential pathogenicity.

We observed that cleanroom samples are dominated by bacteria as reported previously (Weinmaier et al., 2015). Contrary to previous studies, which found substantially more human, archaeal and viral sequences in cleanrooms (Moissl-Eichinger, 2011; Weinmaier et al., 2015), we found significantly less of each taxon in all cleanrooms tested during this study. These previous studies have sampled uncontrolled gowning area and ISO-8 cleanrooms, where no active spacecraft assembly was ongoing. Moreover, each cleanroom is unique, because of factors such as geographical location (Moissl et al., 2007), assembly activities, different decontamination procedures and most importantly, different workers, which are the main source of contamination.

We saw an increased metabolic diversity in samples collected from cleanrooms during spacecraft assembly. Cleanrooms are sometimes referred to as extreme environments; not only due to strict decontamination procedures, but also due to the lack of nutrients, water and cofactors (La Duc et al., 2007; Ghosh et al., 2010). Since there are very few resources to rely on in an area that is maintained to be uninhabitable, any microbes able to survive here would have to synthesize all necessary factors themselves. Sterilization procedures and gowning requirements are even stricter during assembly, making it even harder for microorganisms to survive. Strict gowning protocols and increased frequency of cleaning leads to decrease in introduction of human associated microbes, despite high human activities in the cleanroom during assembly. This might also explain lower phylogenetic diversity during Phoenix spacecraft assembly compared to before or after assembly. In addition to the decreased phylogenetic diversity, pathogenic diversity was also lower during spacecraft assembly, however, we observed an increase in pathogen abundance over time. This suggests that strict decontamination procedure favor the growth of pathogens. Nevertheless, studies with bigger sample sizes need to confirm our descriptive findings. A considerable amount, in case of MSL more than 50%, of all reads was assigned to genetic information processing. This highlights the importance of genetic information processing, including DNA repair in such a harsh environment. Surprisingly, microbial profiles during assembly were very similar. Although, DAWN and MSL samples were collected from geographically distinct locations, they were more similar to PHX-D than PHX-B or PHX-A. This suggests that decontamination procedures have a bigger effect on the cleanroom microbiome than location. Taken together, these results show that decontamination and gowning measures were not only sufficient, but also well executed.

Most virulence factors are organized in so-called pathogenicity-islands (Schmidt and Hensel, 2004). Commensals can turn into pathogens by acquiring pathogenicity-island through phages, or horizontal gene transfer. For example, wild type *Vibrio cholerae* are not able to cause deadly diarrhea. Only upon infection by the CTX prophage they acquire a pathogenicity island coding for virulence factors, such as the cholera toxin and pili, needed for recognition host and disease induction (Das et al., 2011). Therefore, virulence factor detection is the only reliable method to identify pathogens.

*Acinetobacter baumannii, Acinetobacter lwoffii, Escherichia coli and Legionella pneumophila* were found in all samples, although samples were collected from three geographically distinct sites. These prevalent pathogens have to be very resistant to overcome all the cleaning and decontamination procedures. *Acinetobacter* spp., such as *A. baumannii* and *A. lwoffii* are non-fastidious and can rely on a single energy source from different substrates (Rathinavelu et al., 2003). They are resistant to radiation (Firstenberg-Eden et al., 1980b) and several disinfectants and can survive in a wide range of temperatures (Firstenberg-Eden et al., 1980a) and even in low pH. These features might explain why *Acinetobacter* spp. were the most dominating species during spacecraft assembly in this study. *Acinetobacter* spp. have also been reported in high abundance in cleanrooms in previous studies (Vaishampayan et al., 2010a; Mahnert et al., 2015). *Acinetobacter baumannii* has been isolated from water and soil (Yeom et al., 2013), but it has also been found in other hostile environments such as intensive care units. Although *A. baumannii* is not pathogenic to healthy individuals, it can be an opportunistic pathogen in immunocompromised patients. Hence, it is one of the ESKAPE pathogens (Boucher et al., 2009), which are multidrug-resistant bacteria, responsible for the majority of nosocomial infections (Rice, 2008).

We found *A. baumannii* specific beta-Lactamase TEM-1, AdeABC and another cation/multidrug efflux pump, which might be responsible for *A. baumannii's* resistance against all decontamination measures. AdeABC alone is responsible for resistance to aminoglycosides, tetracyclines, erythromycin, chloramphenicol, trimethoprim, fluoroquinolones, some beta-lactams, and also recently tigecycline since they have been described as substrates for this multidrug efflux pump (Wieczorek et al., 2008). We did not detect *A. Iwoffii* associated potential virulence factors in our data set. MvirDB has only three *A. Iwoffii* (formerly known as *Acinetobacter calcoaceticus*) associated virulence factors (two beta-lactamases and a chloramphenicol acetyl transferase). Nevertheless, the presence of this opportunistic pathogen in all our sample collection from locations separated by hundreds of miles, its resistant features, and our finding that *Acinetobacter* spp. were dominating in all three locations during assembly, suggests that *A. lwoffii* and *A. baumannii* are actually viable in the spacecraft cleanroom environment. *L. pneumophila*, another pathogen present in all three distinct locations, is the causative agent of the Legionnaires' disease (Shevchuk et al., 2011), with symptoms such as fever, chills, and coughing. We found Legionella secretion pathway protein E (LspE), which is part of a type II secretion system required for its full virulence and environmental persistence (Hales and Shuman, 1999). In addition, other *L. pneumophila* associated virulence factors, such as the catalase-peroxidase KatB and superoxide dismutase were present, potentially explaining why this species is resistant to hydrogen peroxide treatment, one of the decontamination procedures. The last potential pathogen we found in all cleanrooms was *E. coli*. Although, we have found several virulence factors such as transposases and antimicrobial resistance genes, we cannot confirm whether or not this specific *E. coli* is a pathogen, given that more and more antimicrobial resistance genes are being found in commensal *E. coli* (Kaesbohrer et al., 2012; Tadesse et al., 2012; Wasyl et al., 2013). While we think that the four pathogens found in all geographically separated cleanrooms are alive, given their resistant features, we are not able to tell if the classified taxa and functions derive from intact living cells or if they are originating from dead cells. In an ongoing study we're including propidium monoazide staining, enabling us to differentiate between sequences coming from intact live and dead microorganisms.

Interestingly, potential virulence factor abundance increased over time, despite lower phylogenetic diversity during assembly. We have concluded that virulence factors may provide a survival advantage in this very hostile environment (Rathinavelu et al., 2003). Multidrug efflux pumps might be pumping out harmful compounds before they are able to execute their deadly effect (Yoon et al., 2013). This virulence factor accumulation seems to be species dependent, as we also see an increase in pathogenic abundance over time. We also found pathogens not belonging to the bacterial kingdom; such as *Candida parapsilosis*, a fungus, which plays an important role in wound and tissue sepsis of immunocompromised patients and makes up to 15% of all *Candida* infections.

One limitation of this study is the low biomass in cleanroom samples, due to the repeated strict cleaning and decontamination practices. MDA was necessary to acquire DNA concentrations sufficient for library preparation. MDA can introduce bias, by favoring some DNA fragments over others (Direito et al., 2014). Therefore, some microorganisms might not have been detected in our approach, while others might be overrepresented. Although, we performed stringent quality filtering of our reads, it's impossible to get rid of all errors and biases. Homology based approaches such as BLASTx against specialized databases such as MvirDB are biased, because a sequence with an 80% sequence similarity might have a better hit to a reference which is not in the database. However, we searched all positive MvirDB hits against NCBI non-redundant database, and the majority was classified as virulence factors (see Presentation S1 in Supplementary Material). Moreover, the circumstantial evidences of the presence of virulence factors associated with human pathogens in cleanroom samples should be confirmed by implementation of selective cultivation based approaches and viability-based molecular assays in future missions.

Humans spend most of their lives indoors (Höppe and Martinac, 1998). Recent studies have speculated that human microbiome is the major contributor to the overall indoor microbiome (Lax et al., 2014). Stringent cleaning and maintenance practices in highly controlled indoor environments such as cleanrooms, hospitals and intensive care units may lead to a relative increase of human pathogens in these environments. This may have serious impact on health of the inhabitants. Monitoring pathogens and virulence factors in these indoor environments may prevent diseases such as nosocomial infections and sustain human health.

The results of this study could be used to develop fast and cost-efficient tests (Craw et al., 2015) to detect the presence of specific pathogens or their virulence factors in enclosed environments such as public transport, pharmaceutical cleanrooms, hospitals, and intensive care units. This study has broadened our understanding of the role of pathogens in such highly controlled environments and should be considered for microbial monitoring of the ISS during sustained presence of humans in space and future manned missions to Mars.

## Author contributions

Designed project: PV. Performed wet or computational experiments: MB, PV, DC and NI. Analyzed data: MB, NI and TW. Drafting the manuscript: MB and MA. Generated figures and tables: MB and MA. Wrote and critically reviewed the manuscript: all authors.

### Conflict of interest statement

The authors declare that the research was conducted in the absence of any commercial or financial relationships that could be construed as a potential conflict of interest.
