# Supplementary material for: Functional Metagenomics of Spacecraft Assembly Cleanrooms: Presence of Virulence Factors Associated with Human Pathogens
Source: Front Microbiol. 2016 Sep 9;7:1321. doi: 10.3389/fmicb.2016.01321 (PMC5017214; doi:10.3389/fmicb.2016.01321)
Supplement: Supplementary file 1 [file Table1.DOCX]

Supplementary Material

New perspective on cleanrooms and human pathogens

**Mina Bashir^1,2^, Mahjabeen Ahmed^1,3^, Thomas Weinmaier^4^, Natalia Ivanova^5^, Thomas R. Pieber^2^, Parag Vaishampayan^1*^**

*** Correspondence:** Parag Vaishampayan: Parag.A.Vaishampayan@jpl.nasa.gov

Table 1: List of contaminant genomes used to exclude common contaminants

| gi\|93352797\|gb\|CP000352.1\| Cupriavidus metallidurans CH34, complete genome 5s, 16s, 23s masked |
| --- |
| gi\|160361034\|gb\|CP000884.1\| Delftia acidovorans SPH-1, complete genome 5s, 16s, 23s masked |
| gi\|253972022\|gb\|CP000819.1\| Escherichia coli B str. REL606, complete genome 5s, 16s, 23s masked |
| NC_010473 gi\|170079663\|ref\|NC_010473.1\| Escherichia coli str. K-12 substr. DH10B, complete genome 5s, 16s, 23s masked |
| gi\|206564770\|gb\|CP000964.1\| Klebsiella pneumoniae 342, complete genome 5s, 16s, 23s masked |
| gi\|57158257\|dbj\|AP006725.1\| Klebsiella pneumoniae subsp. pneumoniae NTUH-K2044 DNA, complete genome 5s, 16s, 23s masked |
| gi\|218888746\|ref\|NC_011770.1\| Pseudomonas aeruginosa LESB58, complete genome 5s, 16s, 23s masked |
| gi\|152983466\|ref\|NC_009656.1\| Pseudomonas aeruginosa PA7, complete genome 5s, 16s, 23s masked |
| gi\|110645304\|ref\|NC_002516.2\| Pseudomonas aeruginosa PAO1 chromosome, complete genome 5s, 16s, 23s masked |
| gi\|116048575\|ref\|NC_008463.1\| Pseudomonas aeruginosa UCBPP-PA14, complete genome 5s, 16s, 23s masked |
| gi\|104779316\|ref\|NC_008027.1\| Pseudomonas entomophila L48 chromosome, complete genome 5s, 16s, 23s masked |
| gi\|255961261\|ref\|NC_007492.2\| Pseudomonas fluorescens Pf0-1, complete genome 5s, 16s, 23s masked |
| gi\|229587578\|ref\|NC_012660.1\| Pseudomonas fluorescens SBW25 chromosome, complete genome 5s, 16s, 23s masked |
| gi\|70728250\|ref\|NC_004129.6\| Pseudomonas fluorescens Pf-5 chromosome, complete genome 5s, 16s, 23s masked |
| gi\|146305042\|ref\|NC_009439.1\| Pseudomonas mendocina ymp, complete genome 5s, 16s, 23s masked |
| gi\|148545259\|ref\|NC_009512.1\| Pseudomonas putida F1 chromosome, complete genome 5s, 16s, 23s masked |
| gi\|166857509\|gb\|CP000926.1\| Pseudomonas putida GB-1, complete genome 5s, 16s, 23s masked |
| gi\|26986745\|ref\|NC_002947.3\| Pseudomonas putida KT2440 chromosome, complete genome 5s, 16s, 23s masked |
| gi\|170719187\|ref\|NC_010501.1\| Pseudomonas putida W619 chromosome, complete genome 5s, 16s, 23s masked |
| gi\|146280397\|ref\|NC_009434.1\| Pseudomonas stutzeri A1501, complete genome 5s, 16s, 23s masked |
| gi\|71553748\|gb\|CP000058.1\| Pseudomonas syringae pv. phaseolicola 1448A, complete genome 5s, 16s, 23s masked |
| gi\|66043271\|ref\|NC_007005.1\| Pseudomonas syringae pv. syringae B728a, complete genome 5s, 16s, 23s masked |
| gi\|28856110\|gb\|AE016853.1\| Pseudomonas syringae pv. tomato str. DC3000, complete genome 5s, 16s, 23s masked |
| gi\|71733195\|ref\|NC_005773.3\| Pseudomonas syringae pv. phaseolicola 1448A chromosome, complete genome 5s, 16s, 23s masked |
| gi\|240863652\|gb\|CP001644.1\| Ralstonia pickettii 12D chromosome 1, complete sequence 5s, 16s, 23s masked |
| gi\|240867064\|gb\|CP001645.1\| Ralstonia pickettii 12D chromosome 2, complete sequence 5s, 16s, 23s masked |
| gi\|240868673\|gb\|CP001647.1\| Ralstonia pickettii 12D plasmid pRp12D02, complete sequence |
| gi\|187724002\|gb\|CP001068.1\| Ralstonia pickettii 12J chromosome 1, complete sequence 5s, 16s, 23s masked |
| gi\|30407127\|emb\|AL646052.1\| Ralstonia solanacearum GMI1000 chromosome complete sequence 5s, 16s, 23s masked |
| gi\|81239530\|gb\|CP000034.1\| Shigella dysenteriae Sd197, complete genome 5s, 16s, 23s masked |
| gi\|73854091\|gb\|CP000038.1\| Shigella sonnei Ss046, complete genome 5s, 16s, 23s masked |
| gi\|37509034\|dbj\|BA000037.2\| Vibrio vulnificus YJ016 DNA, chromosome I, complete sequence 5s, 16s, 23s masked |
| gi\|37595821\|ref\|NC_005128.1\| Vibrio vulnificus YJ016 plasmid pYJ016, complete sequence |

Table 2: List of clinically relevant pathogens used for pathogen search

Acinetobacter baumannii, Acinetobacter lwoffii, Acinetobacter spp., Actinomycetes, Adenovirus, Aeromonas spp., Alcaligenes faecalis, Alcaligenes spp./Achromobacter spp., Alcaligenes xylosoxidans, Arbovirus, Aspergillus spp., Astrovirus, Bacillus anthracis, Bacillus cereus, Bacteriodes fragilis, Bartonella quintana, Bordetella pertussis, Borrelia burgdorferi, Borrelia recurrentis, Brevundimonas diminuta, Brevundimonas vesicularis, Brucella spp., Burkholderia cepacia, Burkholderia mallei, Burkholderia pseudomallei, Campylobacter jejuni / coli, Candida albicans, Candida krusei, Candida parapsilosis, Chikungunya virus, CHIKV, Chlamydia pneumoniae, Chlamydia psittaci, Chlamydia trachomatis, Citrobacter spp., Clostridium botulinum, Clostridium difficile, Clostridium perfringens, Clostridium tetani, Coronavirus, Corynebacterium diphtheriae, Corynebacterium pseudotuberculosis, Corynebacterium spp., Corynebacterium ulcerans, Coxiella burnetii, Coxsackievirus, Crimean-Congo haemorrhagic fever virus, Cryptococcus neoformans, Cryptosporidium hominis, Cryptosporidium parvum, Cyclospora cayetanensis, Cytomegalovirus, Dengue virus, Ebola virus, Echovirus, Entamoeba histolytica, Enterobacter aerogenes, Enterobacter cloacae, Enterococcus faecalis, Enterococcus faecium, Enterococcus hirae, Epidermophyton spp., Epstein-Barr virus, EBV, Escherichia coli, Foot-and-mouth disease virus, FMDV, Francisella tularensis, Giardia lamblia, Haemophilus influenzae, Hantavirus, Helicobacter pylori, Helminths, Hepatitis A virus, HAV, Hepatitis B virus, HBV, Hepatitis C virus, HCV, Hepatitis D virus, Hepatitis E virus, Herpes simplex virus, HSV, Histoplasma capsulatum, Human enterovirus 71, Human herpesvirus 6, HHV-6, Human herpesvirus 7, HHV-7, Human herpesvirus 8, HHV-8, Human immunodeficiency virus, HIV, Human metapneumovirus, Human papillomavirus, Influenza virus, Klebsiella granulomatis, Klebsiella oxytoca, Klebsiella pneumoniae, Lassa virus, Leclercia adecarboxylata, Legionella pneumophila, Leishmania spp., Leptospira interrogans, Leuconostoc pseudomesenteroides, Listeria monocytogenes, Marburg virus, Measles virus, Micrococcus luteus, Microsporum spp., Molluscipoxvirus, Morganella spp., Mumps virus, Mycobacterium chimaera Myco, Mycobacterium leprae Myco, Mycobacterium tuberculosis, Mycoplasma pneumoniae, Neisseria meningitidis, Neisseria gonorrhoeae, Norovirus, Orientia tsutsugamushi, Pantoea agglomerans, Parainfluenza virus, Parvovirus, Pediculus humanus capitis, Pediculus humanus corporis, Plasmodium spp., Pneumocystis jiroveci, Poliovirus, Polyomavirus, Proteus mirabilis, Proteus vulgaris, Providencia rettgeri, Providencia stuartii, Pseudomonas aeruginosa, Pseudomonas spp., Rabies virus, Ralstonia spp., Respiratory syncytial virus, RSV, Rhinovirus, Rickettsia prowazekii, Rickettsia typhi, Rotavirus, Rubella virus, Salmonella enteritidis, Salmonella paratyphi, Salmonella spp., Salmonella typhimurium, Sarcoptes scabiei, Sapovirus, Serratia marcescens, Shigella sonnei, Sphingomonas species, Staphylococcus aureus, Staphylococcus capitis, Staphylococcus epidermidis, Staphylococcus haemolyticus, Staphylococcus hominis, Staphylococcus lugdunensis, Staphylococcus saprophyticus, Stenotrophomonas maltophilia, Streptococcus pneumoniae, Streptococcus pyogenes, Streptococcus spp., TBE virus, Toxoplasma gondii, Treponema pallidum, Trichinella spiralis, Trichomonas vaginalis, Trichophyton spp., Trichosporon spp., Trypanosoma brucei gambiense, Trypanosoma brucei rhodesiense, Trypanosoma cruzi, Vaccinia virus, Varicella zoster virus, Variola virus, Vibrio cholerae, West Nile virus, Yellow fever virus, Yersinia enterocolitica, Yersinia pestis, Yersinia pseudotuberculosis, Zika virus

Table 3: Virulence Factors matching the classified pathogens

| Virulence factor | PHX-B | PHX-D | PHX-A | DAWN | MSL |
| --- | --- | --- | --- | --- | --- |
| 114\|vfid\|115\|vsiid\|114\|ssid\|transposase [Escherichia coli] | 1 | 130 | 5 | 0 | 0 |
| 13827\|vfid\|22101\|vsiid\|42154\|ssid\|ORF B protein [Escherichia coli] | 1 | 0 | 0 | 0 | 330 |
| 13876\|vfid\|22199\|vsiid\|42301\|ssid\|putative reverse transcriptase [Escherichia coli] | 1 | 0 | 0 | 0 | 0 |
| 14026\|vfid\|22499\|vsiid\|42751\|ssid\|putative lysil-tR0 synthetase LysU [Escherichia coli] | 1 | 135 | 0 | 0 | 0 |
| 16399\|vfid\|37388\|vsiid\|58225\|ssid\|hypothetical protein c5198 [Escherichia coli CFT073] | 6 | 0 | 0 | 0 | 0 |
| 16400\|vfid\|37390\|vsiid\|58227\|ssid\|transposase insD [Escherichia coli CFT073] | 1 | 0 | 0 | 0 | 0 |
| 16653\|vfid\|37896\|vsiid\|58733\|ssid\|DLP12 prophage; predicted murein endopeptidase [Escherichia coli str K-12 substr MG1655] | 1 | 0 | 0 | 0 | 0 |
| 16692\|vfid\|37974\|vsiid\|58811\|ssid\|unknown protein encoded by IS2 [Escherichia coli O157:H7 EDL933] | 2 | 0 | 0 | 0 | 0 |
| 16718\|vfid\|38026\|vsiid\|58863\|ssid\|putative resolvase [Escherichia coli O157:H7 EDL933] | 1 | 0 | 0 | 0 | 0 |
| 558\|vfid\|559\|vsiid\|558\|ssid\|transposase TnpA [Escherichia coli] | 1 | 0 | 5217 | 0 | 0 |
| 7787\|vfid\|9499\|vsiid\|11548\|ssid\|alginate o-acetyltransferase AlgI [Pseudomonas aeruginosa PAO1] | 2 | 0 | 1 | 0 | 0 |
| 8173\|vfid\|10259\|vsiid\|12686\|ssid\|endopeptidase Clp ATP-binding chain C [Listeria monocytogenes EGD-e] | 5 | 7 | 11 | 0 | 0 |
| 961\|vfid\|962\|vsiid\|961\|ssid\|transposase [Pseudomonas aeruginosa] | 1 | 33 | 0 | 0 | 0 |
| 13271\|vfid\|20471\|vsiid\|39961\|ssid\|aldehyde dehydrogenase [Vibrio cholerae O1 biovar El Tor str N16961] | 0 | 1 | 1 | 0 | 0 |
| 136\|vfid\|137\|vsiid\|136\|ssid\|TnpA [Escherichia coli] | 0 | 265 | 7 | 0 | 0 |
| 13942\|vfid\|22331\|vsiid\|42499\|ssid\|hypothetical protein [Escherichia coli] | 0 | 167 | 0 | 0 | 0 |
| 13943\|vfid\|22333\|vsiid\|42502\|ssid\|hypothetical protein [Escherichia coli] | 0 | 12 | 0 | 0 | 0 |
| 13944\|vfid\|22335\|vsiid\|42505\|ssid\|hypothetical protein [Escherichia coli] | 0 | 3 | 0 | 0 | 0 |
| 14086\|vfid\|22620\|vsiid\|42932\|ssid\|bifunctional enterobactin receptor/adhesin protein [Escherichia coli CFT073] | 0 | 4 | 0 | 0 | 0 |
| 14168\|vfid\|22786\|vsiid\|43180\|ssid\|ClpV1 [Pseudomonas aeruginosa PAO1] | 0 | 5 | 0 | 0 | 0 |
| 16423\|vfid\|37436\|vsiid\|58273\|ssid\|putative iron-regulated outer membrane virulence protein [Escherichia coli CFT073] | 0 | 2 | 0 | 0 | 0 |
| 16472\|vfid\|37534\|vsiid\|58371\|ssid\|hypothetical protein c2399 [Escherichia coli CFT073] | 0 | 11 | 0 | 0 | 0 |
| 16608\|vfid\|37806\|vsiid\|58643\|ssid\|IS30 transposase [Escherichia coli str K-12 substr MG1655] | 0 | 1 | 0 | 0 | 0 |
| 16612\|vfid\|37814\|vsiid\|58651\|ssid\|putative amino acid/amine transport protein [Escherichia coli K12] | 0 | 2 | 0 | 0 | 0 |
| 16712\|vfid\|38014\|vsiid\|58851\|ssid\|hypothetical protein Z5879 [Escherichia coli O157:H7 EDL933] | 0 | 2 | 0 | 0 | 0 |
| 16850\|vfid\|38289\|vsiid\|59126\|ssid\|putative phage inhibition, colicin resistance and tellurite resistance protein [Escherichia coli O157:H7 EDL933] | 0 | 6 | 6 | 0 | 0 |
| 16853\|vfid\|38295\|vsiid\|59132\|ssid\|putative phage inhibition, colicin resistance and tellurite resistance protein [Escherichia coli O157:H7 EDL933] | 0 | 1 | 0 | 0 | 0 |
| 16856\|vfid\|38301\|vsiid\|59138\|ssid\|hypothetical protein Z1608 [Escherichia coli O157:H7 EDL933] | 0 | 3 | 0 | 0 | 0 |
| 16877\|vfid\|38343\|vsiid\|59180\|ssid\|putative urease accessory protein G [Escherichia coli O157:H7 EDL933] | 0 | 52 | 6 | 0 | 0 |
| 16882\|vfid\|38353\|vsiid\|59190\|ssid\|urease subunit gamma [Escherichia coli O157:H7 EDL933] | 0 | 2 | 2 | 0 | 0 |
| 21131\|vfid\|52490\|vsiid\|73357\|ssid\|aminoglycoside 6`-N-acetyltransferase Iv [Acinetobacter sp 631] | 0 | 1 | 0 | 0 | 0 |
| 21358\|vfid\|52928\|vsiid\|73795\|ssid\|AdeA membrane fusion protein [Acinetobacter baumannii] | 0 | 17 | 0 | 0 | 0 |
| 21359\|vfid\|52930\|vsiid\|73797\|ssid\|AdeA membrane fusion protein [Acinetobacter baumannii] | 0 | 5 | 0 | 0 | 0 |
| 21360\|vfid\|52932\|vsiid\|73799\|ssid\|AdeB [Acinetobacter baumannii] | 0 | 369 | 0 | 0 | 0 |
| 21361\|vfid\|52934\|vsiid\|73801\|ssid\|AdeB RND protein [Acinetobacter baumannii] | 0 | 3 | 0 | 0 | 0 |
| 21362\|vfid\|52936\|vsiid\|73803\|ssid\|AdeC outer membrane protein [Acinetobacter baumannii] | 0 | 1 | 0 | 0 | 0 |
| 22924\|vfid\|55916\|vsiid\|76783\|ssid\|cation/multidrug efflux pump [Acinetobacter baumannii] | 0 | 188 | 7 | 0 | 0 |
| 24250\|vfid\|58435\|vsiid\|79302\|ssid\|RND efflux transporter [Pseudomonas aeruginosa PA7] | 0 | 55 | 19 | 0 | 0 |
| 24251\|vfid\|58437\|vsiid\|79304\|ssid\|RND efflux transporter [Pseudomonas aeruginosa UCBPP-PA14] | 0 | 3 | 0 | 0 | 0 |
| 24958\|vfid\|59824\|vsiid\|80691\|ssid\|Putative tetA efflux pump [Acinetobacter baumannii] | 0 | 9 | 0 | 0 | 0 |
| 452\|vfid\|453\|vsiid\|452\|ssid\|TnpA [Escherichia coli] | 0 | 66 | 3 | 0 | 0 |
| 7250\|vfid\|8385\|vsiid\|10303\|ssid\|Biopolymer transport exbB protein [Escherichia coli CFT073] | 0 | 6 | 6 | 0 | 0 |
| 7402\|vfid\|8690\|vsiid\|10608\|ssid\|twitching motility protein PilT [Pseudomonas aeruginosa PAO1] | 0 | 66 | 0 | 0 | 0 |
| 7403\|vfid\|8692\|vsiid\|10610\|ssid\|twitching motility protein PilU [Pseudomonas aeruginosa PAO1] | 0 | 22 | 0 | 0 | 0 |
| 7404\|vfid\|8694\|vsiid\|10612\|ssid\|twitching motility protein PilG [Pseudomonas aeruginosa PAO1] | 0 | 4 | 0 | 0 | 0 |
| 7407\|vfid\|8700\|vsiid\|10618\|ssid\|twitching motility protein PilJ [Pseudomonas aeruginosa PAO1] | 0 | 9 | 0 | 0 | 0 |
| 7412\|vfid\|8710\|vsiid\|10628\|ssid\|type 4 fimbrial biogenesis protein PilB [Pseudomonas aeruginosa PAO1] | 0 | 6 | 0 | 0 | 0 |
| 7416\|vfid\|8718\|vsiid\|10636\|ssid\|two-component response regulator PilR [Pseudomonas aeruginosa PAO1] | 0 | 1 | 0 | 0 | 0 |
| 7424\|vfid\|8734\|vsiid\|10652\|ssid\|Type 4 fimbrial biogenesis outer membrane protein PilQ precursor [Pseudomonas aeruginosa PAO1] | 0 | 1 | 0 | 0 | 0 |
| 7813\|vfid\|9551\|vsiid\|11626\|ssid\|ferripyoverdine receptor [Pseudomonas aeruginosa PAO1] | 0 | 1080 | 0 | 0 | 1 |
| 7827\|vfid\|9581\|vsiid\|11672\|ssid\|general secretion pathway protein E [Pseudomonas aeruginosa PAO1] | 0 | 60 | 0 | 0 | 0 |
| 8350\|vfid\|10572\|vsiid\|13135\|ssid\|chaperonin GroEL [Legionella pneumophila subsp. pneumophila str Philadelphia 1] | 0 | 13 | 0 | 0 | 0 |
| 8399\|vfid\|10670\|vsiid\|13282\|ssid\|type II protein secretion ATPase LspE [Legionella pneumophila subsp. pneumophila str Philadelphia 1] | 0 | 7 | 0 | 0 | 0 |
| 8459\|vfid\|10790\|vsiid\|13462\|ssid\|catalase-peroxidase KatB [Legionella pneumophila subsp. pneumophila str Philadelphia 1] | 0 | 8 | 1 | 0 | 0 |
| 8460\|vfid\|10792\|vsiid\|13465\|ssid\|superoxide dismutase [Legionella pneumophila subsp. pneumophila str Philadelphia 1] | 0 | 1 | 0 | 0 | 0 |
| 864\|vfid\|865\|vsiid\|864\|ssid\|tnpR [Pseudomonas aeruginosa] | 0 | 22 | 0 | 0 | 0 |
| 14055\|vfid\|22557\|vsiid\|42838\|ssid\|hypothetical protein c3561 [Escherichia coli CFT073] | 0 | 0 | 4 | 0 | 0 |
| 16173\|vfid\|36936\|vsiid\|57773\|ssid\|putative tail component of prophage [Escherichia coli CFT073] | 0 | 0 | 1 | 0 | 0 |
| 16179\|vfid\|36948\|vsiid\|57785\|ssid\|putative tail component of prophage [Escherichia coli CFT073] | 0 | 0 | 2 | 0 | 0 |
| 16196\|vfid\|36982\|vsiid\|57819\|ssid\|cold shock-like protein cspI [Escherichia coli CFT073] | 0 | 0 | 95 | 0 | 0 |
| 16616\|vfid\|37822\|vsiid\|58659\|ssid\|IS1 transposase B [Escherichia coli str K-12 substr MG1655] | 0 | 0 | 1 | 0 | 0 |
| 16625\|vfid\|37840\|vsiid\|58677\|ssid\|ornithine carbamoyltransferase 2, chain F; CP4-6 prophage [Escherichia coli str K-12 substr MG1655] | 0 | 0 | 1 | 0 | 0 |
| 16721\|vfid\|38032\|vsiid\|58869\|ssid\|hypothetical protein Z5888 [Escherichia coli O157:H7 EDL933] | 0 | 0 | 1 | 0 | 0 |
| 21217\|vfid\|52650\|vsiid\|73517\|ssid\|kanamycin nucleotidyltransferase [Staphylococcus aureus subsp aureus Mu50] | 0 | 0 | 1 | 0 | 0 |
| 22715\|vfid\|55533\|vsiid\|76400\|ssid\|bleomycin resistance protein [Staphylococcus aureus subsp aureus N315] | 0 | 0 | 4 | 0 | 0 |
| 24255\|vfid\|58445\|vsiid\|79312\|ssid\|COG0841: Cation/multidrug efflux pump [Pseudomonas aeruginosa C3719] | 0 | 0 | 2 | 0 | 0 |
| 24268\|vfid\|58471\|vsiid\|79338\|ssid\|multidrug efflux protein [Pseudomonas aeruginosa PA7] | 0 | 0 | 5 | 0 | 0 |
| 384\|vfid\|385\|vsiid\|384\|ssid\|nonmetallocarbapenamase regulator [Enterobacter cloacae] | 0 | 0 | 2 | 0 | 0 |
| 480\|vfid\|481\|vsiid\|480\|ssid\|Beta-lactamase [Escherichia coli] | 0 | 0 | 2 | 0 | 0 |
| 7698\|vfid\|9291\|vsiid\|11222\|ssid\|glycerol ester hydrolase [Staphylococcus aureus subsp aureus MW2] | 0 | 0 | 1 | 0 | 0 |
| 788\|vfid\|789\|vsiid\|788\|ssid\|SHV-5a [Klebsiella pneumoniae] | 0 | 0 | 1 | 0 | 0 |
| 7931\|vfid\|9789\|vsiid\|11984\|ssid\|UDP-glucose pyrophosphorylase [Streptococcus pyogenes M1 GAS] | 0 | 0 | 11 | 0 | 0 |
| 794\|vfid\|795\|vsiid\|794\|ssid\|ES-beta-lactamase [Escherichia coli] | 0 | 0 | 4 | 0 | 0 |
| 800\|vfid\|801\|vsiid\|800\|ssid\|ES-beta-lactamase [Klebsiella pneumoniae] | 0 | 0 | 1 | 0 | 0 |
| 8175\|vfid\|10263\|vsiid\|12692\|ssid\|ATP-dependent Clp protease proteolytic subunit [Listeria monocytogenes EGD-e] | 0 | 0 | 2 | 0 | 0 |
| 832\|vfid\|833\|vsiid\|832\|ssid\|beta-lactamase TEM-1 [Acinetobacter baumannii] | 0 | 0 | 18 | 0 | 0 |
| 8350\|vfid\|10572\|vsiid\|13135\|ssid\|chaperonin GroEL [Legionella pneumophila subsp pneumophila str Philadelphia 1] | 0 | 0 | 2 | 0 | 0 |
| 8460\|vfid\|10792\|vsiid\|13465\|ssid\|superoxide dismutase [Legionella pneumophila subsp pneumophila str Philadelphia 1] | 0 | 0 | 1 | 0 | 0 |
| 912\|vfid\|913\|vsiid\|912\|ssid\|class A beta-lactamase TEM-106 [Escherichia coli] | 0 | 0 | 1 | 0 | 0 |
| 927\|vfid\|928\|vsiid\|927\|ssid\|inhibitor-resistant beta-lactamase TEM-79 [Escherichia coli] | 0 | 0 | 1 | 0 | 0 |
| 959\|vfid\|960\|vsiid\|959\|ssid\|beta-lactamase class A [Klebsiella pneumoniae] | 0 | 0 | 1 | 0 | 0 |
| 973\|vfid\|974\|vsiid\|973\|ssid\|beta-lactamase [Enterobacter aerogenes] | 0 | 0 | 1 | 0 | 0 |
| 114\|vfid\|115\|vsiid\|114\|ssid\|transposase.[Escherichia.coli] | 0 | 0 | 0 | 1 | 0 |
| 136\|vfid\|137\|vsiid\|136\|ssid\|TnpA.[Escherichia.coli] | 0 | 0 | 0 | 2 | 0 |
| 13942\|vfid\|22331\|vsiid\|42499\|ssid\|hypothetical.protein.[Escherichia.coli] | 0 | 0 | 0 | 1 | 0 |
| 21360\|vfid\|52932\|vsiid\|73799\|ssid\|AdeB.[Acinetobacter.baumannii] | 0 | 0 | 0 | 3 | 0 |
| 452\|vfid\|453\|vsiid\|452\|ssid\|TnpA.[Escherichia.coli] | 0 | 0 | 0 | 1 | 0 |
| 7403\|vfid\|8692\|vsiid\|10610\|ssid\|twitching.motility.protein.PilU.[Pseudomonas.aeruginosa.PAO1] | 0 | 0 | 0 | 1 | 0 |
| 7407\|vfid\|8700\|vsiid\|10618\|ssid\|twitching.motility.protein.PilJ.[Pseudomonas.aeruginosa.PAO1] | 0 | 0 | 0 | 1 | 0 |
| 7813\|vfid\|9551\|vsiid\|11626\|ssid\|ferripyoverdine.receptor.[Pseudomonas.aeruginosa.PAO1] | 0 | 0 | 0 | 5 | 0 |
| 864\|vfid\|865\|vsiid\|864\|ssid\|tnpR.[Pseudomonas.aeruginosa] | 0 | 0 | 0 | 1 | 0 |
| 13606\|vfid\|21659\|vsiid\|41491\|ssid\|unknown [Escherichia coli] | 0 | 0 | 0 | 0 | 84 |
| 13828\|vfid\|22103\|vsiid\|42157\|ssid\|ORF A protein [Escherichia coli] | 0 | 0 | 0 | 0 | 78 |
| 13879\|vfid\|22205\|vsiid\|42310\|ssid\|ORF B protein [Escherichia coli] | 0 | 0 | 0 | 0 | 7 |
| 14021\|vfid\|22489\|vsiid\|42736\|ssid\|transposase Orf B, insertion element IS100 [Escherichia coli] | 0 | 0 | 0 | 0 | 1 |
