# Supplementary material for: Functional Metagenomics of Spacecraft Assembly Cleanrooms: Presence of Virulence Factors Associated with Human Pathogens
Source: Front Microbiol. 2016 Sep 9;7:1321. doi: 10.3389/fmicb.2016.01321 (PMC5017214; doi:10.3389/fmicb.2016.01321)
Supplement: Supplementary file 2 [file Presentation1.ZIP › Supplement.SI1.taxonomy.html]

Javascript must be enabled to view this page.

members
magnitude
magnitudeUnassigned
count
unassigned
taxon
rank

PHX-B
PHX-D
PHX-A
DAWN
MSL

node0.members.0.jsnode0.members.2.jsnode0.members.3.js
17462213288902903271173062439646
112301

node1.members.0.jsnode1.members.1.jsnode1.members.2.jsnode1.members.3.jsnode1.members.4.js
80415123751511442
1515702
species

node2.members.2.js
1
45196
species

node3.members.0.jsnode3.members.2.jsnode3.members.3.jsnode3.members.4.js
1539730111
932710
species

node4.members.0.jsnode4.members.2.js
2191
1515703
species

node5.members.0.jsnode5.members.1.jsnode5.members.2.jsnode5.members.3.jsnode5.members.4.js
63662775642202129137231
16012712511
2759
superkingdom

node6.members.0.jsnode6.members.1.jsnode6.members.2.jsnode6.members.3.jsnode6.members.4.js
592731488962431137058
90028120223
33208
kingdom

761
10219
phylum

761
10220
class

761
10221
family

761
10222
genus

node11.members.0.jsnode11.members.4.js
761
10224
species

node12.members.0.js
3492
3
6340
phylum

144
6341
class

144
105387
subclass

144
6342
order

144
51292
family

144
51293
genus

node18.members.0.js
144
283909
species

2022
55824
class

2022
6403
subclass

2022
6406
order

2022
6407
family

2022
6411
genus

node24.members.0.jsnode24.members.1.js
2022
6412
species

node25.members.0.js
2592
3
6447
phylum

721
6448
class

271
216318
superfamily

271
6498
family

271
6499
genus

node30.members.0.jsnode30.members.1.js
271
6500
species

2
977780
superfamily

2
6524
family

2
6525
genus

node34.members.0.js
2
6526
species

42
216260
superfamily

42
69676
family

42
72691
genus

node38.members.0.js
42
225164
species

1
216441
superfamily

1
6521
family

1
6522
genus

node42.members.0.js
1
6523
species

1841
6544
class

1841
6545
subclass

2
106218
order

2
106219
superfamily

2
6566
family

2
186466
genus

node49.members.0.js
2
6573
species

1821
6562
order

1821
98302
superfamily

1821
6563
family

1821
6564
genus

node54.members.0.jsnode54.members.1.js
1821
29159
species

4
10226
phylum

4
10227
genus

node57.members.0.js
4
10228
species

92
10190
phylum

92
44578
class

92
104779
order

92
104780
family

92
104781
genus

node63.members.0.js
92
104782
species

9
6040
phylum

9
6042
class

9
6049
order

9
178475
family

9
178513
genus

node69.members.0.js
9
400682
species

200100
6073
phylum

15100
35583
order

15100
109802
suborder

15100
35587
family

15100
89725
genus

node75.members.0.jsnode75.members.2.js
15100
669202
species

99
6074
class

99
37516
subclass

99
406427
order

99
1612408
suborder

99
6080
family

node81.members.0.js
99
14
6083
genus

node82.members.0.js
73
6087
species

node83.members.0.js
12
42240
species

86
6101
class

86
6102
subclass

86
6103
order

84
45349
family

84
45350
genus

node89.members.0.js
84
45351
species

2
86626
suborder

2
42822
family

2
6104
genus

node93.members.0.js
2
6106
species

node94.members.0.js
360862212313
7
6231
phylum

node95.members.0.js
349562210312
27
119089
class

42241
6274
order

42241
6295
superfamily

42241
6296
family

24
6292
genus

node100.members.1.js
24
6293
species

1
7208
genus

node102.members.0.js
1
7209
species

411
6278
genus

node104.members.0.jsnode104.members.2.js
411
6279
species

6
6249
order

6
33256
superfamily

6
33259
family

6
6264
genus

node109.members.0.js
6
6265
species

node110.members.0.js
342038209312
5
6236
order

node111.members.0.jsnode111.members.1.js
329418202311
3312
6308
suborder

41
6314
superfamily

41
126387
family

41
33260
subfamily

41
6288
genus

node116.members.0.js
41
6289
species

292216202311
33277
superfamily

node118.members.0.jsnode118.members.3.js
292216202311
72
33278
family

304214111
53469
subfamily

node120.members.0.jsnode120.members.3.js
304214111
351
29169
genus

node121.members.0.jsnode121.members.1.jsnode121.members.2.js
852141
51022
species

node122.members.0.jsnode122.members.4.js
1841
53326
species

2611146110
53477
subfamily

2611146110
51030
genus

node125.members.0.jsnode125.members.1.jsnode125.members.2.jsnode125.members.4.js
2611146110
51031
species

1212071
55879
superfamily

1212071
6243
family

1212071
55885
subfamily

node129.members.0.js
1212071
15
6237
genus

node130.members.0.jsnode130.members.2.jsnode130.members.4.js
3771
135651
species

node131.members.0.jsnode131.members.1.js
5920
31234
species

node132.members.0.js
9
6238
species

node133.members.0.js
1
6239
species

10621
119088
class

10621
1457286
subclass

node136.members.0.js
10621
10
6329
order

6121
119093
family

node138.members.0.js
6121
4
36086
genus

node139.members.0.jsnode139.members.2.js
52
36087
species

node140.members.0.jsnode140.members.4.js
521
68888
species

35
6332
family

35
6333
genus

node143.members.0.js
35
6334
species

node144.members.0.jsnode144.members.2.js
71551170575418136789
1222
7711
phylum

481
7735
subphylum

481
7736
family

481
7737
genus

node148.members.0.jsnode148.members.2.js
481
7739
species

23254
7712
subphylum

22
7713
class

22
183770
order

22
7716
suborder

22
7717
family

22
7718
genus

node155.members.0.js
22
7719
species

1254
30302
class

1254
41302
family

1254
34763
genus

node159.members.0.jsnode159.members.2.js
1254
34765
species

node160.members.0.jsnode160.members.1.jsnode160.members.2.jsnode160.members.3.jsnode160.members.4.js
69621170549718136789
74916794102282
89593
subphylum

1332
8504
superorder

1332
8509
order

52
8570
infraorder

44
34989
superfamily

44
8602
family

44
42168
subfamily

44
8664
genus

node168.members.0.js
44
8665
species

8
34979
superfamily

8
34984
family

8
37579
genus

node172.members.0.js
8
176946
species

1
8560
infraorder

1
8561
family

1
385256
subfamily

node176.members.0.js
1
42749
genus

802
8511
suborder

802
8515
family

802
81957
subfamily

802
28376
genus

node181.members.0.jsnode181.members.4.js
802
28377
species

11
7777
class

11
7863
subclass

11
7864
order

11
7865
family

11
7866
genus

node187.members.0.jsnode187.members.4.js
11
7868
species

188
8459
order

188
8464
suborder

48
27791
superfamily

48
8465
family

48
8468
genus

node193.members.0.jsnode193.members.2.js
48
8469
species

10
8486
superfamily

10
8476
family

10
8477
genus

10
8479
species

node198.members.0.js
10
8478
subspecies

4
34907
family

4
204969
genus

node201.members.0.js
4
13735
species

4601
7898
superclass

4601
186623
class

node204.members.0.js
4601
1
41665
subclass

node205.members.0.js
4571
165
32443
infraclass

1
186628
superorder

1
7991
order

1
1489739
suborder

1
7992
family

1
7993
genus

node211.members.0.js
1
7994
species

31
1489910
superorder

31
1489911
order

31
8113
family

node215.members.0.js
31
7
318546
subfamily

1
319069
tribe

1
32506
genus

node218.members.0.js
1
32507
species

node219.members.0.js
6
2
319058
tribe

1
195936
genus

node221.members.0.js
1
303518
species

1
143623
genus

1
57445
species group

node224.members.0.js
1
106582
species

2
8121
genus

node226.members.0.js
2
8153
species

17
1315725
tribe

17
8139
genus

node229.members.0.js
17
8128
species

581
1489913
superorder

581
28738
order

581
8087
suborder

581
8079
family

581
586240
subfamily

30
8082
genus

node236.members.0.js
30
8083
species

node237.members.0.jsnode237.members.1.js
281
91
8080
genus

node238.members.0.js
9
8081
species

node239.members.0.js
10
48698
species

31
31022
order

31
31028
suborder

31
32517
superfamily

31
31031
family

27
31032
genus

node245.members.0.js
27
31033
species

4
47144
genus

node247.members.0.js
4
99883
species

22
8007
order

22
8008
family

22
8009
genus

node251.members.0.js
22
8010
species

98
8006
order

98
8015
family

node254.members.0.js
98
2
504568
subfamily

96
8016
genus

node256.members.0.js
96
8022
species

4
8111
order

4
8205
suborder

4
8206
family

4
8207
genus

node261.members.0.js
4
8208
species

15
30863
family

15
80992
genus

node264.members.0.js
15
144197
species

29
186627
superorder

29
7952
order

29
30727
superfamily

29
7953
family

29
7954
genus

node270.members.0.js
29
7955
species

3
8252
order

3
30942
suborder

3
30947
family

3
603456
subfamily

3
106173
genus

node276.members.0.js
3
244447
species

2
1489100
infraclass

2
7914
order

2
7915
family

2
7916
genus

node281.members.0.js
2
7918
species

1051
7894
order

1051
7895
family

1051
7896
genus

node285.members.0.jsnode285.members.4.js
1051
7897
species

node286.members.0.jsnode286.members.2.js
1899201
41
8782
class

6
8783
superorder

6
8802
order

6
8803
family

6
30467
genus

node291.members.0.js
6
94827
species

node292.members.0.jsnode292.members.2.js
1889191
2991
8825
superorder

1
9214
order

1
9215
family

1
9216
genus

1
9217
species

node297.members.0.js
1
9218
subspecies

492
9219
order

492
9220
family

492
36302
genus

node301.members.0.jsnode301.members.2.js
492
118200
species

17
8940
order

17
8941
family

17
33592
genus

node305.members.0.js
17
55661
species

833
8892
order

833
8893
family

833
8896
genus

node309.members.0.js
833
8897
species

35
30411
order

35
30412
family

35
37038
genus

node313.members.0.js
35
37040
species

17
8906
order

17
8907
family

17
50391
genus

node317.members.0.js
17
50402
species

6
9223
order

6
9224
family

6
176042
genus

node321.members.2.js
6
176057
species

44
9241
order

44
9242
family

44
9243
genus

node325.members.0.js
44
9244
species

852
9108
order

1
9109
family

1
30413
genus

1
925459
species

node330.members.0.js
1
100784
subspecies

792
54372
family

792
54373
genus

node333.members.0.jsnode333.members.2.js
792
54374
species

5
54381
family

5
54382
genus

node336.members.0.js
5
54383
species

node337.members.0.js
39441
14
9126
order

206
192204
superfamily

206
28725
family

206
30420
genus

node341.members.0.js
206
85066
species

221
114313
family

221
196036
genus

node344.members.0.jsnode344.members.2.js
221
328815
species

15231
38633
family

15231
57067
genus

node347.members.0.jsnode347.members.2.jsnode347.members.4.js
15231
57068
species

11
8920
order

1
8922
family

1
33611
genus

node351.members.2.js
1
43455
species

1
56295
family

1
56296
genus

node354.members.0.js
1
240206
species

3
9205
order

2
8899
family

2
56073
genus

node358.members.0.js
2
188379
species

1
33574
family

1
128389
genus

node361.members.0.js
1
128390
species

2
30447
order

2
30448
family

2
37049
genus

node365.members.0.js
2
345573
species

38
57384
order

38
57385
family

38
57409
genus

node369.members.0.js
38
57412
species

2
8826
order

2
8830
family

2
8835
genus

node373.members.0.js
2
8839
species

12
8948
order

12
8949
family

node376.members.0.js
12
1
8952
genus

node377.members.0.js
11
345164
species

561
8929
order

561
8930
family

561
8931
genus

node381.members.0.jsnode381.members.2.js
561
8932
species

12
56301
order

12
56302
family

12
56303
genus

node385.members.0.jsnode385.members.2.js
12
121530
species

341
8292
class

341
41666
superorder

341
8342
order

341
30319
superfamily

341
8352
family

341
8360
subfamily

node392.members.1.js
341
1
8353
genus

2
262014
subgenus

node394.members.0.js
2
8355
species

32
8363
subgenus

node396.members.0.js
32
8364
species

node397.members.0.jsnode397.members.1.jsnode397.members.2.jsnode397.members.3.jsnode397.members.4.js
3505115253901434502
17931274420613
40674
class

2
38605
order

2
9265
family

2
126287
subfamily

2
13615
genus

node402.members.0.js
2
13616
species

6
9348
superorder

6
948951
order

6
9359
family

6
9360
genus

node407.members.0.js
6
9361
species

411
311790
superorder

1
9774
order

1
9775
family

1
9776
genus

1
9778
species

node413.members.0.js
1
127582
subspecies

1
28734
order

1
28735
family

1
28736
genus

node417.members.2.js
1
28737
species

1
9369
family

1
176113
subfamily

1
9370
genus

node421.members.1.js
1
9371
species

3
9389
family

3
745257
subfamily

3
185452
genus

node425.members.0.js
3
185453
species

node426.members.0.jsnode426.members.1.jsnode426.members.2.jsnode426.members.4.js
3088106749591013887
2612030
314146
superorder

node427.members.0.jsnode427.members.2.js
29281063491695718
32
9443
order

node428.members.0.jsnode428.members.2.jsnode428.members.4.js
29241063491495718
431
376913
suborder

node429.members.0.jsnode429.members.1.jsnode429.members.2.jsnode429.members.3.jsnode429.members.4.js
29161063491195717
264716122704
314293
infraorder

node430.members.0.jsnode430.members.1.jsnode430.members.2.jsnode430.members.3.jsnode430.members.4.js
2633991428475009
1690519202253916
9526
parvorder

node431.members.0.jsnode431.members.2.js
72411819171818
1137
314295
superfamily

node432.members.0.jsnode432.members.1.jsnode432.members.2.jsnode432.members.4.js
71011818691818
134511
9604
family

node433.members.0.jsnode433.members.1.jsnode433.members.2.jsnode433.members.4.js
69211318141817
10153759
207598
subfamily

node434.members.0.js
2277735
3
9596
genus

node435.members.0.jsnode435.members.2.js
571
9598
species

node436.members.0.jsnode436.members.2.jsnode436.members.4.js
146735
9597
species

node437.members.0.js
5451011303173
2
9605
genus

node438.members.0.jsnode438.members.1.jsnode438.members.2.jsnode438.members.3.jsnode438.members.4.js
5431011303173
9606
species

24759
9592
genus

24759
9593
species

node441.members.0.jsnode441.members.1.jsnode441.members.2.js
24759
9595
subspecies

514
607660
subfamily

514
9599
genus

node444.members.0.jsnode444.members.1.jsnode444.members.2.js
514
9601
species

node445.members.2.js
311
1
9577
family

310
325165
genus

node447.members.0.jsnode447.members.2.js
310
61853
species

2193543451275
314294
superfamily

node449.members.0.jsnode449.members.2.js
2193543451275
319
9527
family

node450.members.0.jsnode450.members.1.jsnode450.members.2.jsnode450.members.4.js
2023542851274
7219104
9528
subfamily

node451.members.0.jsnode451.members.1.jsnode451.members.2.jsnode451.members.4.js
1795925824
313797
9539
genus

node452.members.0.jsnode452.members.1.jsnode452.members.2.jsnode452.members.4.js
83511187
9541
species

node453.members.0.jsnode453.members.1.jsnode453.members.2.jsnode453.members.4.js
6556110
9544
species

node454.members.0.js
43245
1
392815
genus

node455.members.0.jsnode455.members.2.jsnode455.members.4.js
33245
60711
species

12761411
9554
genus

node457.members.0.jsnode457.members.1.jsnode457.members.2.jsnode457.members.3.jsnode457.members.4.js
12761411
9555
species

14411
9569
subfamily

14411
542827
genus

node460.members.0.jsnode460.members.2.jsnode460.members.4.js
14411
61622
species

191154
9479
parvorder

61
376919
family

61
38069
subfamily

61
9522
genus

node465.members.0.jsnode465.members.1.js
61
9523
species

node466.members.2.js
13154
1
9498
family

634
378850
subfamily

634
9520
genus

634
27679
species

node470.members.0.jsnode470.members.2.jsnode470.members.4.js
634
39432
subspecies

711
9480
subfamily

711
9481
genus

node473.members.0.jsnode473.members.2.js
711
9483
species

4
376912
infraorder

4
9475
family

4
9476
genus

node477.members.0.js
4
9478
species

node478.members.0.js
1
38020
species

node479.members.4.js
12921818137
1
9989
order

node480.members.0.js
143
1
33550
suborder

2
10150
family

2
10151
genus

node483.members.2.js
2
34839
species

1
10139
family

1
10140
genus

node486.members.2.js
1
10141
species

13
10167
family

8
423606
genus

node489.members.0.js
8
885580
species

5
10180
genus

node491.members.0.js
5
10181
species

node492.members.0.jsnode492.members.3.jsnode492.members.4.js
11521518136
311933
33553
suborder

2
55153
family

2
337726
subfamily

2
337730
tribe

2
1141640
genus

node497.members.2.js
2
43179
species

99137
10066
family

node499.members.0.js
99137
1
39107
subfamily

9512
10114
genus

node501.members.0.jsnode501.members.2.js
9512
10116
species

317
10088
genus

317
862507
subgenus

node504.members.0.jsnode504.members.2.jsnode504.members.4.js
317
10090
species

1326196
337677
family

1126196
10026
subfamily

1126196
10028
genus

node508.members.0.jsnode508.members.1.jsnode508.members.4.js
1126196
10029
species

2
337963
subfamily

node510.members.0.js
2
1
10040
genus

1
10042
species

node512.members.0.js
1
230844
subspecies

32
9392
order

32
9393
family

32
9394
genus

node516.members.0.jsnode516.members.4.js
32
246437
species

node517.members.1.jsnode517.members.2.js
115
11
9975
order

1
9979
family

1
9984
genus

node520.members.0.js
1
9986
species

4
9976
family

4
9977
genus

node523.members.2.js
4
9978
species

1
30656
order

1
30657
family

1
482536
genus

node527.members.0.js
1
482537
species

node528.members.0.jsnode528.members.1.jsnode528.members.2.js
226531562
18131
314145
superorder

14116
33554
order

1
379583
suborder

node531.members.0.js
1
9681
family

13116
379584
suborder

11
9709
family

11
9712
genus

node535.members.0.jsnode535.members.2.js
11
9713
species

2
9655
family

2
169418
subfamily

2
9665
genus

2
9668
species

node540.members.2.js
2
9669
subspecies

8
9608
family

8
9611
genus

8
9612
species

node544.members.2.js
8
9615
subspecies

1215
9632
family

1
9639
genus

node547.members.2.js
1
29073
species

1214
9645
genus

node549.members.0.jsnode549.members.1.jsnode549.members.2.js
1214
9646
species

119
9721
order

119
9722
suborder

19
119500
family

19
118796
genus

node554.members.1.jsnode554.members.2.js
19
118797
species

1
9726
family

1
9738
genus

node557.members.0.js
1
9739
species

1834683
9845
suborder

1834683
35500
infraorder

node560.members.0.jsnode560.members.1.jsnode560.members.2.js
1834683
816
9895
family

node561.members.2.js
1731273
4
27592
subfamily

1
9900
genus

1
9901
species

node564.members.2.js
1
43346
subspecies

36
9918
genus

node566.members.0.jsnode566.members.1.js
36
89462
species

node567.members.0.jsnode567.members.1.jsnode567.members.2.js
170668
12429
9903
genus

node568.members.0.jsnode568.members.1.jsnode568.members.2.js
158238
72004
species

node569.members.2.js
1
9913
species

2334
9948
subfamily

2334
59537
genus

node572.members.0.jsnode572.members.1.jsnode572.members.2.js
2334
59538
species

2131
35497
infraorder

2131
9821
family

2131
9822
genus

node576.members.0.jsnode576.members.1.jsnode576.members.2.jsnode576.members.4.js
2131
9823
species

65
9834
suborder

node578.members.2.js
65
1
9835
family

64
9836
genus

node580.members.0.jsnode580.members.2.js
61
419612
species

node581.members.2.js
3
9838
species

1
9362
order

1
9363
family

1
30577
subfamily

1
9364
genus

node586.members.2.js
1
9365
species

1381
9397
order

131
30560
suborder

131
9431
family

131
9434
genus

node591.members.2.js
1
59463
species

node592.members.1.js
3
225400
species

node593.members.0.js
1
109478
species

71
30559
suborder

71
9398
family

71
77225
subfamily

71
9401
genus

node598.members.2.js
7
9402
species

node599.members.4.js
1
132908
species

1
9787
order

1
9788
family

1
9789
genus

1
35510
subgenus

node604.members.0.js
1
9796
species

58
1294634
order

58
1294636
family

58
34915
subfamily

node608.members.0.js
58
4
8495
genus

node609.members.0.js
54
38654
species

node610.members.0.jsnode610.members.1.jsnode610.members.4.js
3846316935368232
2831173
6656
phylum

node611.members.0.js
326046844135
31
6960
superclass

node612.members.0.jsnode612.members.4.js
325736844135
4451
50557
class

154
30264
subclass

node614.members.0.js
154
1
27551
family

5
27552
genus

node616.members.0.js
5
27553
species

148
89054
genus

node618.members.0.js
148
89055
species

3
33339
subclass

1
6961
order

1
6962
suborder

1
70899
superfamily

node623.members.0.js
1
6964
family

node624.members.0.js
2
30073
order

node625.members.0.jsnode625.members.1.jsnode625.members.2.jsnode625.members.4.js
319716844134
675317218
33340
subclass

node626.members.0.js
57434110
9
33341
infraclass

node627.members.0.js
57304110
102
6970
superorder

4842219
85823
order

4842219
1049656
superfamily

7
6979
family

7
76894
subfamily

7
36960
genus

node633.members.0.js
7
36961
species

4835219
1049651
family

4835219
37811
subfamily

4835219
6972
genus

node637.members.0.jsnode637.members.1.jsnode637.members.2.jsnode637.members.4.js
4835219
6973
species

node638.members.0.js
78621
4
7499
order

78121
7501
family

78121
127820
subfamily

78121
127821
tribe

78121
7502
genus

node643.members.0.jsnode643.members.1.jsnode643.members.4.js
78121
136037
species

1
36985
family

1
72653
subfamily

1
36986
genus

node647.members.0.js
1
36987
species

4
6993
order

3
7001
suborder

3
92621
superfamily

3
7002
family

3
63719
subfamily

3
34654
genus

3
34655
species

node655.members.0.js
3
1477514
subspecies

1
6994
suborder

1
70906
superfamily

1
6995
family

1
114953
subfamily

1
82804
genus

node661.members.0.js
1
62746
species

node662.members.0.jsnode662.members.1.jsnode662.members.4.js
17503421386
3155413
33392
infraclass

324568
85604
superorder

324568
7088
order

324568
41191
suborder

324568
41196
infraorder

node667.members.0.jsnode667.members.1.jsnode667.members.4.js
324568
202345
41197
parvorder

8
37581
superfamily

8
32434
family

8
347703
subfamily

8
13190
genus

node672.members.0.js
8
13191
species

35
37570
superfamily

node674.members.0.js
17
2
7100
family

11
95179
subfamily

11
47766
genus

node677.members.0.js
11
215162
species

3
95178
subfamily

2
7101
genus

node680.members.0.js
2
7102
species

1
7112
genus

node682.members.0.js
1
7113
species

1
95244
subfamily

1
55056
genus

node685.members.0.js
1
55057
species

18
27548
family

18
13122
genus

node688.members.0.js
18
13123
species

10
82592
superfamily

10
82593
family

10
82596
subfamily

8
281823
genus

node693.members.0.js
8
722671
species

2
248898
genus

node695.members.0.js
2
248899
species

59
37573
superfamily

59
268499
family

1
72364
subfamily

1
72365
genus

node700.members.0.js
1
72366
species

58
40080
subfamily

58
29056
genus

node703.members.0.js
58
29057
species

2
104423
superfamily

2
104465
subfamily

2
104426
genus

node707.members.0.js
2
998833
species

node708.members.0.js
1511
2
37572
superfamily

node709.members.0.js
1431
2
33415
family

1251
127218
subfamily

1251
30248
tribe

1251
344705
subtribe

1251
13036
genus

1251
151542
subgenus

node715.members.0.jsnode715.members.1.js
1251
13037
species

6
40037
subfamily

6
127322
tribe

6
33416
genus

node719.members.0.js
6
33431
species

10
42282
subfamily

node721.members.0.js
10
2
127320
tribe

4
150878
subtribe

4
111893
genus

node724.members.0.js
4
242261
species

4
150886
subtribe

node726.members.0.js
4
3
100746
genus

node727.members.0.js
1
596624
species

1
124343
family

1
42265
subfamily

1
124360
tribe

1
124361
genus

node732.members.0.js
1
717297
species

5
27544
family

2
42297
subfamily

1
689545
genus

node736.members.0.js
1
689551
species

1
301170
genus

node738.members.0.js
1
203778
species

3
42300
subfamily

3
140315
tribe

3
328879
genus

node742.members.0.js
3
596664
species

node743.members.0.js
95313
1
37569
superfamily

4
7128
family

4
82616
subfamily

4
469307
tribe

4
40103
genus

node748.members.0.js
4
40104
species

3
7117
family

1
82587
subfamily

1
180246
tribe

1
7126
genus

node753.members.0.js
1
7127
species

1
315994
subfamily

1
469310
genus

node756.members.0.js
1
469311
species

1
475335
subfamily

1
998835
genus

node759.members.0.js
1
998836
species

94513
7089
family

94513
475327
subfamily

1
475355
genus

node763.members.0.js
1
946274
species

node764.members.0.js
94413
38
7090
genus

node765.members.0.jsnode765.members.1.jsnode765.members.4.js
90613
7091
species

2
37568
superfamily

2
7139
family

2
65022
subfamily

2
81689
tribe

2
85584
genus

node771.members.0.js
2
85585
species

1
40092
superfamily

1
40093
family

1
218718
subfamily

1
876080
genus

node776.members.0.js
1
876081
species

1
41015
superfamily

1
58822
family

1
431644
subfamily

1
431647
tribe

1
431655
genus

node782.members.0.js
1
1498834
species

node783.members.0.jsnode783.members.4.js
92351827
2958
7399
order

node784.members.0.jsnode784.members.1.js
86771718
691
7434
suborder

node785.members.0.js
85761518
1
34725
superfamily

node786.members.0.jsnode786.members.1.jsnode786.members.4.js
85751518
3703210
36668
family

176614
43085
subfamily

176614
141711
tribe

176614
604375
genus

node790.members.0.jsnode790.members.1.jsnode790.members.4.js
176614
610380
species

69521
7479
subfamily

69521
72773
tribe

69521
13390
genus

node794.members.0.jsnode794.members.1.jsnode794.members.4.js
69521
104421
species

5771
219563
subfamily

5771
219768
tribe

5771
219769
genus

node798.members.0.jsnode798.members.1.js
5771
443821
species

node799.members.0.js
183493
96
34695
subfamily

135032
143999
tribe

135032
64782
genus

node802.members.0.jsnode802.members.1.jsnode802.members.4.js
135032
103372
species

38561
144017
tribe

38561
13685
genus

node805.members.0.jsnode805.members.1.jsnode805.members.4.js
38561
13686
species

3
144015
tribe

3
81628
genus

node808.members.0.js
3
88063
species

321
34735
superfamily

16
124286
family

16
156330
subfamily

16
156331
tribe

16
132116
genus

node814.members.0.js
16
143995
species

node815.members.0.js
161
3
7458
family

131
70987
subfamily

131
83321
tribe

node818.members.0.js
131
4
7459
genus

node819.members.0.jsnode819.members.1.js
41
7460
species

node820.members.0.js
1
7463
species

1
7461
species

node822.members.0.js
1
94128
subspecies

node823.members.0.js
3
7462
species

17011
7401
superfamily

node825.members.0.js
13211
10
7402
family

node826.members.0.js
1121
7
65207
subfamily

711
51538
genus

node828.members.0.jsnode828.members.4.js
711
69319
species

28
32390
genus

node830.members.0.js
28
51543
species

6
92993
genus

node832.members.0.js
6
463051
species

101
68883
subfamily

101
58738
genus

node835.members.0.jsnode835.members.1.js
101
64838
species

38
7408
family

38
65140
subfamily

38
29048
genus

node839.members.0.js
38
219576
species

93
7422
superfamily

93
7423
family

93
272242
subfamily

93
7424
genus

node844.members.0.js
93
7425
species

14555136
7041
order

node846.members.0.js
14545136
2
41084
suborder

3
41087
infraorder

2
71192
superfamily

2
50527
family

2
261156
subfamily

2
195164
genus

node852.members.0.js
2
224129
species

1
71193
superfamily

1
30009
family

1
195166
subfamily

1
1490192
genus

node857.members.0.js
1
1490193
species

1
41085
infraorder

1
75543
superfamily

1
29026
family

1
82877
subfamily

1
29027
genus

node863.members.0.js
1
29028
species

node864.members.0.js
14475136
37
41088
infraorder

3
71528
superfamily

3
27439
family

2
63710
subfamily

2
63711
tribe

2
226742
subtribe

2
27440
genus

node871.members.0.js
2
27441
species

1
63707
subfamily

1
63712
tribe

1
7538
genus

node875.members.0.js
1
7539
species

30
71529
superfamily

30
7042
family

30
55867
subfamily

30
77156
genus

node880.members.0.js
30
77166
species

13725136
71527
superfamily

7
34672
family

7
34673
genus

node884.members.0.js
7
34674
species

13655136
7065
family

13655136
7069
genus

node887.members.0.jsnode887.members.1.jsnode887.members.3.jsnode887.members.4.js
13655136
7070
species

5
71526
superfamily

2
196993
family

2
1169898
genus

node891.members.0.js
2
1169899
species

3
196984
family

3
57999
genus

node894.members.0.js
3
58000
species

1
41086
infraorder

1
75546
superfamily

1
7055
family

1
7062
subfamily

1
7063
genus

node900.members.0.js
1
7064
species

1
41071
suborder

1
535378
superfamily

node903.members.0.js
1
50515
family

node904.members.0.jsnode904.members.1.jsnode904.members.2.js
4139122
3211
7147
order

node905.members.0.js
270511
4
7148
suborder

3
43784
infraorder

3
41830
superfamily

3
33406
family

3
43793
subfamily

3
52722
tribe

3
39757
genus

node912.members.0.js
3
39758
species

node913.members.0.js
263511
22
43786
infraorder

10931
41827
superfamily

node915.members.0.jsnode915.members.1.js
10931
111
7157
family

30
43816
subfamily

node917.members.0.js
30
2
7164
genus

3
44543
subgenus

node919.members.0.js
3
7167
species

18
44534
subgenus

node921.members.0.js
13
7165
species

node922.members.0.js
5
30069
species

7
44482
subgenus

7
59131
species group

node925.members.0.js
7
74873
species

node926.members.0.jsnode926.members.1.jsnode926.members.4.js
6821
321
43817
subfamily

42
1056966
tribe

42
7158
genus

42
53541
subgenus

node930.members.0.js
42
7159
species

2
308729
tribe

2
174825
genus

node933.members.0.js
2
332058
species

21
53550
tribe

21
7174
genus

21
53527
subgenus

node937.members.0.js
1
38742
species

node938.members.0.js
5
7176
species

node939.members.0.js
15
7175
species

13221
41828
superfamily

13021
7149
family

13021
54970
subfamily

node943.members.0.js
13021
9
72530
tribe

1
36167
genus

node945.members.0.js
1
36168
species

node946.members.0.jsnode946.members.1.js
12021
641
7150
genus

511
41809
subgenus

node948.members.0.jsnode948.members.1.js
511
7153
species

node949.members.0.js
51
1
72537
subgenus

node950.members.0.js
1
113492
species

node951.members.3.js
1
113505
species

node952.members.0.js
1
33397
species

node953.members.0.js
1
7154
species

node954.members.0.js
1
27468
species

1
7190
family

1
43813
subfamily

1
44570
tribe

1
7191
genus

1
47384
subgenus

node960.members.0.js
1
7192
species

1
41819
family

1
43801
subfamily

1
58262
tribe

1
41820
genus

1
58269
subgenus

node966.members.0.js
1
469754
species

node967.members.0.js
111311
4
7203
suborder

5
43735
infraorder

5
1262365
superfamily

5
7205
family

5
43920
subfamily

5
59848
tribe

5
27458
genus

node974.members.0.js
5
27459
species

1
43734
infraorder

1
34687
family

1
343564
subfamily

1
343581
genus

node979.members.0.js
1
343691
species

node980.members.0.jsnode980.members.3.js
101311
171
43733
infraorder

7
43754
superfamily

7
7366
family

7
43910
subfamily

7
57894
tribe

7
7369
genus

7
44052
subgenus

node987.members.0.js
7
7370
species

4
43753
superfamily

4
7392
family

4
7393
genus

4
44051
subgenus

node992.members.0.js
4
7397
species

1
43755
superfamily

node994.members.0.js
1
7371
family

10
43752
superfamily

10
7211
family

10
164860
subfamily

7
43871
tribe

7
27456
genus

7
47833
subgenus

node1001.members.0.js
7
28588
species

3
164862
tribe

3
7212
genus

3
474492
subgenus

node1005.members.0.js
3
7213
species

5631
43746
superfamily

5631
7214
family

5631
43845
subfamily

5631
46877
tribe

5631
46879
subtribe

node1011.members.0.js
5431
2
7215
genus

node1012.members.0.js
4031
1
32341
subgenus

2
32355
species group

2
32358
species subgroup

node1015.members.0.js
2
1
7237
species

node1016.members.0.js
1
46245
subspecies

3611
32346
species group

node1018.members.0.js
3411
5
32351
species subgroup

node1019.members.0.jsnode1019.members.4.js
111
7245
species

node1020.members.0.jsnode1020.members.1.js
161
7227
species

node1021.members.0.js
1
7240
species

node1022.members.0.js
1
7226
species

2
32347
species subgroup

node1024.members.0.js
2
7217
species

12
32365
species group

12
32367
species subgroup

node1027.members.0.jsnode1027.members.1.js
12
7260
species

1
48302
species group

1
32378
species subgroup

node1030.members.0.js
1
7222
species

2
48300
species group

2
32381
species subgroup

node1033.members.0.js
2
32382
species

9
32281
subgenus

1
32321
species group

1
32324
species subgroup

node1037.members.0.js
1
7230
species

8
32335
species group

node1039.members.0.js
8
7244
species

2
7292
genus

2
44003
species group

node1042.members.0.js
2
7293
species

6
43745
superfamily

node1044.members.0.js
6
4
139644
family

2
139679
genus

node1046.members.0.js
1
139649
species

node1047.members.0.js
1
139650
species

node1048.members.0.js
19725120
6
33342
infraclass

36018
85819
order

36018
30005
suborder

36018
121221
family

36018
121222
genus

36018
121225
species

node1054.members.0.jsnode1054.members.4.js
36018
121224
subspecies

501
30259
order

501
160148
suborder

501
160149
family

node1058.members.0.jsnode1058.members.3.js
501
301
160150
genus

node1059.members.0.js
2
550478
species

node1060.members.0.js
3
209927
species

node1061.members.0.js
1
209926
species

node1062.members.0.js
14
185214
species

node1063.members.0.js
155652
28
7524
order

7612
33375
superfamily

7612
30092
family

7612
121844
genus

node1067.members.0.jsnode1067.members.1.js
7612
121845
species

74332
33385
superfamily

74332
27482
family

node1070.members.0.js
74332
4
133076
subfamily

73732
33386
tribe

73732
7028
genus

node1073.members.0.jsnode1073.members.1.jsnode1073.members.4.js
73732
7029
species

2
33387
tribe

2
80764
genus

2
464929
subgenus

node1077.members.0.js
2
80765
species

18
33355
superfamily

18
57993
family

18
528086
subfamily

18
58007
genus

node1082.members.0.js
18
58008
species

1
33377
superfamily

1
7036
family

1
33379
subfamily

1
7037
genus

node1087.members.0.js
1
7038
species

3
50616
family

3
280212
subfamily

3
50617
genus

node1091.members.0.js
3
219578
species

1
36151
superfamily

1
33362
family

1
130551
subfamily

1
108930
genus

node1096.members.0.js
1
108931
species

1
33366
superfamily

1
30086
family

1
130642
genus

node1100.members.0.js
1
798355
species

401
6657
subphylum

20
6681
class

node1103.members.0.js
20
3
72041
subclass

17
6682
superorder

17
6683
order

17
6692
suborder

node1107.members.0.js
17
10
6752
infraorder

1
6774
superfamily

1
6757
family

1
80835
genus

node1111.members.0.js
1
80836
species

2
29962
superfamily

2
72876
family

2
95601
genus

node1115.members.0.js
2
95602
species

3
652086
superfamily

3
483405
family

3
483406
genus

node1119.members.0.js
3
483407
species

1
6778
superfamily

1
108326
family

1
156091
genus

node1123.members.0.js
1
483417
species

191
6658
class

191
116557
subclass

191
84337
order

191
6665
suborder

191
116561
infraorder

191
77658
family

191
6668
genus

node1131.members.0.jsnode1131.members.2.js
191
6669
species

1
72037
class

1
6830
subclass

1
116569
infraclass

1
116571
superorder

1
72033
order

1
72034
family

1
72035
genus

node1139.members.0.js
1
72036
species

2988843531494
6843
subphylum

node1141.members.0.jsnode1141.members.1.jsnode1141.members.4.js
2988843531494
22143
6854
class

521353142
6933
subclass

node1143.members.0.js
511353142
1
6934
superorder

141353142
6935
order

141353142
297308
superfamily

12353142
6939
family

11353142
426442
subfamily

11353142
6944
genus

node1149.members.0.jsnode1149.members.2.jsnode1149.members.3.jsnode1149.members.4.js
11353142
6945
species

1
426441
subfamily

1
6942
genus

node1152.members.0.js
1
6943
species

21
297309
family

21
1029658
genus

node1155.members.0.jsnode1155.members.1.js
21
1029659
species

36
34634
order

36
281668
suborder

36
1723665
infraorder

36
1253825
superfamily

36
34636
family

36
425253
subfamily

36
34637
genus

node1163.members.0.js
36
34638
species

1
6946
superorder

1
6947
suborder

1
83146
superfamily

node1167.members.0.js
1
32262
family

node1168.members.0.js
29128249
3
6893
order

node1169.members.4.js
29028249
32
6905
suborder

1
74972
superfamily

1
74973
family

1
93698
genus

node1173.members.0.js
1
434756
species

2900828
175332
superfamily

2900828
175333
family

2900828
175340
genus

node1177.members.0.jsnode1177.members.1.jsnode1177.members.4.js
2900828
407821
species

19
74975
superfamily

9
34643
family

9
449632
genus

node1181.members.4.js
9
114398
species

node1182.members.0.js
1
6913
family

7
6894
suborder

6
196121
family

6
196122
genus

node1186.members.0.js
5
196123
species

node1187.members.0.js
1
196124
species

1
6895
family

1
6896
genus

node1190.members.0.js
1
64965
species

2
6855
order

1
70336
superfamily

1
6856
family

1
6875
genus

node1195.members.0.js
1
6879
species

1
70337
superfamily

1
100977
family

1
259485
subfamily

1
259492
tribe

1
259495
subtribe

1
259499
genus

node1202.members.0.js
1
311986
species

32
7586
phylum

32
7624
superclass

32
7625
class

32
7638
subclass

32
7674
superorder

32
7675
order

32
31181
family

32
7664
genus

node1211.members.0.js
32
7668
species

node1212.members.0.js
2422
2
6157
phylum

5
6163
order

5
166333
suborder

5
166363
superfamily

5
31256
family

5
166366
subfamily

5
31257
genus

node1219.members.0.js
5
46768
species

112
6178
class

112
6179
subclass

9
6180
order

9
31244
superfamily

9
31245
family

9
6181
genus

node1226.members.0.js
6
6185
species

node1227.members.0.js
3
6183
species

22
6193
order

22
6194
suborder

22
6196
family

1
6197
genus

node1232.members.0.js
1
6198
species

12
79922
genus

node1234.members.0.jsnode1234.members.2.js
12
79923
species

5
6159
order

5
1292243
suborder

5
1292253
superfamily

5
31262
family

5
52316
genus

node1240.members.0.js
5
6162
species

12
6199
class

12
6200
subclass

12
6201
order

2
6214
family

2
6215
genus

node1246.members.1.js
2
85433
species

1
6208
family

1
6209
genus

node1249.members.0.js
1
6210
species

2
137627
subclass

2
5789
order

2
1115744
family

2
5790
genus

node1254.members.0.js
2
5791
species

node1255.members.0.js
8
5
33083
order

node1256.members.0.js
3
1
5782
genus

node1257.members.0.js
1
5786
species

node1258.members.0.js
1
261658
species

node1259.members.0.jsnode1259.members.1.jsnode1259.members.2.js
2287581765657319
305386
4751
kingdom

2
4761
phylum

2
451435
class

2
451442
order

2
100474
genus

node1264.members.0.js
2
109871
species

node1265.members.0.js
1
1603295
species

4885769406916
214504
phylum

4885769406916
214506
class

4885769406916
36750
order

4885769406916
36751
family

4885769406916
1129544
genus

node1271.members.0.jsnode1271.members.1.jsnode1271.members.2.jsnode1271.members.3.jsnode1271.members.4.js
4885769406916
588596
species

345
451507
subphylum

node1273.members.0.js
345
1
4827
order

245
1344963
suborder

2
1344955
family

node1276.members.0.js
2
1
4842
genus

node1277.members.0.js
1
936053
species

45
34489
family

45
4830
genus

node1280.members.2.js
45
91626
species

node1281.members.0.js
81
1
6029
phylum

7
6032
suborder

4
27974
family

node1284.members.0.js
4
1
27977
genus

node1285.members.0.js
2
27978
species

node1286.members.0.js
1
35231
species

1
307623
family

1
311453
genus

node1289.members.0.js
1
723287
species

2
36734
family

node1291.members.0.js
2
6033
genus

1
1633384
genus

node1293.members.2.js
1
1485682
species

node1294.members.0.jsnode1294.members.1.jsnode1294.members.2.js
148045639343
31147
451864
subkingdom

node1295.members.0.jsnode1295.members.2.js
138141577823
207
4890
phylum

node1296.members.0.jsnode1296.members.1.jsnode1296.members.2.jsnode1296.members.4.js
135937576423
905107302
147538
subphylum

1
189478
class

1
189479
order

node1299.members.0.js
1
47021
family

node1300.members.0.jsnode1300.members.1.jsnode1300.members.2.js
174954
3011
147545
class

node1301.members.0.js
10048
8
451870
subclass

node1302.members.0.js
8848
20
34395
order

node1303.members.0.jsnode1303.members.1.js
5128
252
43219
family

31
5583
genus

node1305.members.2.js
1
1033840
species

node1306.members.0.js
2
91928
species

node1307.members.0.js
1
212818
species

205
61458
genus

node1309.members.0.jsnode1309.members.2.js
205
61459
species

1
5587
genus

node1311.members.0.js
1
86056
species

1
82105
genus

node1313.members.0.js
1
569365
species

12
43220
genus

node1315.members.0.js
1
43229
species

node1316.members.2.js
2
43228
species

172
1341112
family

172
226991
genus

node1319.members.0.jsnode1319.members.1.js
172
293227
species

4
146291
order

4
146292
family

4
364710
genus

node1323.members.0.js
4
364733
species

node1324.members.0.js
44445
9
451871
subclass

node1325.members.0.jsnode1325.members.2.js
28444
216
5042
order

21
28568
family

21
5094
genus

node1328.members.0.jsnode1328.members.2.js
21
28564
species

node1329.members.0.jsnode1329.members.1.jsnode1329.members.2.js
21427
418
1131492
family

node1330.members.0.jsnode1330.members.1.jsnode1330.members.2.js
6314
334
5073
genus

node1331.members.0.jsnode1331.members.2.js
310
1108849
species

node1332.members.0.jsnode1332.members.2.js
115
31
5052
genus

node1333.members.2.js
1
746128
species

node1334.members.2.js
1
33178
species

node1335.members.2.js
1
5057
species

node1336.members.0.js
7
5059
species

node1337.members.0.jsnode1337.members.2.js
11
396024
species

3
1131624
family

3
5092
genus

node1340.members.0.js
3
264951
species

71
33183
order

node1342.members.0.js
5
1
299071
family

4
5036
genus

node1344.members.0.js
4
5037
species

1
38946
genus

node1346.members.2.js
1
1048829
species

2
5500
genus

node1348.members.0.js
2
5501
species

node1349.members.0.jsnode1349.members.2.js
18120
53
147548
class

68
34379
family

68
78156
genus

node1352.members.0.jsnode1352.members.2.js
34
1420915
species

node1353.members.2.js
1
1420913
species

node1354.members.2.js
2
1420901
species

node1355.members.0.js
1
1420914
species

node1356.members.0.jsnode1356.members.2.js
21
79858
species

24
37240
family

24
78141
genus

node1359.members.0.jsnode1359.members.2.js
24
78148
species

node1360.members.0.jsnode1360.members.1.jsnode1360.members.2.js
415
213
5178
order

1
5181
family

1
101851
genus

node1363.members.2.js
1
101852
species

node1364.members.0.js
11
1
28983
family

1
5179
genus

node1366.members.2.js
1
5180
species

1
76657
family

1
324777
genus

node1369.members.0.js
1
698440
species

1
5120
order

1
34371
family

1
5121
genus

node1373.members.0.js
1
52586
species

node1374.members.0.jsnode1374.members.1.jsnode1374.members.2.js
19114493621
39189
147541
class

826483921
451868
subclass

node1376.members.0.jsnode1376.members.2.js
826483921
17
92860
order

node1377.members.0.jsnode1377.members.1.jsnode1377.members.2.jsnode1377.members.4.js
816483221
51432121
715340
suborder

510631
5020
family

510631
1351751
genus

node1380.members.0.jsnode1380.members.2.jsnode1380.members.3.js
510631
13684
species

node1381.members.0.jsnode1381.members.1.jsnode1381.members.2.js
2014701
141218
28556
family

node1382.members.0.jsnode1382.members.2.js
2144
251
5027
genus

node1383.members.2.js
33
45151
species

60
53485
species

node1385.members.2.js
60
97479
forma

124
39952
genus

node1387.members.0.jsnode1387.members.2.js
124
93612
species

node1388.members.0.jsnode1388.members.2.jsnode1388.members.3.js
3841
3561
33194
genus

node1389.members.2.js
2
5016
species

node1390.members.2.js
6
45130
species

node1391.members.2.js
6
101162
species

node1392.members.2.js
14
5017
species

5187
34374
family

5187
5021
genus

node1395.members.0.jsnode1395.members.1.jsnode1395.members.2.js
5187
5022
species

node1396.members.0.js
4755
3
451867
subclass

311
5014
order

311
1570301
family

node1399.members.0.jsnode1399.members.2.js
311
191
5579
genus

node1400.members.0.js
2
1042127
species

node1401.members.0.js
2
5580
species

node1402.members.0.js
4
559561
species

node1403.members.0.js
4
46634
species

node1404.members.0.js
1354
6
134362
order

2
668547
family

2
483074
genus

node1407.members.0.js
2
1709381
species

node1408.members.0.jsnode1408.members.1.js
554
31
93133
family

1
237179
genus

node1410.members.1.js
1
85929
species

21
242508
genus

node1412.members.0.jsnode1412.members.1.js
21
64363
species

23
131324
genus

node1414.members.1.jsnode1414.members.2.js
23
83344
species

1
131316
genus

node1416.members.2.js
1
5499
species

192
451869
order

node1418.members.0.js
192
8
45131
family

62
35724
genus

node1420.members.0.jsnode1420.members.2.js
62
35725
species

5
407951
genus

node1422.members.0.js
5
310453
species

421
1111111
order

421
1111112
family

421
1494215
genus

node1426.members.0.jsnode1426.members.1.jsnode1426.members.2.js
421
253628
species

1
147547
class

1
388435
subclass

1
5197
order

1
157822
suborder

1
5198
family

1
5199
genus

node1433.members.0.js
1
27339
species

node1434.members.0.jsnode1434.members.2.js
69324
32
147550
class

node1435.members.0.jsnode1435.members.2.js
59314
21
222543
subclass

node1436.members.0.jsnode1436.members.1.js
5428
32
5125
order

1
485699
tribe

1
124426
genus

node1439.members.0.js
1
1159556
species

355
110618
family

node1441.members.0.jsnode1441.members.2.js
355
312
5506
genus

node1442.members.0.jsnode1442.members.2.js
22
5507
species

node1443.members.0.js
1
117187
species

node1444.members.0.jsnode1444.members.2.js
11
40199
species

node1445.members.0.js
31
1
474943
family

1
5581
genus

node1447.members.2.js
1
176275
species

2
45234
genus

node1449.members.0.js
2
99897
species

2
1667166
family

node1451.members.0.js
2
1
74721
genus

node1452.members.0.js
1
74722
species

1
5129
family

1
5543
genus

node1455.members.0.js
1
63577
species

node1456.members.0.js
92
2
34397
family

1
475080
genus

node1458.members.2.js
1
1531966
species

1
5110
genus

node1460.members.2.js
1
5111
species

7
5529
genus

node1462.members.0.js
7
1650735
species

11
5592
order

11
5593
family

11
41687
genus

node1466.members.0.jsnode1466.members.1.js
11
563466
species

25
1028384
order

1
1033978
family

node1469.members.0.js
1
1036719
genus

15
681950
family

node1471.members.2.js
15
3
5455
genus

node1472.members.0.jsnode1472.members.2.js
12
80884
species

node1473.members.0.jsnode1473.members.2.js
63
12
222544
subclass

4
5139
order

2
35718
family

1
49009
genus

node1477.members.0.js
1
78579
species

1
35719
genus

node1479.members.0.js
1
35720
species

2
5148
family

2
5146
genus

node1482.members.0.js
2
5147
species

11
5151
order

11
5152
family

1
5159
genus

node1486.members.0.js
1
61273
species

1
29907
genus

node1488.members.2.js
1
29908
species

15
222545
subclass

node1490.members.2.js
15
4
37989
order

11
54958
family

11
37840
genus

node1493.members.0.jsnode1493.members.2.js
11
393283
species

247
147537
subphylum

247
4891
class

node1496.members.0.js
247
1
4892
order

17
766764
family

node1498.members.2.js
7
2
1535326
genus

node1499.members.2.js
1
5480
species

node1500.members.2.js
4
5482
species

1
766728
genus

node1502.members.0.js
1
4929
species

node1503.members.1.js
4
4893
family

node1504.members.0.jsnode1504.members.2.js
6835682
38
5204
phylum

node1505.members.0.jsnode1505.members.2.js
1125382
115
452284
subphylum

392
5257
class

392
5267
order

node1508.members.0.jsnode1508.members.2.js
392
390
5268
family

1
63265
genus

node1510.members.2.js
1
72558
species

1
5269
genus

node1512.members.2.js
1
120017
species

724312
1538075
class

724312
162474
order

724312
742845
family

node1516.members.0.jsnode1516.members.2.js
724312
1111
55193
genus

node1517.members.0.jsnode1517.members.1.jsnode1517.members.2.jsnode1517.members.3.js
623022
76773
species

node1518.members.2.js
18
76777
species

node1519.members.1.js
211
1
29000
subphylum

node1520.members.2.js
1
162481
class

1
432025
class

1
432026
order

1
165795
family

1
34348
genus

node1525.members.0.js
1
34349
species

1
162484
class

1
5258
order

1
5259
family

1
5260
genus

node1530.members.0.js
1
203908
species

5221
5302
subphylum

1
155616
class

1
5234
order

1
5215
family

1
105767
genus

node1536.members.0.js
1
5217
species

node1537.members.0.jsnode1537.members.2.js
5121
37
155619
class

2
139380
order

2
40424
family

2
167346
genus

node1541.members.0.js
2
208960
species

40
452342
order

39
103376
family

39
5644
genus

node1545.members.0.js
39
40492
species

1
40420
family

1
13562
genus

node1548.members.0.js
1
984962
species

41
36064
order

41
5250
family

41
1322061
genus

node1552.members.0.jsnode1552.members.2.js
41
456999
species

1
452339
order

1
452340
family

1
40443
genus

node1556.members.0.js
1
104355
species

11
297313
order

1
65702
genus

node1559.members.0.js
1
65672
species

1
358905
genus

node1561.members.2.js
1
109899
species

node1562.members.2.js
12
6
452333
subclass

node1563.members.2.js
2
1
68889
order

1
227327
suborder

1
227329
family

1
37467
genus

node1567.members.2.js
1
37468
species

4
5338
order

4
654128
family

node1570.members.2.js
4
1
221102
genus

node1571.members.2.js
3
221103
species

4214
5794
phylum

1
35086
class

1
35087
order

1
35088
family

1
35089
genus

node1577.members.1.js
1
110365
species

124
422676
class

124
5819
order

124
1639119
family

124
5820
genus

10
418101
subgenus

1
5825
species

node1584.members.0.js
1
31271
subspecies

node1585.members.0.js
9
5821
species

22
418103
subgenus

node1587.members.2.js
2
5855
species

node1588.members.0.js
2
52288
species

2
418107
subgenus

node1590.members.2.js
2
5833
species

30
5796
class

30
75739
order

30
423054
suborder

30
5799
family

node1595.members.0.js
30
4
5800
genus

node1596.members.0.js
24
51316
species

node1597.members.0.js
2
51314
species

node1598.members.0.js
2494212584623152
4
33090
kingdom

node1599.members.0.jsnode1599.members.1.jsnode1599.members.2.jsnode1599.members.3.jsnode1599.members.4.js
2344212584623152
431440123124
35493
phylum

155
261009
order

155
22097
family

155
13332
genus

node1603.members.2.js
155
13333
species

5275385322
71274
subclass

1
4209
order

node1606.members.0.js
1
4210
family

2275377322
4143
order

2275377322
4196
family

2275377322
192258
genus

node1610.members.0.jsnode1610.members.1.jsnode1610.members.2.jsnode1610.members.3.jsnode1610.members.4.js
2275377322
192259
species

28
4069
order

node1612.members.0.js
28
1
4070
family

2
424551
subfamily

2
424574
tribe

2
4107
genus

node1616.members.2.js
2
50514
species

16
424554
subfamily

16
424562
tribe

16
4085
genus

node1620.members.2.js
6
4097
species

node1621.members.0.js
1
4096
species

411
131220
class

411
3172
order

411
3173
family

411
3174
genus

node1626.members.0.jsnode1626.members.4.js
411
3175
species

2
3524
order

2
3563
family

2
3554
genus

2
161934
species

node1631.members.0.js
2
3555
subspecies

137613
232378
order

137613
4429
family

137613
4430
genus

node1635.members.0.jsnode1635.members.1.jsnode1635.members.2.jsnode1635.members.3.js
137613
4432
species

9196111
3313
subclass

9196111
1446380
order

node1638.members.0.js
9196111
4
3318
family

2196111
3337
genus

node1640.members.0.js
2
1
139271
subgenus

node1641.members.0.js
1
3347
species

196111
139272
subgenus

node1643.members.2.jsnode1643.members.3.jsnode1643.members.4.js
196111
88728
species

3
3328
genus

node1645.members.0.js
2
3329
species

node1646.members.0.js
1
3332
species

node1647.members.0.jsnode1647.members.2.jsnode1647.members.4.js
119314321124
142711
71275
subclass

152
3744
order

1
3745
family

1
171637
subfamily

1
3754
genus

node1652.members.0.js
1
3760
species

52
3487
family

52
3497
genus

node1655.members.2.js
52
981085
species

775
3646
order

104
3688
family

104
238069
tribe

node1659.members.0.js
104
1
3689
genus

node1660.members.0.jsnode1660.members.2.js
94
75702
species

671
3977
family

67
235629
subfamily

67
235880
tribe

67
3987
genus

node1665.members.0.js
67
3988
species

1
235631
subfamily

1
235887
tribe

1
3995
genus

node1669.members.2.js
1
180498
species

612762
72025
order

612762
3803
family

node1672.members.0.js
612762
6
3814
subfamily

67
163742
tribe

67
3877
genus

node1675.members.2.js
67
3880
species

12092
163725
tribe

12092
3817
genus

node1678.members.2.jsnode1678.members.3.js
12092
170720
species

7312715101
3699
order

1312715101
3700
family

1312715101
981071
tribe

1312715101
3705
genus

node1683.members.0.jsnode1683.members.1.jsnode1683.members.2.jsnode1683.members.3.jsnode1683.members.4.js
1312715101
3708
species

6
301454
family

6
1168313
genus

node1686.members.0.js
6
28532
species

422
403667
order

422
3602
family

422
3603
genus

node1690.members.0.jsnode1690.members.2.jsnode1690.members.4.js
422
29760
species

6
41944
order

6
3931
family

6
1699524
tribe

6
3932
genus

node1695.members.0.js
6
71139
species

1
41938
order

1
3629
family

1
214909
subfamily

1
3640
genus

node1700.members.0.js
1
3641
species

3
3502
order

3
3503
family

3
21019
genus

node1704.members.0.js
3
60419
species

node1705.members.2.js
71111
1
4447
class

71101
4734
subclass

1
40551
order

1
4710
family

1
169697
subfamily

1
169705
tribe

1
169729
subtribe

1
51952
genus

node1713.members.2.js
1
51953
species

7191
38820
order

node1715.members.2.js
7191
4
4479
family

31
147369
subfamily

31
147429
tribe

31
1648029
subtribe

31
4575
genus

node1720.members.0.js
31
3
4577
species

node1721.members.3.js
1
4579
subspecies

3
147367
subfamily

3
147380
tribe

3
1648021
subtribe

3
4527
genus

node1726.members.0.js
3
4530
species

115
147368
subfamily

node1728.members.1.js
115
1
147389
tribe

1
1648017
subtribe

1
4549
genus

node1731.members.0.js
1
4550
species

5
1648030
subtribe

4
4564
genus

node1734.members.2.js
4
4565
species

1
4480
genus

node1736.members.2.js
1
37682
species

7
3214
class

7
114656
subclass

7
3215
order

7
3216
family

7
3217
genus

node1742.members.0.js
7
3218
species

node1743.members.0.js
11
4
3041
phylum

node1744.members.0.js
7
3
75966
class

4
35472
family

4
41891
genus

node1747.members.0.js
4
248742
species

11
5653
order

node1749.members.0.js
11
1
5654
family

1
5690
genus

1
47569
subgenus

node1752.members.2.js
1
5692
species

1
4762
class

1
4776
order

1
4783
genus

node1756.members.0.js
1
4792
species

node1757.members.0.js
22237
1
431838
subphylum

21237
33829
class

21237
194286
subclass

21237
693921
order

node1761.members.0.jsnode1761.members.2.js
21237
41
57506
family

15236
1001748
subfamily

15236
5948
genus

node1764.members.0.jsnode1764.members.1.jsnode1764.members.2.js
15236
5949
species

2
1001750
subfamily

2
5943
genus

node1767.members.0.js
2
1172189
species

84
28009
order

2
81529
family

2
86017
genus

node1771.members.0.js
2
946362
species

82
81524
family

82
81525
genus

node1774.members.0.js
82
81824
species

911
1485168
order

911
5754
genus

node1777.members.0.jsnode1777.members.4.js
911
5755
species

1
5739
family

1
68459
subfamily

1
5740
genus

node1781.members.0.js
1
5741
species

1
5752
class

1
41165
order

1
5765
family

1
5761
genus

node1786.members.3.js
1
5762
species

node1787.members.0.jsnode1787.members.1.jsnode1787.members.2.js
16236
32630
species

node1788.members.0.jsnode1788.members.1.jsnode1788.members.2.jsnode1788.members.3.jsnode1788.members.4.js
11058812796082847045166532301971
308729777237595573574
2
superkingdom

node1789.members.0.jsnode1789.members.1.jsnode1789.members.2.jsnode1789.members.4.js
47503511084
3235292162
1117
phylum

226
1212
order

226
1213
family

226
1214
genus

node1793.members.0.js
226
1216
species

node1794.members.0.jsnode1794.members.1.js
235
31
1189
order

2
29417
genus

node1796.members.0.js
2
83541
species

22
1123
genus

node1798.members.0.jsnode1798.members.1.js
22
1124
species

6
221282
genus

node1800.members.0.js
6
221287
species

node1801.members.0.js
92
2
1190
genus

node1802.members.0.jsnode1802.members.1.js
21
1173024
species

node1803.members.0.js
2
1173023
species

node1804.members.0.jsnode1804.members.1.js
21
1174528
species

node1805.members.0.js
1
92938
species

1
996924
genus

node1807.members.0.js
1
996925
species

node1808.members.0.js
17012
1
52604
order

15712
54298
genus

node1810.members.0.jsnode1810.members.1.jsnode1810.members.4.js
15712
54299
species

1
102115
genus

node1812.members.0.js
1
102116
species

4
52607
genus

node1814.members.0.js
4
102125
species

7
44474
genus

node1816.members.0.js
7
54308
species

node1817.members.0.jsnode1817.members.1.js
379372
1027
1161
order

node1818.members.0.js
2421
10
1162
family

1
1175
genus

node1820.members.2.js
1
1176
species

11
159191
genus

node1822.members.0.jsnode1822.members.1.js
11
70799
species

2
1163
genus

node1824.members.0.js
1
163908
species

node1825.members.0.js
1
1172
species

3
56106
genus

node1827.members.0.js
3
142864
species

node1828.members.0.jsnode1828.members.1.js
81
31
1177
genus

node1829.members.0.js
1
28072
species

node1830.members.0.js
4
272131
species

node1831.members.0.js
12271
4
119859
family

3
188910
genus

node1833.members.0.js
3
643473
species

351
482629
genus

node1835.members.0.jsnode1835.members.2.js
351
482630
species

node1836.members.0.js
807
1
111782
genus

node1837.members.0.jsnode1837.members.1.js
613
379535
species

node1838.members.0.js
2
1246981
species

node1839.members.0.jsnode1839.members.1.js
164
1136105
species

963
1182
family

node1841.members.0.jsnode1841.members.1.js
963
21
1203
genus

node1842.members.0.js
9
34078
species

node1843.members.0.js
22
1233230
species

node1844.members.0.jsnode1844.members.1.js
632
1245922
species

3518
1185
family

11
373984
genus

node1847.members.0.jsnode1847.members.1.js
11
373994
species

node1848.members.0.js
3417
2
1186
genus

node1849.members.0.js
1
99598
species

node1850.members.0.jsnode1850.members.1.js
2816
32057
species

node1851.members.0.jsnode1851.members.1.js
31
32054
species

node1852.members.0.jsnode1852.members.1.jsnode1852.members.2.js
7171690
7443
1301283
subclass

node1853.members.0.jsnode1853.members.1.js
134317
371
1150
order

1
1205
genus

node1855.members.0.js
1
1206
species

35
54304
genus

node1857.members.0.js
2
54307
species

node1858.members.0.jsnode1858.members.2.js
15
1160
species

node1859.members.0.js
4411
3
47251
genus

node1860.members.0.js
34
1184
species

node1861.members.2.js
11
1173264
species

node1862.members.0.js
2
1229172
species

node1863.members.0.js
4
1487953
species

node1864.members.0.js
1
1385935
species

node1865.members.0.js
2
864702
species

1
1152
genus

node1867.members.0.js
1
82654
species

3
241421
genus

node1869.members.0.js
3
241425
species

node1870.members.0.js
8
1501433
genus

3
63132
genus

node1872.members.0.js
2
1173025
species

node1873.members.0.js
1
102127
species

81
1158
genus

node1875.members.0.jsnode1875.members.1.js
81
1173028
species

2
28073
genus

node1877.members.0.js
1
313612
species

node1878.members.0.js
1
118322
species

161
44471
genus

node1880.members.0.js
1
119532
species

node1881.members.0.jsnode1881.members.2.js
151
1173027
species

61
669368
genus

node1883.members.0.jsnode1883.members.1.js
61
64178
species

node1884.members.0.jsnode1884.members.1.js
509970
2522
1118
order

11
217161
genus

node1886.members.0.jsnode1886.members.1.js
11
1173032
species

4
582491
genus

node1888.members.0.js
4
582514
species

node1889.members.0.js
5
1
1125
genus

node1890.members.0.js
4
1160279
species

node1891.members.0.js
1070
2
43988
genus

node1892.members.0.js
3
395961
species

node1893.members.0.js
3
497965
species

node1894.members.0.jsnode1894.members.2.js
170
391612
species

node1895.members.0.js
1
43989
species

node1896.members.0.js
1243
11
1142
genus

node1897.members.0.jsnode1897.members.1.js
1133
927677
species

3
155977
genus

node1899.members.0.js
3
310037
species

1062
102231
genus

node1901.members.0.jsnode1901.members.1.js
1062
1173026
species

1
102234
genus

node1903.members.1.js
1
102235
species

3
263510
genus

node1905.members.0.js
3
263511
species

1
1129
genus

node1907.members.0.js
1
91464
species

node1908.members.2.js
5
1
1052815
phylum

4
1462351
genus

node1910.members.2.js
4
1462352
species

node1911.members.2.js
9
1256570
species

2810
32066
phylum

2810
203490
class

2810
203491
order

1810
203492
family

node1916.members.0.jsnode1916.members.2.js
1810
11
848
genus

node1917.members.2.js
809
1
851
species

node1918.members.2.js
808
155615
subspecies

1
1129771
family

1
32067
genus

node1921.members.0.js
1
157688
species

node1922.members.2.js
1
1120746
species

1
142182
phylum

1
219685
class

1
219686
order

1
219687
family

1
1706036
genus

node1928.members.0.js
1
861299
species

20631
51290
superphylum

10421
204428
phylum

10421
204429
class

node1932.members.0.jsnode1932.members.1.js
10421
24
51291
order

71
92713
family

node1934.members.2.js
1
112987
genus

7
282132
genus

node1936.members.0.js
7
362787
species

11
809
family

11
810
genus

node1939.members.2.js
1
83554
species

node1940.members.0.js
1
1444711
species

1
92712
family

1
34093
genus

node1943.members.3.js
1
83561
species

node1944.members.0.js
1021
1
74201
phylum

12
414999
class

12
415000
order

node1947.members.0.jsnode1947.members.1.js
12
11
134623
family

1
1148783
genus

node1949.members.1.js
1
1148786
species

node1950.members.0.js
1
1009415
species

1
134549
class

1
295577
genus

node1953.members.0.js
1
191863
species

61
203494
class

61
48461
order

node1956.members.2.js
1
382464
species

node1957.members.0.js
2
1
203557
family

1
2735
genus

node1959.members.0.js
1
1403819
species

4
1647988
family

4
239934
genus

node1962.members.0.js
1
239935
species

node1963.members.0.js
3
1263034
species

node1964.members.0.jsnode1964.members.2.js
22
1499967
species

node1965.members.0.jsnode1965.members.1.jsnode1965.members.2.jsnode1965.members.3.jsnode1965.members.4.js
7406712713541866298158282111204
13451738307122336144069
1224
phylum

5224513961
68525
subphylum

40921
29547
class

40921
213849
order

111
72293
family

1
209
genus

node1971.members.1.js
1
210
species

11
843
genus

node1973.members.0.jsnode1973.members.2.js
11
844
species

4081
72294
family

node1975.members.0.js
4081
133
194
genus

node1976.members.0.js
1
195
species

node1977.members.1.js
1
1031542
species

node1978.members.0.js
274
234
197
species

node1979.members.0.js
40
32022
subspecies

node1980.members.0.jsnode1980.members.2.js
1134313951
15
28221
class

21
69541
order

21
213421
family

21
890
genus

node1984.members.0.js
21
891
species

node1985.members.0.js
5
1268301
species

23
29
order

12
80812
suborder

12
49
family

12
39643
genus

node1990.members.0.jsnode1990.members.2.js
12
56
species

11
80811
suborder

11
39
family

1
224455
genus

node1994.members.0.js
1
394096
species

1
40
genus

node1996.members.2.js
1
41
species

31
1551504
genus

node1998.members.0.jsnode1998.members.1.js
31
673862
species

1113
213115
order

node2000.members.2.js
1113
1
194924
family

node2001.members.2.js
1112
6
872
genus

node2002.members.2.js
1104
184917
species

node2003.members.2.js
2
878
species

node2004.members.2.js
6274
1
213118
order

3
213121
family

3
893
genus

node2007.members.1.js
3
53332
species

3273
213119
family

1
115780
genus

node2010.members.1.js
1
115781
species

233
1549126
genus

node2012.members.2.js
233
90728
species

11
896
genus

node2014.members.1.jsnode2014.members.2.js
11
181663
species

39
2295
genus

node2016.members.2.js
39
201089
species

1
153027
genus

node2018.members.1.js
1
153026
species

80
213481
order

28
263369
family

node2021.members.0.js
28
27
146784
genus

node2022.members.0.js
1
1353529
species

48
1652132
family

48
1652133
genus

node2025.members.0.js
48
97084
species

4
213483
family

node2027.members.0.js
4
2
958
genus

node2028.members.0.js
1
1569284
species

node2029.members.0.js
1
959
species

1361
213113
order

1361
117942
family

1361
33001
genus

node2033.members.0.jsnode2033.members.1.jsnode2033.members.3.js
1361
33002
species

node2034.members.0.jsnode2034.members.1.jsnode2034.members.2.jsnode2034.members.3.jsnode2034.members.4.js
811112336760520583372903
491828035066661104829
28211
class

node2035.members.2.js
1
1282876
species

node2036.members.0.jsnode2036.members.1.jsnode2036.members.2.js
50431587728533
111868
204441
order

node2037.members.0.jsnode2037.members.1.jsnode2037.members.2.jsnode2037.members.4.js
41106892
20141
433
family

243
441
genus

node2039.members.1.js
1
479935
species

node2040.members.1.jsnode2040.members.2.js
143
38308
species

node2041.members.2.js
19261
78
434
genus

node2042.members.0.jsnode2042.members.2.js
1815
65959
species

node2043.members.0.js
1
438
species

node2044.members.2.js
168
104102
species

1
1434011
genus

node2046.members.1.js
1
33995
species

node2047.members.2.js
68
19
89583
genus

node2048.members.2.js
26
1004836
species

node2049.members.2.js
23
33996
species

node2050.members.2.js
1
365532
genus

1
364409
genus

node2052.members.0.js
1
364410
species

63121
91914
genus

node2054.members.1.jsnode2054.members.2.jsnode2054.members.4.js
63121
610243
species

1
522
genus

node2056.members.0.js
1
62140
species

node2057.members.1.jsnode2057.members.2.js
113
710788
species

4
445219
genus

node2059.members.0.js
4
445220
species

node2060.members.0.jsnode2060.members.1.jsnode2060.members.2.jsnode2060.members.3.jsnode2060.members.4.js
4311562028531
24713754013
41295
family

node2061.members.2.js
5738443514
2
191
genus

node2062.members.2.js
18
193
species

node2063.members.1.jsnode2063.members.2.jsnode2063.members.3.jsnode2063.members.4.js
5738023514
1262706
species

node2064.members.2.js
22
192
species

11
414051
genus

node2066.members.0.jsnode2066.members.1.js
11
859058
species

1
168934
genus

node2068.members.0.js
1
168935
species

1910391104
1612157
genus

node2070.members.1.jsnode2070.members.2.jsnode2070.members.3.jsnode2070.members.4.js
1910391104
1084
species

2589
13134
genus

node2072.members.2.js
3
84159
species

node2073.members.1.jsnode2073.members.2.js
2586
55518
species

2
1081
genus

node2075.members.2.js
2
1085
species

1
390876
genus

node2077.members.2.js
1
390877
species

3
991903
genus

node2079.members.2.js
3
991904
species

node2080.members.0.jsnode2080.members.1.js
2254
1229484
species

node2081.members.0.jsnode2081.members.1.jsnode2081.members.3.js
8126710
1234595
species

node2082.members.1.jsnode2082.members.2.js
550
710804
species

node2083.members.0.jsnode2083.members.1.jsnode2083.members.4.js
281
1229204
species

node2084.members.0.jsnode2084.members.1.jsnode2084.members.2.jsnode2084.members.3.jsnode2084.members.4.js
1061421611661226911
13113127621
204457
order

node2085.members.0.jsnode2085.members.1.jsnode2085.members.3.jsnode2085.members.4.js
104741921074914092
21856534
41297
family

node2086.members.2.js
1
95606
species

node2087.members.1.jsnode2087.members.4.js
501
341
165697
genus

node2088.members.1.js
14
1502850
species

node2089.members.1.js
2
117207
species

14
150203
genus

node2091.members.0.jsnode2091.members.2.js
14
1419876
species

node2092.members.0.jsnode2092.members.1.jsnode2092.members.4.js
313161912
11412
165696
genus

node2093.members.0.js
2
1144305
species

node2094.members.0.js
2
1206458
species

node2095.members.0.jsnode2095.members.1.js
1561
1329895
species

node2096.members.0.jsnode2096.members.1.jsnode2096.members.2.js
9118
702113
species

node2097.members.1.js
3
158500
species

node2098.members.1.jsnode2098.members.2.js
21
555793
species

node2099.members.0.js
28
48936
species

node2100.members.1.js
2
169176
species

node2101.members.0.jsnode2101.members.1.jsnode2101.members.3.js
261
1609758
species

node2102.members.0.jsnode2102.members.1.jsnode2102.members.3.js
453174242013979
18838210
165695
genus

node2103.members.2.js
3
76947
species

node2104.members.0.jsnode2104.members.1.jsnode2104.members.3.js
5421
121428
species

node2105.members.0.jsnode2105.members.1.js
431
420994
species

node2106.members.0.js
2
1522072
species

node2107.members.0.js
10
522773
species

node2108.members.0.jsnode2108.members.1.jsnode2108.members.3.js
10512659
13690
species

node2109.members.0.jsnode2109.members.1.jsnode2109.members.4.js
2213965
332056
species

node2110.members.0.jsnode2110.members.1.js
401
1335760
species

node2111.members.0.jsnode2111.members.2.jsnode2111.members.4.js
4811
1207055
species

node2112.members.1.js
5
1144307
species

node2113.members.0.jsnode2113.members.1.js
444
1332080
species

node2114.members.0.jsnode2114.members.4.js
113
1461752
species

node2115.members.0.js
5
627192
species

node2116.members.0.jsnode2116.members.1.jsnode2116.members.2.jsnode2116.members.3.jsnode2116.members.4.js
6218197925106
88952154
13687
genus

node2117.members.0.jsnode2117.members.1.js
11
1381597
species

node2118.members.0.jsnode2118.members.1.jsnode2118.members.4.js
12100
1549858
species

node2119.members.1.js
1
1007104
species

node2120.members.0.jsnode2120.members.1.js
62
397260
species

node2121.members.2.js
1
1550073
species

node2122.members.2.js
23
1564113
species

node2123.members.1.js
1
1112214
species

node2124.members.0.js
1
194867
species

node2125.members.1.jsnode2125.members.3.jsnode2125.members.4.js
67661
1112216
species

node2126.members.0.jsnode2126.members.1.js
52
13689
species

node2127.members.0.jsnode2127.members.1.js
21
745310
species

node2128.members.0.js
2
1112213
species

node2129.members.1.js
1
685778
species

node2130.members.0.js
6
152682
species

node2131.members.3.js
1
1449076
species

node2132.members.0.js
1
1283312
species

node2133.members.1.jsnode2133.members.3.js
1512
104605
species

node2134.members.0.jsnode2134.members.1.jsnode2134.members.3.js
3861
473781
species

node2135.members.0.jsnode2135.members.2.jsnode2135.members.4.js
5531
160791
species

node2136.members.0.js
1
907061
species

node2137.members.0.js
17
322611
species

node2138.members.0.js
3
314266
species

node2139.members.1.jsnode2139.members.2.jsnode2139.members.3.jsnode2139.members.4.js
113611185198
11611178435
335929
family

6
1111
genus

node2141.members.4.js
5
1248917
species

node2142.members.4.js
1
1479239
species

node2143.members.1.jsnode2143.members.4.js
126757
23436
1041
genus

node2144.members.4.js
1209
383381
species

node2145.members.0.jsnode2145.members.4.js
12112
39960
species

node2146.members.0.jsnode2146.members.1.jsnode2146.members.2.jsnode2146.members.3.jsnode2146.members.4.js
83656750264030740906
12617326316914
356
order

1
772
family

1
773
genus

node2149.members.2.js
1
373638
species

3
1484898
genus

node2151.members.2.js
3
1384459
species

1841123256417
118882
family

121
528
genus

node2154.members.0.jsnode2154.members.4.js
121
529
species

node2155.members.2.jsnode2155.members.3.js
531122876416
18472
234
genus

node2156.members.1.jsnode2156.members.2.jsnode2156.members.3.jsnode2156.members.4.js
158828335
29461
species

node2157.members.0.jsnode2157.members.1.jsnode2157.members.2.jsnode2157.members.3.jsnode2157.members.4.js
12516002911
120576
species

node2158.members.0.js
4
235
species

node2159.members.2.js
11
520449
species

node2160.members.2.js
1
120577
species

1138
354349
genus

node2162.members.0.jsnode2162.members.1.jsnode2162.members.2.js
1138
1201036
species

node2163.members.0.jsnode2163.members.2.js
430
22
31993
family

node2164.members.2.js
110
1
425
genus

node2165.members.2.js
6
136993
species

node2166.members.2.js
3
426
species

node2167.members.0.js
1
107635
species

15
133
genus

node2169.members.2.js
15
622637
species

3
61653
genus

node2171.members.2.js
3
1120792
species

1
261933
genus

node2173.members.0.js
1
261934
species

10
255475
family

10
182269
genus

node2176.members.2.js
10
651183
species

node2177.members.0.jsnode2177.members.2.js
119536
29
69277
family

11
274591
genus

node2179.members.1.js
1
1547437
species

node2180.members.2.js
1
1116369
species

node2181.members.0.jsnode2181.members.1.jsnode2181.members.2.js
88526
76474
68287
genus

node2182.members.2.js
1
1287322
species

node2183.members.2.js
2
381
species

node2184.members.1.js
1
39645
species

node2185.members.2.js
2
1287292
species

node2186.members.0.js
1
1566387
species

node2187.members.2.js
5
489722
species

node2188.members.1.js
1
1287085
species

node2189.members.2.js
2
69974
species

node2190.members.2.js
1
1505933
species

node2191.members.2.js
3
1287313
species

node2192.members.2.js
9
1287252
species

node2193.members.2.js
27
1287334
species

1
449972
genus

node2195.members.0.js
1
266779
species

730
45401
family

node2197.members.2.js
1
1068
genus

19
81
genus

node2199.members.2.js
19
1427356
species

710
46913
genus

node2201.members.1.jsnode2201.members.2.js
710
46914
species

15
119043
family

1
643217
genus

node2204.members.2.js
1
209897
species

1
256616
genus

node2206.members.0.js
1
256618
species

1
580880
genus

node2208.members.2.js
1
340680
species

3
1358438
genus

node2210.members.2.js
3
1358440
species

node2211.members.0.js
317
1
335928
family

node2212.members.2.js
12
10
279
genus

node2213.members.2.js
1
280
species

node2214.members.2.js
1
1117244
species

15
152053
genus

node2216.members.0.jsnode2216.members.2.js
15
921
species

1
6
genus

node2218.members.0.js
1
281091
species

node2219.members.0.jsnode2219.members.1.jsnode2219.members.2.jsnode2219.members.3.js
16034142711
32218311
82115
family

node2220.members.0.jsnode2220.members.1.jsnode2220.members.2.jsnode2220.members.4.js
11363801
681521
379
genus

node2221.members.2.js
4
1353277
species

node2222.members.2.js
8
196607
species

node2223.members.2.js
12
424182
species

node2224.members.0.jsnode2224.members.2.js
21
1223565
species

node2225.members.2.js
1
29449
species

node2226.members.2.js
2
1500305
species

node2227.members.2.js
5
1500306
species

node2228.members.0.jsnode2228.members.1.js
151
1510041
species

node2229.members.0.jsnode2229.members.2.js
21
384
species

node2230.members.2.js
3
1144312
species

node2231.members.2.js
9
398
species

node2232.members.0.jsnode2232.members.2.js
1282
391
species

node2233.members.0.js
24
1246459
species

node2234.members.0.js
1
78527
species

node2235.members.1.js
4
1125979
species

3
166953
genus

node2237.members.2.js
3
363259
species

node2238.members.0.js
21
1
28105
genus

node2239.members.0.js
1
380
species

node2240.members.2.js
1
382
species

10
323620
genus

node2242.members.1.js
10
1410620
species

5
1525371
genus

node2244.members.0.js
5
399
species

node2245.members.0.jsnode2245.members.1.js
810712
627
357
genus

node2246.members.1.js
9
28099
species

node2247.members.1.js
63
160699
species

node2248.members.2.js
11
359
species

281
1183400
species group

node2250.members.0.jsnode2250.members.1.jsnode2250.members.2.js
281
358
species

node2251.members.0.jsnode2251.members.2.jsnode2251.members.3.js
1691
710780
species

node2252.members.2.jsnode2252.members.3.jsnode2252.members.4.js
155135739897
10314357
119045
family

node2253.members.0.jsnode2253.members.1.jsnode2253.members.2.jsnode2253.members.3.jsnode2253.members.4.js
154112425421
94122502
407
genus

node2254.members.4.js
10
648885
species

node2255.members.2.jsnode2255.members.4.js
21
223967
species

node2256.members.2.js
1
39956
species

21015175
578822
species group

node2258.members.0.jsnode2258.members.2.jsnode2258.members.4.js
21015175
408
species

node2259.members.2.jsnode2259.members.3.jsnode2259.members.4.js
8429733
270351
species

node2260.members.2.js
2
1166158
species

node2261.members.0.js
4
1305730
species

node2262.members.2.js
1
1096546
species

node2263.members.1.jsnode2263.members.2.js
113119
14
186650
genus

node2264.members.4.js
119
1549810
species

node2265.members.2.js
4
864069
species

node2266.members.2.js
4
217168
species

node2267.members.2.js
1
420324
species

node2268.members.0.jsnode2268.members.1.jsnode2268.members.2.jsnode2268.members.3.jsnode2268.members.4.js
49626362733225977
19253997527101
41294
family

node2269.members.0.jsnode2269.members.2.jsnode2269.members.3.js
2611073841
1131553
1033
genus

node2270.members.2.js
7
1429916
species

node2271.members.0.jsnode2271.members.2.jsnode2271.members.3.jsnode2271.members.4.js
3692211
56946
species

node2272.members.2.js
92
666684
species

node2273.members.0.jsnode2273.members.1.jsnode2273.members.2.js
21547
1035
species

node2274.members.2.js
6
1034
species

node2275.members.0.jsnode2275.members.2.js
109
151414
species

node2276.members.2.js
8
709797
species

node2277.members.2.js
14510831
3
1073
genus

node2278.members.0.jsnode2278.members.2.js
112
95607
species

node2279.members.0.jsnode2279.members.1.jsnode2279.members.2.jsnode2279.members.3.js
13510681
1076
species

node2280.members.2.js
219792
1
911
genus

node2281.members.2.js
15
314253
species

node2282.members.0.jsnode2282.members.2.jsnode2282.members.3.js
219632
912
species

330
40136
genus

node2284.members.2.js
330
40137
species

node2285.members.0.jsnode2285.members.1.jsnode2285.members.2.jsnode2285.members.3.jsnode2285.members.4.js
26112308619191875
1469268005168833
374
genus

node2286.members.0.jsnode2286.members.2.js
165
443598
species

node2287.members.0.jsnode2287.members.2.js
122
1223566
species

node2288.members.0.jsnode2288.members.2.js
699
196490
species

node2289.members.0.jsnode2289.members.2.js
558
288000
species

node2290.members.0.jsnode2290.members.2.js
118
1040989
species

node2291.members.2.js
1
1268334
species

node2292.members.0.jsnode2292.members.2.js
286
551947
species

node2293.members.2.js
66
1380352
species

node2294.members.2.js
38
319017
species

node2295.members.2.js
1
455353
species

node2296.members.2.js
172
319003
species

node2297.members.2.js
2
1040988
species

node2298.members.2.js
36
754500
species

node2299.members.0.jsnode2299.members.2.js
2140
1298867
species

node2300.members.2.js
45
1188263
species

node2301.members.2.js
43
502063
species

node2302.members.2.js
18
189753
species

node2303.members.2.js
22
44255
species

node2304.members.2.jsnode2304.members.3.jsnode2304.members.4.js
1274952
1297865
species

node2305.members.0.jsnode2305.members.2.js
118
108015
species

node2306.members.0.jsnode2306.members.2.js
2017
1144344
species

node2307.members.2.js
184
1038867
species

node2308.members.2.js
47
551936
species

node2309.members.0.jsnode2309.members.2.jsnode2309.members.4.js
114457
172088
species

node2310.members.0.jsnode2310.members.2.js
210
1038866
species

node2311.members.0.jsnode2311.members.1.jsnode2311.members.2.jsnode2311.members.3.jsnode2311.members.4.js
291149221518
29448
species

node2312.members.2.js
1
1128179
species

node2313.members.0.jsnode2313.members.2.js
525
1126627
species

node2314.members.2.js
1
244563
species

node2315.members.2.js
64
115808
species

node2316.members.2.js
40
318996
species

node2317.members.0.jsnode2317.members.2.js
1444
375
species

node2318.members.2.js
51
43992
species

node2319.members.2.js
1575
335659
species

node2320.members.2.js
465
1355477
species

node2321.members.0.jsnode2321.members.2.js
122
1094929
species

node2322.members.0.jsnode2322.members.2.jsnode2322.members.3.js
24011
113395
species

node2323.members.1.jsnode2323.members.2.js
117
1380355
species

node2324.members.2.js
4
1128178
species

node2325.members.0.jsnode2325.members.1.jsnode2325.members.2.jsnode2325.members.3.jsnode2325.members.4.js
1617260215
1230476
species

node2326.members.2.js
20
566679
species

node2327.members.1.js
131
2
85413
genus

node2328.members.2.js
1
1502851
species

node2329.members.0.jsnode2329.members.1.js
11
1510531
species

123
45404
family

1
1143004
genus

node2332.members.2.js
1
876270
species

122
184923
genus

node2334.members.0.jsnode2334.members.1.jsnode2334.members.2.js
122
133552
species

2533365333214
204458
order

node2336.members.0.jsnode2336.members.1.jsnode2336.members.3.js
2533365333214
171881
76892
family

23
20
genus

node2338.members.0.jsnode2338.members.1.js
23
284016
species

node2339.members.0.jsnode2339.members.1.js
5910
545
75
genus

node2340.members.1.js
4
1298862
species

node2341.members.0.jsnode2341.members.1.js
41
69395
species

node2342.members.0.js
1
1449049
species

node2343.members.0.jsnode2343.members.1.jsnode2343.members.3.jsnode2343.members.4.js
1753160331214
147217426163
41275
genus

node2344.members.0.jsnode2344.members.1.jsnode2344.members.3.js
132854
293
species

node2345.members.0.jsnode2345.members.1.js
17
1495854
species

node2346.members.0.js
9
1125965
species

node2347.members.0.jsnode2347.members.1.jsnode2347.members.2.jsnode2347.members.4.js
140735
172043
species

node2348.members.1.js
7
74313
species

node2349.members.0.jsnode2349.members.1.jsnode2349.members.3.js
42771
588932
species

node2350.members.4.js
1
370977
species

node2351.members.1.js
1
74311
species

node2352.members.1.jsnode2352.members.4.js
245
391600
species

node2353.members.1.jsnode2353.members.3.js
41
1382303
species

node2354.members.0.js
94448168259
29
766
order

24
84646
genus

node2356.members.0.js
24
84647
species

node2357.members.0.js
27
1
1521255
genus

node2358.members.0.js
26
91604
species

node2359.members.0.js
63748168259
22
775
family

node2360.members.0.js
61548168259
1
33988
tribe

node2361.members.0.jsnode2361.members.2.jsnode2361.members.3.jsnode2361.members.4.js
61348168259
2001694146
780
genus

115
114292
species group

node2363.members.0.jsnode2363.members.2.js
115
782
species

node2364.members.0.js
41231212213
234
114277
species group

node2365.members.0.js
3
787
species

node2366.members.0.jsnode2366.members.2.jsnode2366.members.3.jsnode2366.members.4.js
15731212213
42862
species

node2367.members.0.js
1
35791
species

node2368.members.0.js
5
467174
species

node2369.members.0.js
1
33989
species

node2370.members.0.js
1
1068590
species

node2371.members.0.js
1
1462938
species

node2372.members.0.js
1
786
species

node2373.members.0.js
1
334545
species

node2374.members.0.js
3
354585
species

node2375.members.0.js
4
444612
species

1
69474
genus

node2377.members.0.js
1
784
species

node2378.members.0.js
67
54
44746
family

2
44747
genus

node2380.members.0.js
1
431043
species

node2381.members.0.js
1
1169117
species

node2382.members.0.js
4
86105
species

node2383.members.0.js
7
86106
species

160
942
family

160
952
tribe

node2386.members.0.js
160
5
953
genus

node2387.members.0.js
152
188349
species

node2388.members.0.js
1
263437
species

node2389.members.0.js
1
77038
species

node2390.members.0.js
1
246273
species

1
1191478
order

1
1191479
family

1
162171
genus

node2394.members.2.js
1
1124597
species

1781001
204455
order

11
69657
family

node2397.members.1.jsnode2397.members.2.js
11
85
genus

node2398.members.0.jsnode2398.members.1.jsnode2398.members.2.js
177991
212
31989
family

1
191028
genus

node2400.members.0.js
1
1577895
species

1
1470577
genus

node2402.members.1.js
1
1105367
species

node2403.members.0.js
451
1
265
genus

node2404.members.1.js
1
59779
species

node2405.members.0.js
3
225362
species

node2406.members.1.js
4
82367
species

node2407.members.3.js
1
1569209
species

4
295418
genus

node2409.members.0.js
4
1123067
species

4
1060
genus

node2411.members.0.js
4
1061
species

2
282682
genus

node2413.members.0.js
2
501479
species

49
285107
genus

node2415.members.2.js
49
1185766
species

32
2433
genus

node2417.members.2.js
32
351016
species

1
225421
genus

node2419.members.2.js
1
225422
species

node2420.members.2.js
5
314270
species

9
258255
genus

node2422.members.2.js
9
439495
species

node2423.members.2.js
1
1366046
species

node2424.members.0.jsnode2424.members.1.jsnode2424.members.2.jsnode2424.members.3.jsnode2424.members.4.js
171861115097186894127471581485
3167139130407927688
1236
class

3
1400857
family

3
1400859
genus

node2427.members.0.js
3
1400860
species

94615321
135625
order

node2429.members.1.jsnode2429.members.2.jsnode2429.members.4.js
94615321
181
712
family

10
416916
genus

node2431.members.2.js
10
714
species

382
75984
genus

node2433.members.1.jsnode2433.members.3.js
372
75985
species

node2434.members.1.js
1
85404
species

1
658623
genus

node2436.members.1.js
1
505317
species

node2437.members.0.jsnode2437.members.1.jsnode2437.members.2.js
96135
9313
724
genus

node2438.members.1.js
3
729
species

node2439.members.2.js
1
726
species

node2440.members.2.js
121
727
species

node2441.members.4.js
1
939696
species

20323841
135624
order

20323841
84642
family

node2444.members.0.js
20323841
16
642
genus

node2445.members.2.js
18
591962
species

node2446.members.0.jsnode2446.members.2.jsnode2446.members.3.js
222791
645
species

node2447.members.0.jsnode2447.members.1.jsnode2447.members.2.js
2387
644
species

1
135615
order

1
868
family

1
2717
genus

node2451.members.3.js
1
2718
species

3229301
135618
order

11
1486721
family

11
244364
genus

node2455.members.0.jsnode2455.members.2.js
11
244365
species

2229291
403
family

1
160800
genus

node2458.members.0.js
1
105972
species

node2459.members.2.js
1
416
genus

11
429
genus

node2461.members.0.jsnode2461.members.2.js
11
173365
species

229271
413
genus

node2463.members.1.jsnode2463.members.2.jsnode2463.members.3.js
229271
414
species

node2464.members.1.jsnode2464.members.4.js
7232183640
154
135622
order

515141
267888
family

node2466.members.1.js
515141
4
53246
genus

node2467.members.1.js
6
228
species

node2468.members.1.js
3
394751
species

node2469.members.1.js
1
1333520
species

node2470.members.1.js
1
304208
species

node2471.members.0.jsnode2471.members.2.jsnode2471.members.4.js
5141
176102
species

1130132
267890
family

node2473.members.0.jsnode2473.members.4.js
1130132
12
22
genus

node2474.members.2.js
1
60961
species

node2475.members.1.jsnode2475.members.3.js
1303
256839
species

11
267893
family

node2477.members.2.js
11
1
135575
genus

node2478.members.4.js
1
190892
species

node2479.members.4.js
1722632
1
72275
family

630
1172191
genus

node2481.members.4.js
630
1172192
species

node2482.members.1.js
70
24
2742
genus

node2483.members.1.js
32
1479237
species

node2484.members.1.js
14
1150997
species

12
1621534
genus

node2486.members.2.js
2
326544
species

node2487.members.1.js
1
1128911
species

node2488.members.1.jsnode2488.members.4.js
111
11
226
genus

node2489.members.0.js
1
28108
species

6772024113
118969
order

node2491.members.0.js
6572024113
29
444
family

node2492.members.0.js
3672024113
30
445
genus

node2493.members.0.js
272024113
1
446
species

node2494.members.0.jsnode2494.members.1.jsnode2494.members.2.jsnode2494.members.3.jsnode2494.members.4.js
172024113
91891
subspecies

node2495.members.0.js
3
168933
species

node2496.members.0.js
1
29423
species

2
118968
family

2
776
genus

node2499.members.0.js
2
777
species

8361493263313
135614
order

23
568386
family

1
1274363
genus

node2503.members.2.js
1
616992
species

22
64001
genus

node2505.members.2.js
22
418856
species

node2506.members.0.jsnode2506.members.1.jsnode2506.members.2.jsnode2506.members.3.js
8361493240313
10381473
32033
family

1
490567
genus

node2508.members.0.js
1
498055
species

2
231454
genus

node2510.members.4.js
2
1379159
species

node2511.members.0.jsnode2511.members.1.js
5655810
2711
40323
genus

node2512.members.0.jsnode2512.members.1.jsnode2512.members.4.js
5364510
40324
species

node2513.members.0.jsnode2513.members.1.js
12
216778
species

node2514.members.0.js
1
391601
species

node2515.members.0.jsnode2515.members.1.jsnode2515.members.2.js
154931871
7472
338
genus

node2516.members.2.jsnode2516.members.4.js
3051
56448
species

22880
643453
species group

node2518.members.1.jsnode2518.members.2.js
22880
346
species

node2519.members.0.js
1
29446
species

node2520.members.0.js
2
56450
species

node2521.members.0.js
76
53413
species

node2522.members.0.js
1
339
species

1
83614
genus

node2524.members.0.js
1
935863
species

16
75309
genus

node2526.members.2.js
1
416169
species

node2527.members.2.js
4
1076524
species

node2528.members.0.jsnode2528.members.2.js
11
219571
species

11
2370
genus

node2530.members.0.js
11
2371
species

1
68
genus

node2532.members.1.js
1
435897
species

8083618351942118
91347
order

node2534.members.0.jsnode2534.members.1.jsnode2534.members.2.jsnode2534.members.3.jsnode2534.members.4.js
8083618351942118
251358743410
543
family

node2535.members.1.js
1
635
genus

1
451512
genus

node2537.members.1.js
1
1224318
species

1
413496
genus

node2539.members.2.js
1
413503
species

node2540.members.0.jsnode2540.members.2.js
252144
58
547
genus

node2541.members.2.js
2026
1
354276
species group

node2542.members.0.jsnode2542.members.1.jsnode2542.members.2.js
2025
550
species

node2543.members.2.js
130
548
species

6131
570
genus

node2545.members.0.jsnode2545.members.2.jsnode2545.members.4.js
6121
573
species

node2546.members.2.js
1
936565
species

1
84565
genus

node2548.members.0.js
1
63612
species

109351
590
genus

node2550.members.0.jsnode2550.members.1.js
109351
113
28901
species

node2551.members.0.jsnode2551.members.2.jsnode2551.members.4.js
9851
59201
subspecies

1
568988
genus

node2553.members.0.js
1
138073
species

211025
122277
genus

node2555.members.0.jsnode2555.members.1.jsnode2555.members.2.js
211025
554
species

node2556.members.0.js
11
1
586
genus

node2557.members.2.js
1
588
species

272
620
genus

node2559.members.2.jsnode2559.members.4.js
272
623
species

node2560.members.0.js
18
1
53335
genus

node2561.members.1.js
8
1484157
species

node2562.members.0.jsnode2562.members.1.jsnode2562.members.2.js
103567456936103
1131
561
genus

node2563.members.1.jsnode2563.members.2.js
3420
469598
species

node2564.members.0.jsnode2564.members.1.jsnode2564.members.2.jsnode2564.members.3.jsnode2564.members.4.js
102527411836103
562
species

node2565.members.0.jsnode2565.members.1.jsnode2565.members.2.jsnode2565.members.3.js
915262
912561
613
genus

node2566.members.1.js
25
24
615
species

node2567.members.1.js
1
211759
subspecies

node2568.members.1.js
2
47917
species

node2569.members.3.js
1
82996
species

2191
568987
genus

node2571.members.0.jsnode2571.members.4.js
2191
138072
species

571
544
genus

node2573.members.0.js
57
1563222
species

node2574.members.1.js
1
545
species

45
637
genus

node2576.members.2.js
5
1247023
species

node2577.members.0.js
4
638
species

3382
135619
order

1
224379
family

1
305899
genus

node2581.members.4.js
1
1027273
species

341
28256
family

341
2745
genus

node2584.members.1.js
22
664683
species

node2585.members.4.js
1
1121960
species

node2586.members.1.js
12
176290
species

34
135620
family

3
267849
genus

node2589.members.0.js
3
1229521
species

node2590.members.1.js
1
1330036
species

3
187492
genus

node2592.members.1.js
3
187493
species

node2593.members.0.jsnode2593.members.1.jsnode2593.members.2.jsnode2593.members.3.jsnode2593.members.4.js
14900110686572006126081552939
193384268
72274
order

node2594.members.0.jsnode2594.members.2.jsnode2594.members.4.js
2089236157536592
911651
135621
family

node2595.members.0.jsnode2595.members.1.jsnode2595.members.2.jsnode2595.members.3.jsnode2595.members.4.js
2080236156534941
115914263430351
286
genus

node2596.members.4.js
3
101564
species

node2597.members.2.js
1
95619
species

2641633
136846
species group

node2599.members.4.js
3
47886
species

node2600.members.1.js
41
271420
species

2663
578833
species subgroup

node2602.members.0.jsnode2602.members.2.js
2663
316
species

node2603.members.1.js
1
157783
species

node2604.members.4.js
21
115714
species

node2605.members.4.js
4
1197727
species

458
136845
species group

node2607.members.0.js
457
303
species

node2608.members.0.js
1
76759
species

2
136844
species group

node2610.members.1.js
2
43306
species

node2611.members.0.js
1
470150
species

node2612.members.0.js
2
157782
species

389501314557
136841
species group

node2614.members.1.js
1
53412
species

32
1232139
species subgroup

node2616.members.4.js
32
330
species

node2617.members.0.jsnode2617.members.1.jsnode2617.members.2.jsnode2617.members.4.js
3882134524
287
species

471
627141
species subgroup

node2619.members.1.jsnode2619.members.3.js
471
46680
species

node2620.members.0.jsnode2620.members.4.js
11
300
species

node2621.members.0.js
3216
2
136849
species group

node2622.members.0.jsnode2622.members.2.js
2715
251701
species

1
251698
species subgroup

node2624.members.0.js
1
29438
species

21
251695
species subgroup

node2626.members.0.jsnode2626.members.2.js
21
317
species

node2627.members.0.js
12
7
136843
species group

node2628.members.0.js
1
183795
species

node2629.members.0.js
1
294
species

node2630.members.0.js
3
75612
species

node2631.members.4.js
2
1234594
species

node2632.members.0.js
1
911240
species

node2633.members.0.jsnode2633.members.1.jsnode2633.members.2.jsnode2633.members.3.jsnode2633.members.4.js
12792110629171845126011516279
21940231188508
468
family

node2634.members.1.js
2711
27
497
genus

node2635.members.4.js
1
1002339
species

node2636.members.2.js
1
248452
species

3216
475
genus

31
46225
subgenus

node2639.members.1.js
31
77152
species

116
46226
subgenus

node2641.members.1.jsnode2641.members.2.js
116
480
species

221
222991
genus

node2643.members.1.jsnode2643.members.4.js
221
197183
species

node2644.members.0.jsnode2644.members.1.jsnode2644.members.2.jsnode2644.members.3.jsnode2644.members.4.js
12771110527071805125901427769
54618165873818290301025051
469
genus

node2645.members.0.jsnode2645.members.1.jsnode2645.members.2.js
1141
1144661
species

node2646.members.0.jsnode2646.members.1.jsnode2646.members.2.jsnode2646.members.3.js
3758112
1144671
species

node2647.members.1.jsnode2647.members.2.js
212
1217692
species

node2648.members.0.jsnode2648.members.1.jsnode2648.members.2.jsnode2648.members.3.jsnode2648.members.4.js
136548362475322
202952
species

node2649.members.1.jsnode2649.members.2.js
1915
108980
species

node2650.members.1.js
8
1217706
species

node2651.members.1.jsnode2651.members.2.js
102
1397525
species

node2652.members.1.jsnode2652.members.2.js
1379
1144663
species

node2653.members.0.jsnode2653.members.1.jsnode2653.members.2.jsnode2653.members.3.jsnode2653.members.4.js
26149516931076
40215
species

node2654.members.1.jsnode2654.members.3.js
2183
466088
species

node2655.members.1.js
14
1217709
species

node2656.members.0.jsnode2656.members.1.jsnode2656.members.3.jsnode2656.members.4.js
6358414
1485002
species

node2657.members.1.js
1
520709
species

node2658.members.0.jsnode2658.members.2.js
319
1144674
species

node2659.members.1.jsnode2659.members.2.js
111
1217699
species

node2660.members.0.jsnode2660.members.1.jsnode2660.members.2.jsnode2660.members.3.jsnode2660.members.4.js
2823412212
106649
species

node2661.members.0.jsnode2661.members.1.jsnode2661.members.2.jsnode2661.members.3.jsnode2661.members.4.js
2326157133
756892
species

node2662.members.1.jsnode2662.members.2.jsnode2662.members.4.js
14185
134533
species

node2663.members.1.js
8
1221303
species

node2664.members.1.js
3
1144668
species

node2665.members.1.jsnode2665.members.2.jsnode2665.members.3.js
92331
1144664
species

node2666.members.1.jsnode2666.members.3.js
141
1217703
species

node2667.members.0.jsnode2667.members.1.jsnode2667.members.2.jsnode2667.members.3.jsnode2667.members.4.js
613183117336
1509403
species

node2668.members.1.jsnode2668.members.2.jsnode2668.members.3.jsnode2668.members.4.js
16501687172
202956
species

node2669.members.1.js
2
1217698
species

node2670.members.0.js
1
1262690
species

node2671.members.1.js
10
1217695
species

node2672.members.1.js
3
1221304
species

node2673.members.1.js
16
1561217
species

node2674.members.1.js
2
520708
species

node2675.members.1.jsnode2675.members.3.js
1242
1144672
species

node2676.members.1.jsnode2676.members.2.js
335
1144665
species

node2677.members.4.js
1
1144662
species

node2678.members.0.jsnode2678.members.1.jsnode2678.members.2.jsnode2678.members.3.js
141137917
202954
species

node2679.members.1.js
1
1217705
species

node2680.members.0.jsnode2680.members.2.js
13
1144673
species

node2681.members.1.jsnode2681.members.2.js
32
1219382
species

node2682.members.0.jsnode2682.members.1.jsnode2682.members.2.js
221131
40216
species

node2683.members.1.js
7
192995
species

node2684.members.1.jsnode2684.members.2.jsnode2684.members.3.js
35324
202955
species

node2685.members.1.js
2
1148157
species

node2686.members.0.jsnode2686.members.1.jsnode2686.members.2.jsnode2686.members.3.jsnode2686.members.4.js
768486227
1217704
species

node2687.members.0.jsnode2687.members.1.jsnode2687.members.2.jsnode2687.members.3.jsnode2687.members.4.js
24401244
1217710
species

node2688.members.1.js
11
1217707
species

node2689.members.1.jsnode2689.members.2.js
111
525244
species

node2690.members.1.jsnode2690.members.3.js
41
1501691
species

node2691.members.1.js
8
1217694
species

node2692.members.1.jsnode2692.members.3.js
231
262668
species

node2693.members.1.js
1
1071390
species

node2694.members.0.jsnode2694.members.1.jsnode2694.members.2.js
414301
1217701
species

node2695.members.1.js
1
752244
species

node2696.members.0.jsnode2696.members.1.jsnode2696.members.2.jsnode2696.members.4.js
38816272
108981
species

node2697.members.0.jsnode2697.members.1.jsnode2697.members.2.jsnode2697.members.3.jsnode2697.members.4.js
1826291
106648
species

node2698.members.1.js
30
396323
species

node2699.members.0.jsnode2699.members.1.jsnode2699.members.2.jsnode2699.members.3.jsnode2699.members.4.js
3312139321
487316
species

node2700.members.1.jsnode2700.members.2.js
83
1217693
species

node2701.members.0.jsnode2701.members.1.jsnode2701.members.2.jsnode2701.members.3.jsnode2701.members.4.js
29422491290623
1217711
species

node2702.members.1.js
2
107402
species

node2703.members.1.js
1
1499942
species

node2704.members.1.jsnode2704.members.3.jsnode2704.members.4.js
87542895
1217713
species

node2705.members.0.jsnode2705.members.1.jsnode2705.members.3.jsnode2705.members.4.js
3911256012117
40214
species

node2706.members.1.js
15
1217700
species

node2707.members.1.jsnode2707.members.2.jsnode2707.members.3.js
12221
1217702
species

node2708.members.0.jsnode2708.members.1.jsnode2708.members.4.js
1172
1209355
species

node2709.members.1.jsnode2709.members.2.jsnode2709.members.3.js
4694887
632955
species

node2710.members.1.jsnode2710.members.2.js
441
1550715
species

node2711.members.2.js
26
202950
species

node2712.members.0.jsnode2712.members.1.jsnode2712.members.2.jsnode2712.members.3.js
3114691
1029823
species

node2713.members.1.jsnode2713.members.4.js
511
1435036
species

node2714.members.1.js
4
1144669
species

node2715.members.1.js
5
1507807
species

node2716.members.1.jsnode2716.members.3.js
501
1513354
species

node2717.members.1.jsnode2717.members.2.js
18265
1280052
species

node2718.members.0.jsnode2718.members.1.jsnode2718.members.2.jsnode2718.members.3.jsnode2718.members.4.js
838212446252723289712
29430
species

node2719.members.0.jsnode2719.members.1.jsnode2719.members.2.jsnode2719.members.3.jsnode2719.members.4.js
1803846373315
28090
species

node2720.members.1.jsnode2720.members.2.js
28
52133
species

node2721.members.0.jsnode2721.members.1.jsnode2721.members.2.js
1321
903945
species

node2722.members.1.jsnode2722.members.2.jsnode2722.members.3.js
12241
134534
species

node2723.members.1.jsnode2723.members.2.js
97
1380368
species

node2724.members.1.js
13
619773
species

node2725.members.1.js
4
62977
species

node2726.members.1.js
2
1217714
species

node2727.members.1.jsnode2727.members.3.jsnode2727.members.4.js
1011
1144670
species

node2728.members.1.jsnode2728.members.3.js
111
1217708
species

node2729.members.1.js
3
1353941
species

node2730.members.0.jsnode2730.members.1.js
26
1217696
species

node2731.members.0.jsnode2731.members.1.js
124
202951
species

node2732.members.1.js
48
1217697
species

node2733.members.0.jsnode2733.members.1.jsnode2733.members.2.jsnode2733.members.3.jsnode2733.members.4.js
314284691568232419166
181114731023
909768
species group

node2734.members.1.js
15
903915
species

node2735.members.1.jsnode2735.members.2.jsnode2735.members.3.js
170452
48296
species

node2736.members.0.jsnode2736.members.1.jsnode2736.members.2.jsnode2736.members.3.jsnode2736.members.4.js
295264501539030319143
470
species

node2737.members.1.jsnode2737.members.2.js
311
903918
species

node2738.members.0.jsnode2738.members.1.jsnode2738.members.2.js
1321
471
species

node2739.members.1.jsnode2739.members.2.jsnode2739.members.3.js
6551729
106654
species

node2740.members.1.js
2
903916
species

node2741.members.1.jsnode2741.members.3.js
1271
1173062
species

node2742.members.1.jsnode2742.members.3.js
411
1217712
species

node2743.members.0.jsnode2743.members.4.js
91
2340
species

181129552665
135623
order

node2745.members.0.jsnode2745.members.2.jsnode2745.members.3.jsnode2745.members.4.js
181129552665
3101821
641
family

5392675150
657
genus

node2747.members.0.jsnode2747.members.1.jsnode2747.members.2.jsnode2747.members.3.jsnode2747.members.4.js
1392675150
121723
species

node2748.members.0.js
2
1056511
species

2
38293
species

node2750.members.0.js
2
85581
subspecies

10
511678
genus

node2752.members.2.js
10
688
species

node2753.members.1.js
10735849314
31
662
genus

node2754.members.0.jsnode2754.members.2.js
12
672
species

node2755.members.1.js
1
29494
species

node2756.members.1.js
11
674
species

node2757.members.4.js
12
1481922
species

node2758.members.1.jsnode2758.members.2.jsnode2758.members.3.jsnode2758.members.4.js
29582832
666
species

node2759.members.0.js
2
28173
species

7119
717610
species group

node2761.members.2.js
16
669
species

node2762.members.0.jsnode2762.members.1.jsnode2762.members.2.js
713
670
species

121882
1706369
order

1
1706373
family

1
48073
genus

node2766.members.1.js
1
266805
species

1211
1706371
family

node2768.members.0.jsnode2768.members.4.js
1211
10
genus

87
1706372
family

node2770.members.1.js
87
876044
species

1
1706375
family

node2772.members.4.js
1
247633
species

2
135613
order

1
72276
family

1
1765964
genus

node2776.members.0.js
1
160660
species

1
1046
family

1
85076
genus

node2779.members.0.js
1
37487
species

73552
72273
order

2
34064
family

node2782.members.4.js
2
1
262
genus

node2783.members.4.js
1
657445
species

3184
135617
family

218
1021
genus

node2786.members.0.jsnode2786.members.1.js
218
422289
species

1
40751
genus

node2788.members.0.js
1
40754
species

4
1030
genus

node2790.members.2.js
4
125627
species

4171
135616
family

4171
1237
genus

node2793.members.0.jsnode2793.members.1.jsnode2793.members.2.js
4171
1238
species

57
225057
order

57
225058
family

57
119977
genus

node2797.members.0.js
55
930
species

node2798.members.0.js
2
920
species

node2799.members.0.jsnode2799.members.1.jsnode2799.members.2.jsnode2799.members.3.jsnode2799.members.4.js
46903142136610366216112747
479146176332
28216
class

1
206350
order

1
32011
family

node2802.members.1.js
1
359407
genus

node2803.members.0.jsnode2803.members.1.jsnode2803.members.2.jsnode2803.members.3.jsnode2803.members.4.js
4639414194960066521481067
206437221911259408
80840
order

node2804.members.0.jsnode2804.members.1.jsnode2804.members.2.jsnode2804.members.3.jsnode2804.members.4.js
483492262910241429
3322761331
506
family

1
305976
genus

node2806.members.1.js
1
1007105
species

1
124224
genus

node2808.members.2.js
1
124225
species

node2809.members.1.js
12380
1
517
genus

node2810.members.0.js
1
94624
species

node2811.members.0.jsnode2811.members.1.jsnode2811.members.2.js
11280
518
species

1
312063
genus

node2813.members.1.js
1
364039
species

2562262810235428
29574
genus

node2815.members.0.jsnode2815.members.1.jsnode2815.members.2.jsnode2815.members.3.jsnode2815.members.4.js
2562262810235428
84590
species

node2816.members.1.js
106
4
507
genus

node2817.members.1.jsnode2817.members.2.js
66
511
species

node2818.members.0.jsnode2818.members.1.jsnode2818.members.3.js
1141393
85802
222
genus

node2819.members.0.jsnode2819.members.1.jsnode2819.members.3.js
29561
72556
species

node2820.members.1.js
2
1147684
species

node2821.members.1.js
1
1397275
species

node2822.members.2.js
14
1469502
species

72
212743
genus

node2824.members.0.jsnode2824.members.2.js
72
1298878
species

node2825.members.0.jsnode2825.members.1.jsnode2825.members.2.js
214453823
115131
75682
family

9
303379
genus

node2827.members.0.js
9
204773
species

node2828.members.2.js
40367
2
963
genus

node2829.members.0.jsnode2829.members.2.js
40365
1144342
species

node2830.members.0.jsnode2830.members.1.js
1531
1110
149698
genus

node2831.members.1.js
1
544911
species

node2832.members.0.jsnode2832.members.1.js
120
47229
species

node2833.members.0.js
1
286638
species

node2834.members.0.js
2
1549812
species

35114
29580
genus

node2836.members.0.jsnode2836.members.2.js
352
375286
species

node2837.members.1.js
1
1075768
species

node2838.members.2.js
12
1502762
species

3
75654
genus

node2840.members.3.js
3
75659
species

2
92793
genus

node2842.members.0.js
2
1538295
species

node2843.members.0.js
16268838510692
2
32012
genus

node2844.members.0.js
1
1158291
species

node2845.members.0.jsnode2845.members.1.jsnode2845.members.2.jsnode2845.members.3.jsnode2845.members.4.js
13268838510692
554131
species

node2846.members.0.jsnode2846.members.1.jsnode2846.members.2.jsnode2846.members.4.js
10442814349915
74511472
119060
family

node2847.members.0.js
1
93217
genus

node2848.members.0.jsnode2848.members.2.jsnode2848.members.4.js
69513938413
74121
32008
genus

node2849.members.2.js
1
1500897
species

node2850.members.0.js
1
1080179
species

node2851.members.0.jsnode2851.members.2.js
11
134537
species

node2852.members.4.js
1
412963
species

node2853.members.4.js
2
640511
species

node2854.members.2.js
2
337
species

node2855.members.0.jsnode2855.members.2.js
451303
15
87882
species group

node2856.members.1.jsnode2856.members.2.js
158
87883
species

node2857.members.0.js
36
292
species

node2858.members.0.jsnode2858.members.2.js
8240
95486
species

node2859.members.2.js
1
60547
species

node2860.members.2.js
1
870478
species

node2861.members.0.js
1
416344
species

node2862.members.0.jsnode2862.members.1.jsnode2862.members.2.jsnode2862.members.3.jsnode2862.members.4.js
1121310149
11786039
111527
species group

node2863.members.0.jsnode2863.members.1.jsnode2863.members.2.js
114604
28450
species

node2864.members.0.js
9
342113
species

node2865.members.2.js
241
57975
species

node2866.members.2.jsnode2866.members.3.js
3961
13373
species

node2867.members.2.js
98
312026
species

node2868.members.0.js
1
944435
species

node2869.members.2.js
1
392320
species

node2870.members.1.js
1
1071679
species

node2871.members.0.js
1
28095
species

node2872.members.2.js
7
935543
species

node2873.members.1.js
1
75105
species

node2874.members.0.jsnode2874.members.2.js
11
1097668
species

node2875.members.2.js
9
101571
species

node2876.members.0.js
2738
21
106589
genus

node2877.members.0.jsnode2877.members.1.jsnode2877.members.2.js
132
164546
species

node2878.members.0.jsnode2878.members.2.js
13
1268322
species

node2879.members.0.jsnode2879.members.2.js
32
68895
species

node2880.members.2.js
1
106590
species

node2881.members.0.js
1
119219
species

17
47670
genus

node2883.members.0.jsnode2883.members.1.js
17
47671
species

node2884.members.0.jsnode2884.members.1.jsnode2884.members.2.jsnode2884.members.3.js
201122565
14011251
48736
genus

node2885.members.0.jsnode2885.members.2.js
29
658080
species

node2886.members.1.js
8
28097
species

node2887.members.0.jsnode2887.members.1.jsnode2887.members.2.jsnode2887.members.3.js
493773
1235457
species

node2888.members.0.jsnode2888.members.2.js
24
329
species

node2889.members.2.js
1
1380362
species

node2890.members.0.jsnode2890.members.2.js
613
658664
species

node2891.members.0.jsnode2891.members.2.js
16
1217052
species

node2892.members.0.jsnode2892.members.2.jsnode2892.members.3.js
1211
305
species

node2893.members.0.jsnode2893.members.1.jsnode2893.members.2.jsnode2893.members.3.jsnode2893.members.4.js
44422140921111471527122
3107910025421108882
80864
family

672
151754
genus

node2895.members.1.jsnode2895.members.3.js
672
151755
species

1
335058
genus

node2897.members.0.js
1
1167741
species

5
52972
genus

node2899.members.0.js
5
296591
species

4
219181
genus

node2901.members.1.js
4
219182
species

node2902.members.0.js
127
10
34072
genus

node2903.members.0.jsnode2903.members.2.js
27
34073
species

1
433923
genus

node2905.members.0.js
1
433924
species

1
364316
genus

node2907.members.2.js
1
364317
species

node2908.members.0.js
1
1458425
species

node2909.members.0.jsnode2909.members.1.jsnode2909.members.2.jsnode2909.members.3.jsnode2909.members.4.js
1128839686741538
1008735554636834
12916
genus

node2910.members.0.jsnode2910.members.1.jsnode2910.members.2.jsnode2910.members.3.jsnode2910.members.4.js
119741141474
47920
species

node2911.members.1.js
1
1144317
species

node2912.members.0.jsnode2912.members.1.js
16
512030
species

node2913.members.0.js
1
232721
species

node2914.members.0.jsnode2914.members.1.js
12
80869
species

node2915.members.1.js
9
721785
species

node2916.members.0.js
1
358220
species

node2917.members.0.jsnode2917.members.1.js
135539212121
23
283
genus

node2918.members.1.js
5
1055192
species

node2919.members.0.jsnode2919.members.1.jsnode2919.members.2.jsnode2919.members.3.jsnode2919.members.4.js
135038412121
285
species

node2920.members.0.js
3
298265
species

node2921.members.3.js
2414110987
2
281915
genus

node2922.members.0.js
23
86182
species

node2923.members.0.jsnode2923.members.1.jsnode2923.members.2.jsnode2923.members.3.js
114110985
667019
species

node2924.members.0.jsnode2924.members.1.jsnode2924.members.3.jsnode2924.members.4.js
656503131
23431521
80865
genus

node2925.members.0.jsnode2925.members.1.js
1997
180282
species

node2926.members.0.jsnode2926.members.1.jsnode2926.members.2.jsnode2926.members.3.js
21615911
80866
species

node2927.members.0.jsnode2927.members.1.js
17
742013
species

node2928.members.0.jsnode2928.members.1.js
514
1468410
species

node2929.members.0.jsnode2929.members.1.js
11
1537702
species

1
201096
genus

node2931.members.1.js
1
179636
species

156531
995019
family

156531
40544
genus

node2934.members.2.jsnode2934.members.3.jsnode2934.members.4.js
156531
437898
species

437
206389
order

node2936.members.1.jsnode2936.members.2.js
437
11
75787
family

1
392735
genus

node2938.members.0.js
1
392736
species

node2939.members.0.js
1
1565605
species

node2940.members.0.js
15
1
33057
genus

node2941.members.2.js
1
1572758
species

node2942.members.2.js
4
76113
species

11
146935
genus

node2944.members.0.jsnode2944.members.2.js
11
146938
species

2
378210
genus

node2946.members.1.js
2
378211
species

104
327159
genus

node2948.members.0.js
10
1454002
species

node2949.members.2.js
4
1454005
species

2
713636
order

2
90627
family

2
96
genus

node2953.members.2.js
2
370405
species

161879251011678
206351
order

817511676
1499392
family

1
400947
genus

node2957.members.2.js
1
400948
species

3
535
genus

node2959.members.2.js
3
394935
species

811511676
568394
genus

node2961.members.0.jsnode2961.members.1.jsnode2961.members.3.jsnode2961.members.4.js
81511676
748280
species

node2962.members.2.js
1
990370
species

2
397275
genus

node2964.members.2.js
2
396808
species

node2965.members.2.js
817791852
5
481
family

1
538
genus

node2967.members.0.js
1
539
species

1
334107
genus

node2969.members.2.js
1
211502
species

17
32257
genus

node2971.members.1.js
17
1522312
species

node2972.members.0.jsnode2972.members.2.jsnode2972.members.3.js
7791252
15892
482
genus

node2973.members.2.jsnode2973.members.3.jsnode2973.members.4.js
730532
487
species

node2974.members.0.js
5
484
species

node2975.members.2.js
1
607711
species

node2976.members.2.js
1
267212
species

node2977.members.0.js
1
28449
species

node2978.members.2.js
2
489
species

node2979.members.2.js
14
485
species

node2980.members.1.js
2
417305
species

17
32003
order

17
206379
family

17
35798
genus

node2984.members.1.js
17
58133
species

node2985.members.1.js
2
86038
species

583
203691
phylum

583
203692
class

279
136
order

79
137
family

79
157
genus

node2991.members.2.js
1
158
species

78
53419
species

node2993.members.2.js
78
69714
subspecies

1
1643685
family

1
138
genus

1
64895
species group

node2997.members.0.js
1
29519
species

node2998.members.0.js
1
710994
species

34
170
family

node3000.members.0.js
33
2
171
genus

node3001.members.0.js
1
172
species

node3002.members.2.js
3
173
species

1
338321
genus

node3004.members.2.js
1
29510
species

6
508458
phylum

6
649775
class

6
649776
order

6
649777
family

3
46613
genus

node3010.members.2.js
3
97477
species

1
1434006
genus

node3012.members.2.js
1
651822
species

node3013.members.2.js
1
81466
genus

1
508459
genus

node3015.members.2.js
1
508460
species

26
67818
phylum

26
1454725
genus

node3018.members.0.jsnode3018.members.2.js
26
1454726
species

10315
131550
superphylum

node3020.members.0.js
10215
9
57723
phylum

node3021.members.0.js
281
1
1562566
class

261
1562565
genus

node3023.members.0.jsnode3023.members.1.js
261
454194
species

1
458032
genus

node3025.members.0.js
1
458033
species

101
204432
class

101
204433
order

node3028.members.0.js
101
3
204434
family

node3029.members.0.js
5
1267535
species

1
33973
genus

node3031.members.0.js
1
33075
species

node3032.members.2.js
1
1267534
species

1
940557
genus

node3034.members.0.js
1
940615
species

52
332159
class

52
332160
order

2
332161
family

2
332162
genus

node3039.members.0.js
2
332163
species

50
911113
genus

node3041.members.0.js
50
360054
species

node3042.members.0.js
1
1235986
species

4
533205
class

4
574975
order

4
574976
family

4
35838
genus

node3047.members.2.js
4
35839
species

node3048.members.0.js
2
171953
species

1
65842
phylum

1
204430
class

1
218872
order

1
204431
family

1
832
genus

1
833
species

node3055.members.0.js
1
834
subspecies

992
1297
phylum

node3057.members.0.jsnode3057.members.1.js
992
31
188787
class

5
68933
order

node3059.members.0.js
5
1
188786
family

2
65551
genus

node3061.members.0.js
1
307124
species

node3062.members.0.js
1
52023
species

node3063.members.0.js
2
1
270
genus

node3064.members.0.js
1
271
species

node3065.members.0.js
911
2
118964
order

281
332247
family

281
332248
genus

node3068.members.0.jsnode3068.members.1.js
281
332249
species

61
183710
family

node3070.members.0.js
61
20
1298
genus

node3071.members.0.js
1
1489678
species

node3072.members.0.js
31
68909
species

node3073.members.0.js
2
1299
species

node3074.members.0.js
4
519440
species

node3075.members.0.js
1
392413
species

node3076.members.0.js
1
1182571
species

node3077.members.0.js
1
249408
species

node3078.members.2.jsnode3078.members.3.jsnode3078.members.4.js
435855
1343837
species

node3079.members.2.jsnode3079.members.4.js
611
1256549
species

node3080.members.0.jsnode3080.members.1.jsnode3080.members.2.jsnode3080.members.3.jsnode3080.members.4.js
9082387603246
77133
species

124725
544448
phylum

node3082.members.0.jsnode3082.members.2.js
124725
22
31969
class

1083
186328
order

23
33925
family

23
46239
genus

node3086.members.0.js
2
28224
species

node3087.members.2.js
3
2151
species

106
2131
family

node3089.members.0.js
106
65
2132
genus

node3090.members.0.js
1
47834
species

node3091.members.0.js
17
2133
species

node3092.members.0.js
23
2138
species

116
2085
order

116
2092
family

116
2093
genus

node3096.members.0.js
1
1262904
species

16
656088
species group

node3098.members.2.js
16
2102
species

1374
186329
order

1374
2146
family

1374
33926
genus

1374
85620
species group

node3103.members.0.jsnode3103.members.1.jsnode3103.members.2.js
1374
35779
species

node3104.members.0.jsnode3104.members.2.js
14
12
95818
phylum

node3105.members.2.js
1
443342
species

node3106.members.2.js
1
1476577
species

12
200918
phylum

12
188708
class

12
2419
order

12
1643950
family

12
2422
genus

node3112.members.2.js
12
93466
species

31
40117
phylum

31
203693
class

31
189778
order

31
189779
family

31
1234
genus

node3118.members.0.jsnode3118.members.2.js
31
330214
species

44175221026523113112
201174
phylum

14572494
84998
class

14572494
84999
order

node3122.members.0.js
14572494
1
84107
family

node3123.members.0.jsnode3123.members.4.js
14562494
7102
102106
genus

node3124.members.0.jsnode3124.members.1.jsnode3124.members.2.jsnode3124.members.4.js
7362492
74426
species

node3125.members.0.js
10
742722
species

14
1497346
class

node3127.members.2.js
14
2
588673
order

11
320599
family

11
207599
genus

node3130.members.0.jsnode3130.members.2.js
11
363832
species

1
320583
family

1
191494
genus

node3133.members.2.js
1
191495
species

node3134.members.0.jsnode3134.members.1.jsnode3134.members.2.jsnode3134.members.3.js
29585201021223113108
5936331
1760
class

node3135.members.0.jsnode3135.members.1.jsnode3135.members.2.jsnode3135.members.3.jsnode3135.members.4.js
3063348344
3053287434
2037
order

node3136.members.2.js
16091
8
2049
family

1
184869
genus

node3138.members.2.js
1
184870
species

node3139.members.0.jsnode3139.members.2.js
16001
12
1654
genus

node3140.members.2.js
7
103621
species

node3141.members.2.js
7
131111
species

node3142.members.2.js
1
1203556
species

node3143.members.2.jsnode3143.members.3.js
5831
1660
species

node3144.members.0.jsnode3144.members.1.js
32
932042
species

node3145.members.2.js
2
723604
species

3947751
85011
order

3947751
2062
family

node3148.members.0.jsnode3148.members.2.js
3947751
1163
1883
genus

node3149.members.2.js
11
998084
species

node3150.members.2.js
4
591157
species

node3151.members.2.js
1
83656
species

node3152.members.1.js
4
285562
species

node3153.members.2.js
253
67294
species

node3154.members.2.js
15
35758
species

node3155.members.2.jsnode3155.members.3.js
41
1901
species

node3156.members.0.jsnode3156.members.2.js
38308
352211
species

node3157.members.2.js
1
645465
species

node3158.members.2.js
11
465543
species

node3159.members.2.js
1
1078086
species

node3160.members.2.js
3
591158
species

1
1643682
order

1
85030
family

1
1860
genus

node3164.members.0.js
1
1861
species

432
85008
order

432
28056
family

1
1865
genus

node3168.members.2.js
1
649831
species

31
1873
genus

node3170.members.2.js
31
47853
species

4
1121254
genus

node3172.members.0.js
4
53368
species

373400202813108232
85009
order

node3174.members.2.js
368400202812108232
3
31957
family

node3175.members.0.jsnode3175.members.1.jsnode3175.members.2.jsnode3175.members.3.jsnode3175.members.4.js
368400202512108232
269516196108037
1743
genus

node3176.members.0.jsnode3176.members.2.jsnode3176.members.4.js
111
1050843
species

node3177.members.2.js
1
1203574
species

node3178.members.2.js
37
1744
species

node3179.members.0.jsnode3179.members.1.jsnode3179.members.2.jsnode3179.members.3.jsnode3179.members.4.js
9834913676194
1747
species

node3180.members.3.js
51
1
85015
family

3
1839
genus

node3182.members.0.js
3
196162
species

1
182639
genus

node3184.members.0.js
1
460086
species

1
117156
genus

node3186.members.0.js
1
533267
species

node3187.members.0.jsnode3187.members.1.jsnode3187.members.2.jsnode3187.members.4.js
20719989314870
111181383
85007
order

node3188.members.2.js
915072134
1
1653
family

node3189.members.0.jsnode3189.members.1.jsnode3189.members.2.js
915071134
64343
1716
genus

node3190.members.0.jsnode3190.members.2.js
119
37637
species

node3191.members.2.js
6
61592
species

node3192.members.0.jsnode3192.members.2.js
13
38284
species

node3193.members.0.js
1
65058
species

node3194.members.4.js
34
161879
species

node3195.members.2.js
1
43768
species

node3196.members.0.js
1
1203624
species

node3197.members.2.js
1
1203561
species

node3198.members.2.js
1
1414719
species

node3199.members.2.js
1
38301
species

node3200.members.0.js
2
1121358
species

node3201.members.0.jsnode3201.members.2.js
21
43769
species

node3202.members.0.jsnode3202.members.1.jsnode3202.members.2.jsnode3202.members.3.js
94631
38303
species

node3203.members.1.js
1
401472
species

node3204.members.2.js
2
43770
species

node3205.members.0.jsnode3205.members.1.js
1960318033
1567
85025
family

node3206.members.0.jsnode3206.members.1.jsnode3206.members.2.jsnode3206.members.4.js
1802228033
117281453
1827
genus

node3207.members.0.jsnode3207.members.2.js
24
1828
species

node3208.members.2.js
654
1830
species

node3209.members.0.jsnode3209.members.1.js
213
1829
species

node3210.members.0.jsnode3210.members.1.js
5491
1833
species

node3211.members.0.js
1
1384060
species

node3212.members.0.js
74
334542
species

node3213.members.0.js
2
37919
species

22
1817
genus

node3215.members.1.js
2
120957
species

node3216.members.0.js
1
37330
species

node3217.members.0.js
1
373212
species

73450
1762
family

node3219.members.0.js
73450
5
1763
genus

node3220.members.4.js
3450
3280
670516
species group

170
670506
species subgroup

node3222.members.4.js
170
36809
species

node3223.members.0.js
2
1449048
species

217
85026
family

node3225.members.0.jsnode3225.members.1.js
217
21
2053
genus

node3226.members.1.js
16
83341
species

1186
85010
order

node3228.members.2.js
1186
5
2070
family

node3229.members.2.js
15
1
1847
genus

node3230.members.2.js
13
103441
species

node3231.members.2.js
1
2074
species

node3232.members.2.js
165
26
1813
genus

node3233.members.2.js
16
385957
species

node3234.members.2.js
123
330084
species

1
2029
genus

node3236.members.0.js
1
2030
species

1
2071
genus

node3238.members.2.js
1
103733
species

39216612
85004
order

39216612
31953
family

node3241.members.0.jsnode3241.members.2.jsnode3241.members.3.jsnode3241.members.4.js
39216612
39107212
1678
genus

87
246618
species

node3243.members.2.js
87
79262
subspecies

node3244.members.2.js
3
1680
species

995
216816
species

node3246.members.2.js
995
1682
subspecies

9
28025
species

node3248.members.2.js
9
302912
subspecies

node3249.members.0.js
629142
3
85006
order

13
85021
family

13
99479
genus

node3252.members.2.js
13
101689
species

node3253.members.0.js
24712
1
1268
family

3
57494
genus

node3255.members.0.js
3
515814
species

node3256.members.0.js
1851
4
32207
genus

node3257.members.0.js
14
2047
species

node3258.members.1.jsnode3258.members.3.js
51
43675
species

1
57493
genus

node3260.members.3.js
1
72000
species

node3261.members.1.jsnode3261.members.2.js
221
21
1663
genus

node3262.members.0.js
1
1494608
species

node3263.members.0.js
1
37928
species

322
85023
family

node3265.members.0.jsnode3265.members.1.js
322
302
33882
genus

node3266.members.0.js
1
199592
species

node3267.members.0.js
1
1177594
species

1
145357
family

1
57499
genus

node3270.members.0.js
1
1276
species

2
125316
family

2
84756
genus

node3273.members.0.js
2
84757
species

1
84995
class

1
84996
order

1
84997
family

1
42255
genus

node3278.members.0.js
1
42256
species

node3279.members.0.js
11
1
265317
phylum

node3280.members.2.js
1
857041
species

node3281.members.2.js
8
332979
species

node3282.members.0.jsnode3282.members.2.js
21
1522316
species

13153
203682
phylum

13152
203683
class

1
1127829
order

1
1127830
family

1
360731
genus

node3288.members.0.js
1
247490
species

12152
112
order

node3290.members.2.js
51
455066
species

5
1763524
family

5
466152
genus

node3293.members.0.js
5
466153
species

node3294.members.0.js
711
6
126
family

1
1649490
genus

node3296.members.2.js
1
119
species

1
265488
genus

node3298.members.1.js
1
1265734
species

1
600332
genus

node3300.members.0.js
1
360055
species

node3301.members.2.js
1
1298906
species

node3302.members.2.js
270
1256574
species

node3303.members.0.jsnode3303.members.1.jsnode3303.members.2.jsnode3303.members.3.jsnode3303.members.4.js
3188492237291196285
21433812543410
1239
phylum

node3304.members.0.js
1
1262989
species

node3305.members.0.jsnode3305.members.2.js
235936708464475
1325
186801
class

node3306.members.2.js
120
3
68295
order

7
227387
family

7
227388
genus

node3309.members.2.js
7
184064
species

node3310.members.2.js
9
2
186814
family

3
862261
genus

node3312.members.2.js
3
44256
species

4
140458
genus

node3314.members.2.js
4
85874
species

11
543371
family

11
291988
genus

node3317.members.0.jsnode3317.members.2.js
11
291990
species

1
485256
order

1
485255
family

1
375928
genus

node3321.members.2.js
1
375929
species

node3322.members.0.jsnode3322.members.1.jsnode3322.members.2.jsnode3322.members.3.jsnode3322.members.4.js
235736705004475
103613286801222
186802
order

177611
31984
family

177611
2697
genus

node3325.members.0.jsnode3325.members.2.jsnode3325.members.4.js
177611
35701
species

111296362
1017280
genus

node3327.members.0.jsnode3327.members.1.jsnode3327.members.2.jsnode3327.members.3.jsnode3327.members.4.js
111296362
106588
species

node3328.members.2.js
7114
6
186804
family

711
1481960
genus

node3330.members.2.js
1
1540
species

node3331.members.0.js
71
1496
species

node3332.members.2.js
6
1476973
species

1
1257
genus

node3334.members.2.js
1
1261
species

16813
186806
family

1
33951
genus

node3337.members.2.js
1
82116
species

node3338.members.0.jsnode3338.members.1.js
1588
68
1730
genus

node3339.members.2.js
2
1262894
species

node3340.members.2.js
1
1736
species

node3341.members.0.js
1
97253
species

node3342.members.0.js
1
1519438
species

node3343.members.0.js
2
457402
species

node3344.members.0.js
2
1262883
species

node3345.members.0.jsnode3345.members.2.js
34
39496
species

node3346.members.2.js
1
1392487
species

14
113286
genus

node3348.members.0.jsnode3348.members.2.js
14
113287
species

node3349.members.0.jsnode3349.members.1.jsnode3349.members.2.jsnode3349.members.4.js
68366440434
97415213
186803
family

node3350.members.0.js
13
1235800
species

110921
1506577
genus

node3352.members.0.jsnode3352.members.2.jsnode3352.members.3.js
110921
29361
species

3144773
1506553
genus

node3354.members.0.js
3
66219
species

node3355.members.2.jsnode3355.members.3.js
44773
208479
species

node3356.members.1.js
1
333367
species

4
698776
genus

node3358.members.0.js
4
29360
species

37
265975
genus

node3360.members.2.js
37
652706
species

node3361.members.0.js
530
3
189330
genus

node3362.members.0.jsnode3362.members.2.js
130
88431
species

node3363.members.0.js
1
1235798
species

39820
33042
genus

node3365.members.2.jsnode3365.members.4.js
39820
33043
species

node3366.members.0.js
5231
6
841
genus

node3367.members.0.jsnode3367.members.1.js
11
166486
species

node3368.members.0.js
1
301301
species

node3369.members.0.js
515
360807
species

1
207244
genus

node3371.members.0.js
1
105841
species

71911
43996
genus

node3373.members.0.jsnode3373.members.2.jsnode3373.members.4.js
71911
43997
species

1
830
genus

node3375.members.0.js
1
831
species

6
1766253
genus

node3377.members.0.js
6
39491
species

node3378.members.0.js
1
1410627
species

node3379.members.0.jsnode3379.members.2.js
1962
14
572511
genus

node3380.members.0.js
2
1262757
species

node3381.members.0.js
2
1262756
species

node3382.members.2.js
2
40520
species

node3383.members.0.js
13
53443
species

node3384.members.2.js
38
33038
species

node3385.members.0.js
1
1226324
species

node3386.members.2.js
18
33039
species

node3387.members.0.js
2
397291
species

node3388.members.2.js
1
397287
species

8
1392389
genus

node3390.members.0.js
8
1297617
species

node3391.members.2.js
15521098229
92
541000
family

10
35829
genus

node3393.members.2.js
10
35830
species

520922229
216851
genus

node3395.members.1.jsnode3395.members.2.jsnode3395.members.3.jsnode3395.members.4.js
520922229
853
species

node3396.members.2.js
156
13
1508657
genus

node3397.members.2.js
5
29362
species

node3398.members.2.js
14
1515
species

node3399.members.2.js
1
1499
species

node3400.members.2.js
3
288965
species

node3401.members.2.js
1
29371
species

node3402.members.2.js
3
253314
species

node3403.members.0.jsnode3403.members.2.js
116
1535
species

118
1486726
genus

node3405.members.0.jsnode3405.members.2.js
118
35825
species

node3406.members.0.js
13
3
1263
genus

node3407.members.0.js
1
657323
species

node3408.members.0.js
1
40519
species

node3409.members.0.js
5
46228
species

node3410.members.0.js
2
1262964
species

node3411.members.0.js
1
1262963
species

1
2383
genus

node3413.members.2.js
1
420336
species

152313
543314
family

1413
86331
genus

node3416.members.0.jsnode3416.members.1.js
1413
35519
species

node3417.members.0.js
11
1230734
species

13
109326
genus

node3419.members.2.js
13
109327
species

node3420.members.2.js
1
1321777
species

node3421.members.0.js
1
1232439
species

node3422.members.2.js
3534547
27
31979
family

2
1408818
genus

node3424.members.0.js
2
1408819
species

node3425.members.0.jsnode3425.members.2.js
3513367
841
1485
genus

node3426.members.0.js
1
1414720
species

node3427.members.0.js
2
97139
species

node3428.members.0.jsnode3428.members.4.js
3181
411486
species

node3429.members.0.js
1
1262845
species

node3430.members.2.js
2
1301100
species

node3431.members.0.js
1
1262807
species

node3432.members.2.js
1
1196322
species

node3433.members.4.js
6
1262796
species

node3434.members.2.js
3
641107
species

node3435.members.2.js
2
91623
species

node3436.members.0.js
1
556261
species

node3437.members.0.js
3
1262780
species

node3438.members.0.js
9
1226325
species

node3439.members.0.jsnode3439.members.2.js
319
1449050
species

node3440.members.0.js
1
1262812
species

node3441.members.2.js
1
217159
species

node3442.members.0.js
1
1262842
species

node3443.members.2.js
13
1491
species

node3444.members.2.js
2
1487921
species

node3445.members.0.js
1
1553
species

node3446.members.0.js
1
1262802
species

node3447.members.2.js
11
1262811
species

node3448.members.2.js
1
1499689
species

node3449.members.2.js
4
1534
species

node3450.members.2.js
173
29341
species

node3451.members.2.js
63
411489
species

86
114627
genus

node3453.members.2.js
1
461876
species

node3454.members.2.js
85
208226
species

5
171003
genus

node3456.members.2.js
5
116090
species

4
68298
family

4
129001
genus

node3459.members.2.js
4
86170
species

node3460.members.2.js
357
22
186807
family

node3461.members.2.js
120
12
79206
genus

node3462.members.0.js
1
1487923
species

node3463.members.2.js
2
1563
species

node3464.members.2.js
6
1531958
species

node3465.members.0.js
1
1487582
species

node3466.members.0.js
1
1531962
species

node3467.members.2.js
13
1
1562
genus

node3468.members.2.js
2
59610
species

node3469.members.2.js
1
102134
species

node3470.members.2.js
8
1564
species

node3471.members.2.js
1
42471
species

node3472.members.2.js
2
1
56112
genus

node3473.members.2.js
1
55583
species

node3474.members.0.js
1
1232445
species

node3475.members.2.js
1
1449126
species

node3476.members.0.js
1
1263004
species

node3477.members.0.jsnode3477.members.1.jsnode3477.members.2.jsnode3477.members.3.jsnode3477.members.4.js
54039385088118200
916260003981
91061
class

node3478.members.0.jsnode3478.members.1.jsnode3478.members.2.jsnode3478.members.4.js
21264366236584
4315581
186826
order

1539626
1300
family

node3480.members.0.jsnode3480.members.1.jsnode3480.members.2.js
738611
69383
1301
genus

node3481.members.2.js
4
1313
species

77
671232
species group

node3483.members.2.js
77
73
1328
species

node3484.members.2.js
4
1272910
subspecies

node3485.members.1.jsnode3485.members.2.js
129
1304
species

node3486.members.2.js
1
563038
species

node3487.members.2.js
23
1305
species

node3488.members.2.js
9
1314
species

node3489.members.2.js
1
1308
species

1
102684
species

node3491.members.2.js
1
150054
subspecies

node3492.members.0.js
1
1310
species

node3493.members.2.js
10
469609
species

node3494.members.2.js
1
1318
species

4
119603
species group

4
1336
species

node3497.members.2.js
4
40041
subspecies

node3498.members.1.jsnode3498.members.2.js
2634
1311
species

node3499.members.2.js
8
28037
species

node3500.members.2.js
1
1303
species

node3501.members.1.js
2
113107
species

node3502.members.2.js
25
1307
species

node3503.members.0.js
8115
3
1357
genus

node3504.members.0.jsnode3504.members.1.jsnode3504.members.2.js
5115
1111
1358
species

node3505.members.0.js
3
1359
subspecies

node3506.members.0.jsnode3506.members.2.js
14
1360
subspecies

151
81850
family

1
1243
genus

node3509.members.2.js
1
1403934
species

15
46255
genus

node3511.members.0.js
15
137591
species

node3512.members.0.jsnode3512.members.2.js
482311
24
186828
family

42
117563
genus

node3514.members.2.js
2
137732
species

node3515.members.0.js
4
46124
species

node3516.members.0.jsnode3516.members.2.js
23
2747
genus

382302
29393
genus

node3518.members.0.jsnode3518.members.1.jsnode3518.members.2.js
382302
29394
species

1
1651
genus

node3520.members.0.js
1
1652
species

1
633405
genus

node3522.members.0.js
1
515351
species

19114322
81852
family

node3524.members.0.jsnode3524.members.2.js
19114322
111
1350
genus

node3525.members.0.js
1
1352
species

node3526.members.0.jsnode3526.members.1.jsnode3526.members.2.jsnode3526.members.3.js
1114152
1351
species

node3527.members.0.jsnode3527.members.2.js
51
53345
species

node3528.members.2.js
15
37734
species

node3529.members.0.js
1
1354
species

node3530.members.0.js
7221336926383
9
33958
family

node3531.members.0.jsnode3531.members.1.jsnode3531.members.2.jsnode3531.members.3.jsnode3531.members.4.js
6321336926383
3117125843253
1578
genus

node3532.members.2.js
1
1624
species

node3533.members.2.js
12
1613
species

node3534.members.2.js
8
1596
species

node3535.members.2.js
59
1580
species

node3536.members.0.js
2
97137
species

node3537.members.0.jsnode3537.members.2.js
164
231049
species

node3538.members.2.js
16
47715
species

node3539.members.0.jsnode3539.members.1.jsnode3539.members.2.jsnode3539.members.3.jsnode3539.members.4.js
34203803130
47770
species

node3540.members.2.js
2
1633
species

node3541.members.2.js
18
1589
species

node3542.members.0.js
2
307126
species

node3543.members.0.js
1
1622
species

node3544.members.2.js
2
1605
species

node3545.members.0.js
8
1598
species

node3546.members.2.js
606
109790
species

3
186827
family

3
46123
genus

node3549.members.2.js
3
46125
species

node3550.members.0.jsnode3550.members.1.jsnode3550.members.2.js
319313224651435
41772055
1385
order

node3551.members.0.js
1
1460636
species

node3552.members.0.jsnode3552.members.2.js
1501731149676
16
90964
family

1
227979
genus

node3554.members.1.js
1
516700
species

node3555.members.0.jsnode3555.members.1.jsnode3555.members.2.jsnode3555.members.3.jsnode3555.members.4.js
1491721149076
1888184541
1279
genus

node3556.members.0.jsnode3556.members.1.jsnode3556.members.2.jsnode3556.members.3.js
4711221
1282
species

node3557.members.2.js
1
1234593
species

node3558.members.0.jsnode3558.members.1.jsnode3558.members.2.jsnode3558.members.4.js
116459795
115448654
1280
species

node3559.members.0.jsnode3559.members.1.jsnode3559.members.2.jsnode3559.members.4.js
111141
46170
subspecies

node3560.members.2.js
7
1403935
species

node3561.members.2.js
1
861530
species

node3562.members.2.js
11
985762
species

node3563.members.0.jsnode3563.members.2.js
135
29388
species

node3564.members.2.js
1
29380
species

node3565.members.2.js
1
28035
species

node3566.members.2.js
74
44
29385
species

node3567.members.2.js
30
147452
subspecies

node3568.members.0.jsnode3568.members.1.jsnode3568.members.2.jsnode3568.members.3.js
53173832
1157683
species

node3569.members.0.jsnode3569.members.2.js
530
1283
species

node3570.members.1.js
1
170573
species

node3571.members.2.js
156
83
186818
family

node3572.members.2.js
73
63
1372
genus

node3573.members.2.js
3
1541197
species

node3574.members.2.js
7
161360
species

41610
186820
family

1
2755
genus

node3577.members.2.js
1
2757
species

41609
1637
genus

node3579.members.2.js
30
1641
species

node3580.members.0.jsnode3580.members.1.jsnode3580.members.2.js
41579
1639
species

node3581.members.0.jsnode3581.members.2.js
14
1378
genus

node3582.members.0.js
2
1460649
species

node3583.members.0.jsnode3583.members.2.jsnode3583.members.4.js
9955189721
25591
186817
family

node3584.members.2.js
25
24
84406
genus

node3585.members.2.js
1
1462526
species

2
29331
genus

node3587.members.2.js
2
1449
species

1
182709
genus

node3589.members.2.js
1
586413
species

12
150247
genus

node3591.members.0.jsnode3591.members.2.js
12
1535751
species

node3592.members.0.jsnode3592.members.2.js
612
111
129337
genus

node3593.members.2.js
1
81408
species

node3594.members.0.js
5
471223
species

61
400634
genus

node3596.members.1.js
5
1421
species

node3597.members.2.js
1
561440
species

node3598.members.1.js
1
28031
species

node3599.members.0.jsnode3599.members.1.jsnode3599.members.2.jsnode3599.members.3.js
674917942
162111041
1386
genus

node3600.members.2.js
14
313627
species

node3601.members.2.js
1
1131816
species

node3602.members.1.jsnode3602.members.2.js
2625148
226
86661
species group

node3603.members.2.js
1
574376
species

node3604.members.0.jsnode3604.members.1.jsnode3604.members.2.js
262137
1396
species

node3605.members.2.js
4
1392
species

node3606.members.1.js
1
1428
species

node3607.members.3.js
1
1478
species

node3608.members.2.js
21
1413
species

node3609.members.2.js
36
935837
species

node3610.members.2.js
16196
1
653685
species group

node3611.members.2.js
135
1390
species

node3612.members.0.jsnode3612.members.2.js
1659
1402
species

node3613.members.2.js
1
1423
species

node3614.members.0.js
3
1399
species

node3615.members.2.js
10
1408
species

node3616.members.2.js
211
220686
species

node3617.members.2.js
1
1132442
species

node3618.members.2.js
1
1418
species

node3619.members.2.js
1
1460641
species

node3620.members.0.jsnode3620.members.2.js
110
561879
species

node3621.members.0.js
2
1347086
species

node3622.members.2.js
22
1287657
species

node3623.members.2.js
1
1033734
species

node3624.members.2.js
2
1408303
species

node3625.members.0.js
1
384933
species

node3626.members.0.js
1
659243
species

node3627.members.2.js
4
363872
species

node3628.members.2.js
1
1298866
species

node3629.members.2.js
1
1423321
species

node3630.members.2.js
1
1340434
species

node3631.members.1.js
3
1404
species

node3632.members.2.js
8
293386
species

node3633.members.0.js
1
254759
species

1
74385
genus

node3635.members.2.js
1
307521
species

node3636.members.2.js
1676247528
1
186822
family

1
456492
genus

node3638.members.0.js
1
456493
species

node3639.members.0.jsnode3639.members.1.jsnode3639.members.2.jsnode3639.members.3.js
1576245528
6641
44249
genus

node3640.members.1.js
1
1395586
species

node3641.members.2.js
10
996640
species

node3642.members.2.js
1
1536769
species

node3643.members.2.js
1
1536772
species

node3644.members.2.js
3
78057
species

node3645.members.0.js
5
1380372
species

node3646.members.0.js
1
1449063
species

node3647.members.2.js
2
1333845
species

node3648.members.0.js
2
1236974
species

node3649.members.0.js
1
1243662
species

node3650.members.2.jsnode3650.members.3.jsnode3650.members.4.js
6224428
483908
species

1
55079
genus

node3652.members.2.js
1
278991
species

node3653.members.0.js
5
1460635
species

node3654.members.0.js
1
1263021
species

551956
909932
class

node3656.members.0.js
551956
2
909929
order

node3657.members.0.jsnode3657.members.2.js
531956
333
31977
family

81
261684
genus

node3659.members.0.jsnode3659.members.2.js
81
261685
species

node3660.members.0.js
125
1
29465
genus

node3661.members.2.js
25
1403932
species

1
52225
genus

node3663.members.2.js
1
1321780
species

node3664.members.0.jsnode3664.members.2.js
62
21
365348
genus

node3665.members.0.jsnode3665.members.2.js
21
484770
species

node3666.members.0.js
2
365349
species

1
2373
genus

node3668.members.0.js
1
2374
species

23
82202
genus

node3670.members.2.js
23
82203
species

11
2375
genus

node3672.members.0.jsnode3672.members.2.js
11
2378
species

319
970
genus

node3674.members.0.js
3
1408313
species

node3675.members.1.js
19
135080
species

node3676.members.0.js
2
1263009
species

151117
1737404
class

151117
1737405
order

node3679.members.0.jsnode3679.members.1.js
151117
11
1570339
family

node3680.members.1.js
11016
3
165779
genus

node3681.members.0.jsnode3681.members.1.js
17
33034
species

node3682.members.2.js
16
33029
species

node3683.members.0.js
111
5
162289
genus

node3684.members.0.js
5
875453
species

node3685.members.0.js
1
1465756
species

node3686.members.2.js
1
1175452
species

2
150022
genus

node3688.members.0.js
2
1260
species

30
526524
class

30
526525
order

node3691.members.2.js
30
1
128827
family

node3692.members.2.js
3
1262981
species

node3693.members.2.js
26
457422
species

node3694.members.0.jsnode3694.members.2.jsnode3694.members.3.jsnode3694.members.4.js
72573422
795322
species

node3695.members.0.js
821
3
200795
phylum

22
388447
class

22
388448
order

22
388449
family

22
363276
genus

node3700.members.0.jsnode3700.members.1.js
22
363277
species

2
32061
class

2
189772
order

2
189773
family

2
64
genus

node3705.members.0.js
2
65
species

node3706.members.2.js
1
710736
species

1
189775
class

1
1649508
genus

node3709.members.0.js
1
1382356
species

node3710.members.0.js
1
1047063
species

node3711.members.2.js
40
1343846
species

node3712.members.0.jsnode3712.members.1.jsnode3712.members.2.js
116033506300936308
14312
68336
superphylum

node3713.members.0.jsnode3713.members.1.jsnode3713.members.2.jsnode3713.members.3.jsnode3713.members.4.js
115863503299636308
19791147772109
976
phylum

node3714.members.0.js
3
100480
species

146117457361816
117747
class

node3716.members.0.jsnode3716.members.1.jsnode3716.members.2.jsnode3716.members.4.js
146117457361816
3831592
200666
order

node3717.members.0.jsnode3717.members.1.jsnode3717.members.2.jsnode3717.members.3.js
2731687381811
411008265
84566
family

node3718.members.0.jsnode3718.members.1.js
36163927
969
84567
genus

node3719.members.0.jsnode3719.members.1.js
112
509635
species

node3720.members.0.jsnode3720.members.1.jsnode3720.members.2.jsnode3720.members.3.js
3611
1122941
species

node3721.members.0.jsnode3721.members.2.js
51
1122943
species

node3722.members.0.jsnode3722.members.1.jsnode3722.members.4.js
2217
984
species

node3723.members.0.jsnode3723.members.1.jsnode3723.members.2.js
381
454586
species

node3724.members.0.jsnode3724.members.1.js
13
475254
species

node3725.members.0.jsnode3725.members.1.jsnode3725.members.3.js
3151
752140
species

node3726.members.1.jsnode3726.members.2.js
131
1069985
species

node3727.members.0.jsnode3727.members.1.js
49
34086
species

node3728.members.1.js
2
391596
species

node3729.members.0.jsnode3729.members.1.jsnode3729.members.2.js
314
1503925
species

node3730.members.0.jsnode3730.members.1.jsnode3730.members.2.js
241
1270196
species

1
929509
genus

node3732.members.0.js
1
995
species

61
1288026
genus

node3734.members.0.jsnode3734.members.2.js
61
1288027
species

3
376469
genus

node3736.members.1.js
3
376470
species

node3737.members.0.jsnode3737.members.1.js
185508104
87
28453
genus

node3738.members.1.js
15
1408813
species

node3739.members.0.js
1
403776
species

node3740.members.0.jsnode3740.members.1.jsnode3740.members.3.js
311
743722
species

node3741.members.0.jsnode3741.members.1.js
118
258
species

node3742.members.1.js
3
1590596
species

node3743.members.0.jsnode3743.members.1.jsnode3743.members.3.jsnode3743.members.4.js
16247494
259
species

121
1649482
genus

node3745.members.0.jsnode3745.members.1.jsnode3745.members.2.js
121
151895
species

3311
423349
genus

node3747.members.0.jsnode3747.members.1.jsnode3747.members.2.jsnode3747.members.3.js
3311
423351
species

node3748.members.0.jsnode3748.members.1.jsnode3748.members.2.js
1146256373
27915432
563835
family

node3749.members.2.js
1
1094955
species

63338
460073
genus

node3751.members.0.jsnode3751.members.1.jsnode3751.members.2.js
63338
398037
species

13321
79328
genus

node3753.members.0.js
2
339340
species

node3754.members.0.jsnode3754.members.2.js
21
1235985
species

node3755.members.0.jsnode3755.members.1.jsnode3755.members.2.jsnode3755.members.4.js
9311
79329
species

2036
354354
genus

node3757.members.0.jsnode3757.members.2.js
2036
354356
species

node3758.members.0.jsnode3758.members.2.js
735
21
379899
genus

node3759.members.0.jsnode3759.members.2.js
12
446683
species

node3760.members.0.jsnode3760.members.1.jsnode3760.members.2.js
432
379900
species

3529
296051
genus

node3762.members.0.jsnode3762.members.2.js
3529
249
species

node3763.members.0.jsnode3763.members.2.jsnode3763.members.4.js
687552
616392
504481
genus

node3764.members.2.js
10
1267211
species

node3765.members.0.jsnode3765.members.2.js
676
925409
species

node3766.members.0.js
1
426421
species

node3767.members.0.js
3
1076522
species

node3768.members.0.jsnode3768.members.2.js
25
1268323
species

node3769.members.0.jsnode3769.members.2.js
40134
1212
1004301
genus

node3770.members.0.jsnode3770.members.1.jsnode3770.members.2.js
21115
1463156
species

node3771.members.0.jsnode3771.members.2.js
77
1349421
species

422
89374
family

322
2349
genus

node3774.members.0.jsnode3774.members.1.jsnode3774.members.2.js
322
2350
species

1
70994
genus

node3776.members.0.js
1
70995
species

21
1100070
order

21
28194
genus

node3779.members.0.jsnode3779.members.1.js
21
55505
species

210620952226
200643
class

node3781.members.0.jsnode3781.members.1.jsnode3781.members.2.jsnode3781.members.4.js
210620952226
3983641
171549
order

3
1471398
family

2
1471399
genus

node3784.members.0.js
2
1168034
species

1
314318
genus

node3786.members.0.js
1
314319
species

21
171550
family

1
1611681
genus

node3789.members.2.js
1
1433126
species

node3790.members.0.js
2
1
239759
genus

node3791.members.0.js
1
1288121
species

node3792.members.0.js
10
1400053
species

node3793.members.0.js
22312
1
171551
family

node3794.members.2.js
2
836
genus

node3795.members.1.jsnode3795.members.2.js
37
13
156973
genus

node3796.members.2.js
4
163665
species

node3797.members.1.js
2
45254
species

13
195950
genus

node3799.members.0.js
1
1262978
species

node3800.members.2.js
3
28112
species

node3801.members.0.js
1
1517682
species

1
574697
genus

node3803.members.0.js
1
544644
species

node3804.members.0.js
12
4
375288
genus

node3805.members.0.js
3
328812
species

node3806.members.0.js
3
574930
species

node3807.members.0.js
2
46503
species

node3808.members.0.js
6
283168
genus

node3809.members.0.js
166014122224
2
815
family

node3810.members.0.jsnode3810.members.1.jsnode3810.members.2.jsnode3810.members.3.jsnode3810.members.4.js
165814122224
12774122
816
genus

node3811.members.0.jsnode3811.members.2.jsnode3811.members.4.js
181221
820
species

node3812.members.0.js
4
1078089
species

node3813.members.0.js
1
556259
species

node3814.members.2.js
1
392838
species

node3815.members.0.js
4
28116
species

node3816.members.0.js
1
469590
species

node3817.members.0.js
3
732242
species

node3818.members.0.jsnode3818.members.1.jsnode3818.members.2.jsnode3818.members.4.js
223951
817
species

node3819.members.1.js
1
457390
species

node3820.members.0.js
18
246787
species

node3821.members.0.js
9
1262753
species

node3822.members.0.js
1
371601
species

node3823.members.0.jsnode3823.members.2.js
21
47678
species

node3824.members.0.js
9
469593
species

node3825.members.0.js
21
357276
species

node3826.members.0.js
1
338188
species

node3827.members.2.js
3
469589
species

node3828.members.0.js
24
204516
species

node3829.members.0.js
8
310298
species

node3830.members.0.js
1
457387
species

node3831.members.0.js
33
821
species

1161
171552
family

511
1283313
genus

node3834.members.0.jsnode3834.members.2.jsnode3834.members.4.js
511
712469
species

1
577309
genus

node3836.members.2.js
1
454154
species

node3837.members.0.jsnode3837.members.2.js
64
34
838
genus

node3838.members.0.js
1
386414
species

node3839.members.0.js
2
1200547
species

node3840.members.0.jsnode3840.members.1.jsnode3840.members.2.js
1716553122653
1011
117743
class

node3841.members.0.jsnode3841.members.1.jsnode3841.members.2.js
1706552122553
32522
200644
order

1
1333713
family

1
1333716
genus

node3844.members.1.js
1
884107
species

node3845.members.0.jsnode3845.members.1.jsnode3845.members.2.jsnode3845.members.3.js
1673546120353
2992646744
49546
family

node3846.members.1.js
1
59734
genus

91
76831
genus

node3848.members.0.jsnode3848.members.2.js
91
76832
species

node3849.members.2.js
104
1
34084
genus

node3850.members.2.js
102
34085
species

node3851.members.2.js
1
103810
species

1
104267
genus

node3853.members.0.js
1
107401
species

node3854.members.0.jsnode3854.members.1.jsnode3854.members.2.jsnode3854.members.4.js
128431343
11521983
237
genus

node3855.members.0.jsnode3855.members.1.js
11
329186
species

node3856.members.0.jsnode3856.members.1.js
13
657326
species

node3857.members.2.js
3
350894
species

node3858.members.1.jsnode3858.members.2.js
46
95618
species

node3859.members.0.jsnode3859.members.1.js
61
344881
species

node3860.members.0.js
1
991
species

node3861.members.0.js
3
426226
species

node3862.members.0.js
4
29536
species

node3863.members.0.js
1
1202532
species

node3864.members.0.js
1
498301
species

node3865.members.2.js
1
996
species

node3866.members.0.js
3
1506583
species

node3867.members.0.jsnode3867.members.2.js
11
55197
species

node3868.members.0.jsnode3868.members.2.js
811
986
species

node3869.members.0.js
1
150172
species

node3870.members.0.js
3
554283
species

node3871.members.0.js
2
503361
species

node3872.members.0.js
3
1229487
species

node3873.members.0.js
2
1298868
species

node3874.members.2.js
1
510946
species

node3875.members.0.jsnode3875.members.1.js
21
350893
species

node3876.members.1.js
2
1341165
species

node3877.members.0.js
1
1566023
species

node3878.members.2.js
13
206041
species

node3879.members.0.js
6
1131812
species

node3880.members.0.js
4
245
species

node3881.members.0.js
5
1144313
species

node3882.members.0.jsnode3882.members.1.js
11
336276
genus

node3883.members.2.js
10
531844
species

node3884.members.0.jsnode3884.members.1.jsnode3884.members.2.jsnode3884.members.3.js
24222681
8131401
59732
genus

node3885.members.1.js
1
412438
species

node3886.members.1.js
3
1500286
species

node3887.members.2.js
7
421525
species

node3888.members.1.js
29
311334
species

node3889.members.0.jsnode3889.members.1.jsnode3889.members.2.js
126
250
species

node3890.members.0.js
1
445961
species

node3891.members.0.jsnode3891.members.1.jsnode3891.members.2.js
242
307480
species

node3892.members.2.js
2
266749
species

node3893.members.1.js
18
1144316
species

node3894.members.2.js
1
266748
species

node3895.members.0.jsnode3895.members.1.js
31
421058
species

node3896.members.0.jsnode3896.members.2.js
12
493376
species

node3897.members.0.jsnode3897.members.1.js
120
1453492
species

node3898.members.0.jsnode3898.members.1.jsnode3898.members.2.js
271
365343
species

node3899.members.1.js
1
456299
species

node3900.members.2.js
1
510955
species

node3901.members.0.jsnode3901.members.2.js
14
1233950
species

node3902.members.0.jsnode3902.members.1.jsnode3902.members.2.js
331
236814
species

node3903.members.1.js
1
253
species

node3904.members.1.js
1
558152
species

node3905.members.0.jsnode3905.members.2.js
11
1500282
species

1
1476424
genus

node3907.members.0.js
1
1178825
species

3128
1016
genus

node3909.members.1.js
1
706435
species

node3910.members.0.jsnode3910.members.2.js
315
28188
species

node3911.members.2.js
13
1017
species

3
244698
genus

node3913.members.0.js
1
1167637
species

node3914.members.0.js
1
985255
species

node3915.members.0.js
1
1283286
species

9
252356
genus

node3917.members.0.js
9
313603
species

1
28250
genus

node3919.members.2.js
1
28251
species

node3920.members.1.js
1
1042376
species

48
59735
genus

node3922.members.1.jsnode3922.members.2.js
48
1015
species

1
83612
genus

node3924.members.0.js
1
57029
species

211
290174
genus

node3926.members.2.js
1
1443666
species

node3927.members.1.js
1
1317122
species

node3928.members.0.js
1
1296415
species

node3929.members.0.js
1
279356
species

5
178469
genus

node3931.members.0.js
5
616991
species

node3932.members.1.js
4
1
112040
genus

node3933.members.1.js
3
63186
species

11
221065
genus

node3935.members.0.jsnode3935.members.2.js
11
221066
species

2
444051
genus

node3937.members.2.js
2
393060
species

node3938.members.0.jsnode3938.members.1.jsnode3938.members.2.js
2611265
49236
308865
genus

node3939.members.2.js
10
238
species

node3940.members.0.jsnode3940.members.1.jsnode3940.members.2.js
22114
1117645
species

node3941.members.1.jsnode3941.members.2.js
15
172045
species

node3942.members.1.jsnode3942.members.2.js
256
31
358033
genus

node3943.members.0.jsnode3943.members.1.jsnode3943.members.2.js
215
191577
species

node3944.members.1.js
1
421072
species

1
111500
genus

node3946.members.0.js
1
1250232
species

1
379072
genus

node3948.members.0.js
1
1392490
species

1
246874
family

1
332102
genus

node3951.members.0.js
1
191579
species

node3952.members.0.js
431937167154
2
768503
class

node3953.members.0.jsnode3953.members.1.jsnode3953.members.2.js
431737167154
8751
768507
order

node3954.members.0.jsnode3954.members.1.jsnode3954.members.2.jsnode3954.members.4.js
40062875110
848172
89373
family

39812
861913
genus

node3956.members.0.jsnode3956.members.1.jsnode3956.members.4.js
39812
663275
species

node3957.members.0.jsnode3957.members.2.js
981221
521
89966
genus

node3958.members.0.jsnode3958.members.1.jsnode3958.members.2.js
1321
223903
species

node3959.members.0.jsnode3959.members.4.js
7021
1356852
species

node3960.members.0.js
9
1446467
species

node3961.members.0.js
1
1385664
species

node3962.members.0.js
2
119644
species

node3963.members.0.js
202
1505605
species

22
1023
genus

node3965.members.0.jsnode3965.members.2.js
22
1027
species

3
312278
genus

node3967.members.0.js
3
312279
species

node3968.members.0.js
3
1094958
species

101
861914
genus

node3970.members.0.js
101
651143
species

711
101
genus

node3972.members.0.jsnode3972.members.1.jsnode3972.members.2.js
711
103
species

121
1011
genus

node3974.members.0.jsnode3974.members.2.js
121
153721
species

87
299566
genus

node3976.members.0.jsnode3976.members.1.js
87
299567
species

9
451373
genus

node3978.members.0.js
9
451374
species

1
336827
genus

node3980.members.0.js
1
336828
species

node3981.members.0.js
7
5
978
genus

node3982.members.0.js
1
29530
species

node3983.members.0.js
1
985
species

1
1494913
genus

node3985.members.0.js
1
1048983
species

node3986.members.0.jsnode3986.members.1.jsnode3986.members.2.jsnode3986.members.4.js
2612911
12651
120831
genus

node3987.members.0.jsnode3987.members.1.jsnode3987.members.2.jsnode3987.members.3.js
1531
365489
species

node3988.members.0.js
8
492736
species

node3989.members.1.js
1
1211851
species

node3990.members.0.js
1
292407
species

node3991.members.0.jsnode3991.members.2.js
41
94254
species

node3992.members.0.jsnode3992.members.2.js
12247
36
323449
genus

node3993.members.0.jsnode3993.members.1.jsnode3993.members.2.js
511
336989
species

node3994.members.0.jsnode3994.members.1.jsnode3994.members.2.js
419
1144253
species

node3995.members.2.js
31
323450
species

node3996.members.0.jsnode3996.members.2.jsnode3996.members.4.js
1562144
44121
107
genus

node3997.members.0.js
60
431554
species

node3998.members.0.jsnode3998.members.2.jsnode3998.members.4.js
69912
496058
species

node3999.members.0.jsnode3999.members.1.jsnode3999.members.4.js
16411
108
species

node4000.members.0.jsnode4000.members.2.js
1981
431553
species

node4001.members.0.js
222
8
105
genus

node4002.members.0.jsnode4002.members.2.js
41
94255
species

node4003.members.0.js
7
106
species

node4004.members.0.jsnode4004.members.2.js
31
370978
species

31
992
genus

node4006.members.1.js
1
999
species

node4007.members.0.js
1
1002
species

node4008.members.0.js
2
997
species

node4009.members.0.js
202344
4
563798
family

144
68288
genus

node4011.members.4.js
44
104
species

node4012.members.1.js
1
1350429
species

node4013.members.0.js
11
1288963
species

2
1245590
genus

node4015.members.0.js
2
1245591
species

213
246875
genus

node4017.members.0.jsnode4017.members.2.js
12
264027
species

node4018.members.0.js
1
226504
species

node4019.members.1.jsnode4019.members.2.js
11
344884
species

1
1187078
genus

node4021.members.0.js
1
645110
species

183
1501348
family

node4023.members.0.js
72
59
273135
genus

node4024.members.0.js
11
672794
species

node4025.members.0.js
2
249402
species

111
281119
genus

node4027.members.0.js
111
281120
species

node4028.members.0.js
21288
4
200667
family

1
869806
genus

node4030.members.0.js
1
1006
species

3
70992
genus

node4032.members.0.js
3
70993
species

21
1133570
genus

node4034.members.0.jsnode4034.members.1.js
21
649507
species

88
59739
genus

node4036.members.2.js
88
367791
species

6
1265689
genus

node4038.members.0.js
6
1265690
species

51
396811
genus

node4040.members.0.jsnode4040.members.1.js
51
881893
species

31
1090
phylum

31
191410
class

31
191411
order

31
191412
family

1
256319
genus

node4046.members.2.js
1
1097
species

3
1091
genus

node4048.members.0.js
2
1096
species

node4049.members.0.js
1
337090
species

node4050.members.0.jsnode4050.members.2.jsnode4050.members.4.js
9394311
731
10239
superkingdom

23
11632
family

node4052.members.0.jsnode4052.members.2.js
12
126994
species

node4053.members.2.js
1
11827
species

node4054.members.0.js
1
87786
species

1
186534
family

1
187214
genus

node4057.members.0.js
1
172220
species

node4058.members.2.js
229231
6
28883
order

node4059.members.1.js
12
1
10662
family

node4060.members.2.js
1
1187128
species

node4061.members.2.js
1
1560342
species

node4062.members.0.jsnode4062.members.2.jsnode4062.members.3.js
22141
18111
10699
family

node4063.members.2.js
1
1176422
species

node4064.members.0.js
1
1556290
species

node4065.members.0.js
1
1498188
species

node4066.members.0.js
1
376758
species

node4067.members.2.js
1
1229790
species

node4068.members.2.js
1
1173759
species

node4069.members.0.js
1
1229782
species

81
10744
family

node4071.members.2.js
1
1481187
species

8
542835
subfamily

8
477967
genus

node4074.members.1.js
8
718008
species

node4075.members.0.js
5
1211417
species

node4076.members.0.js
43
28
151341
family

node4077.members.0.js
15
12
10624
genus

node4078.members.0.js
1
1606497
species

node4079.members.0.js
1
1236391
species

node4080.members.0.js
1
10627
species

node4081.members.2.js
14
11
11050
family

3
11102
genus

node4083.members.2.js
3
11103
species

13
10482
family

12
10485
genus

node4086.members.0.js
3
39640
species

node4087.members.0.js
9
452647
species

1
10483
genus

node4089.members.0.js
1
419435
species

9342
2157
superkingdom

node4091.members.1.js
53
2
651137
phylum

node4092.members.1.js
1
1407055
species

5
1643678
class

5
1033996
order

5
1033997
family

node4096.members.0.js
5
4
497726
genus

node4097.members.0.js
1
497727
species

2
28889
phylum

node4099.members.0.js
1
1130302
species

1
183924
class

1
2281
order

1
118883
family

1
41980
genus

node4104.members.0.js
1
1111107
species

242
28890
phylum

123
224756
class

21
570264
order

21
570265
family

21
570266
genus

node4110.members.2.js
21
570267
species

12
94695
order

12
1392996
family

12
1392997
genus

node4114.members.0.jsnode4114.members.2.js
12
1392998
species

19
183925
class

19
2158
order

19
2159
family

19
2160
genus

node4119.members.2.js
19
2162
species

node4120.members.0.js
1
913322
species

node4121.members.0.js
2
155900
species

node4122.members.0.js
6
511658
species
