# Supplementary material for: Functional Metagenomics of Spacecraft Assembly Cleanrooms: Presence of Virulence Factors Associated with Human Pathogens
Source: Front Microbiol. 2016 Sep 9;7:1321. doi: 10.3389/fmicb.2016.01321 (PMC5017214; doi:10.3389/fmicb.2016.01321)
Supplement: Supplementary file 3 [file Presentation2.ZIP › Supplement.SI2.kegg.html]

Javascript must be enabled to view this page.

members
magnitude
magnitudeUnassigned

Phoenix before assembly
Phoenix during assembly
Phoenix after assembly
DAWN during assembly
MSL during assembly

1336024916298350557664699

1336024916298350557664699

124829251014760343037

331799236944343036

3428641

1

1

1

12227

57

21

212

19241

1045

3231

1

12

131

121611

2

1

91611

798259351

445

1

48

1

2431111

97

2

211

11

315

61022

16

11524054

512

1

6494

3

7727225335343034

21

1

4427

1231

4666

211114029343003

1

1

24214

7416

937121

348

16185530

31

1

3

1613

613

7223710632

3710159

3

411421

152041

125

3

798769

215471

541782041

5

1

1

2914

2

31

4678

1

1

523441

19

291320

1

5

412628

532125466049

2781

2

1

62

1

87

11

1

2

1

1

3

2

2

2

1

1

1622288

2

1

2

1

11

2

8

1

3

13

1

3

1

12

1

2

4

2

105

1

3

1

1

5

1420

3

1

11

1

1

1

2

1

51

1356

1

14752353626

1

1

1

2

1219

31

813

1847

1

1

2517

25

513

305

12351

11224

514

286

24

1

38

26

14

2112

51113

1246103

425

3112

162

1

1

77

3

924

25

2481

21113

19221

169

1910

310

1113

11415

1

11110

512

1

3313

19

337

1156

923

3919

1

6

2209

1813

198

55

1115

1

4

26

12

2412

7542

1794

216

1414

1

11917

101

1517

361467

116

1

2

2

13

213

1

1

21121

1

5

3

3

1

2

712

13

1

1

16069510063

67145

17511

14

71881

11830

7782

71019

33538

7328

422951

2278531

277571

196

167222

13

1115

2

2144

233622

3836

219

3131

1

2

53601

4633

5993

8275

145

21

3

2

1

11

1

1

1

2

1

2

6870

2

1

1

1

1

2

2

1

1

1

1

11

6

13

1

7

2

1

4

2

1

2

1

2

1

1

2

1

52

1

2

4

1

111

1

1

1

303872109971

6

1

4

1

2029

1

1

1

1

1

2

1

9

1

5

15

2

1

7

1

6545

2

17

1

1

1

1

1

10

16

2

1

1

4

2

1

7

2

1

8

1

3

2

4

1

11

1

11

1

1

31

803665265

4

82519

212

1

3

4

14

1812

1

1523

1

3

41031

1

4928

4

1

1

397

1

2

538441

11

2269

131471

118

2246113

614

1069145

21

2

39

15361

1

8162

1811

41144731

1

1

1

1

2

1

1

1

11

1

1

3

1

1

12

21

2

5

49127

1

4

2

21

1

1

2

1

495

1

1

1

3

2

2

812932812

162

3236

8442

217536

510

25

3618

2

26

1

212321

141091

840541

16115

29

11

138

119

1

16749419061

91580

2

2

3

1

1

1

33

2

31

3345

1345

1

1

8322

1

7122

1

10

310

10

10

3

1

2

4

3

3

1

1

8

3

2

1

1

1

1

1

2

2

1

1

1947876

84786

2

24786

4

2

2

1

1

3

2

1

27

17

1

3

3

102

92

2

9

1

1

121111

1

1

1

1

112

2

1

1

28

8

2

2

2

1

1

6

5

1

5979

1

1

3979

3979

1

1

1511

141

141

11

1

1

3061136138518119559

9917242

2829

1

4

3

1

12

1

1

4

23

3

1

1

1

13

3

1

1

1

31129

184

7

2

1

633

2

612

5

1

1113

4

4

13

3

74

1

1

3

1

1

1

1

1

1

221767

9

6

1

4

1

2

2

5

11756

2

136108135812

59106035212

73316

27

366

53119

36

12

91126

20

4918310

110

1713

202018

10

15

410

1223612

91

3

1

1

3

1

1

5014

8

1

1

2

1

1

1

2

11

3

3

30

1

1

1319

4

9

19

11

11

4

2

2

18385119404

2

2

7

1

4

1

1

9375119404

1

3

5375119404

1

1

53357771155

12

1

1

1

1

2

1

1

2

1

1

4135663155

8123

4

178

6

26

687

11

2537

120117

19

21155

15

37

31

11

2

28

7

17123

150

115

4

1121

1141

12

28

161

40

18

1885328017178508118

1089264113285368113

97715919396288107

3121

2

1

1

1

6671

5

3

3

11916

1

1

1110

1221

4188

8

1524

11771

57

7196615

1

1

139282

248

22385

1

32491

11

165181

5

291617152

2550

7

1529

11

37816129

181617

73787812

9

3

37

12

4

1

109181

5111211

15

1331

2

191

166

1119

8

110391

1323

4

1391

1

6

92

2

239

4

1

16114

7

1

3

434391

144

24515

213204

2

27

7

40

4

296

111224639

125212510

7321

8

1

15

1

25

1

16

562

3

11

61

31537

31523

60128

3291

1

3245

48

1

5151

2633

161

9

2

6

66908

13026

24

1

25

32284943

3

12

1

42

8

220120

1228

1

3

1

135

12

13

1

1510

12129872

64

137

639

148

1

20810

4198592

173

134

26602

1

922

1

5442

102343377946

5214

2130

28

14662

35

156

125

817491

33

931

143

58191

941

1191222

8130

2181

1252

2

12576

15

1441

4

575

129

4515

1

253

44521

1

107071104

3

1

1781

31

36874

15

29

31

1

13016

73

1

3

2

1

1

1

1

3

1

2

12013

1

11

1

1

1

1

3

1

2

1

1

1

36

1

1

70

6666393877145

1

1

771

4

1

1

45

11

1

3

1

1

1

1

1

2

2

1

3

3

3

2

1

1

611

1

2

1

1

11

1

2571012

1

250102

1

2

1

3

161

1

2

1

2

1

1

1

1

2

3

1

1

1

1

6

1

1

3

1

292

1

2

3

6

4

1

2

8

1

1

1

1

2376273866141

2

3725

1

416

5146

1328

2

736

2

3512551

1

15171

1

34

39

18

2

211

350

3

127

4247711

1

1

11

3419

24

6

31

16181

2

30

1333412

1

33

5

1

107

467495

1

5

19

2947

7

3

24

1

1

9

5

7

910

18

5

246

21011

5

1

131

18

8

4

647

32287

38

44250

21

1

1022

1

2

4101

111131

21319

2

1

112

65

319541

1

1

311

27

11

2

518

313

21

1

1

35

34

21741

2

19

1

216

11

2248

9

52232

8

51

163

289

17

1

1

1

3113

2

21

1

2

1

3

729

216

9

2

21951

16

44131

62

1

141

1

29

10

1

1

4

2

2

5

4

1

2111

3

3

3

1

1

2

1

1

3

1

1

2

1

3

1

2

2

1

1

74130910783

423098281

91

21

3

4

1

1

61

61

17

7

1

421221

321221

1

221512

2111

1411

1

329361

329361

123751

23151

16

1

1

2

2

3

2

1

161

1

1

16

16

1441

1

1

144

1

143

4

4

1

3

854

1

1

9

5

2

2

1

1

3

3

723

1

673

4

450192

5

1

1

1

1

1

1921

2

94

911

1

1

3

253191

31

1

1

106

1

4

133

1

814

901316772269343415193981

73561

13

13

12

2

1

344

25

94

5152

129

3123

1

1745397957

172312173

8178

1

34

15

15

11

2

1017

16

841

24

331

2114

16472

1112

19

263

27

131

5

1

1

3

1

3

2346

2

8

1

6

24

622

411201

8

2

111

6

4146

154

1

1

2

1

2

3

1

1

16210

2

1

1

4

3

10

7

413013554

11

2017

280131

5

47

5731812

27

2116

817

93821

2352

21311

91

159

122

1

5

5

14

12

2

6

5

1

2

1

1

139505146531

52

44

5

3

2934185

243

5182

295

93952

93946

6

642282

1108

1516

17

21

9

3

52

362

221

1

2

114

839

839

1

1

3616391

3511

1637

11

1

1

5150

4

191

455

42

5

37

466

466

350101

2

150

26

1

74

503123833

1

4

23

1

2

4

6

6

6

2633

24

39

9

3

11

911

6

1

34

60101

6

5575

222

1821

615421

29

15

345

1844

2

3

59

9

27

2

1020

13146

511

6

6

67

67

12812741901042201114

3483189377209240

12306

2265188913209240

815142

3116

428

3

1

28

3373

3373

1

1

6

2

3

1

52139

23

28

2108

3

151

4

18

10

3

2

6

18

523902363376

27551

1145376

1

10

3

9131

1

4244192

1

111

1

16

1320

2

1627

113438

1

16221

1

21

20266155

1262

71353

1

5

2

8

2

1

211

1

34952553498

8

724

1521498

24771443

2

334

12

1

12

20410341785131

16

6

1

4

4

1656135

222

1

19

7

36

3

116

2

4813

1

223

1

675

1

2

2

116055

34

158

17

322

5

62221

1687

34

1

2

8

25

1

1

2

1

1

161798

161798

401423061

41433

51

868

3

1

1

51511

6

4949

617

1

11

3320

1

1

48

2

1

14475

1

3626

1

2

2

2

2

392914724

28

1338144

1100332

65

24746

23

591

421

41

481611

1544

4

123

42

5

1

2

1

235

35

2

197513611

2

1

1812011

7514

72

3

12

1

2

224401

12411

39

1

213205276

293

818

12371

4438

36281

13

2

111913334

24

148

1172

197

82516322056139

741896155598

4

4216

2

83471

61516

4

1822531

3

149

1

1

10733

4

30

2

103

1051

1851

1

30556231

2

70

314547

11

5518

61915

1

6

2

10

71

73

118

743

6

51225

5

15

810

117

871

1132

1

1647254

2139

39212

8949

1

3621

4

12

416

1

7686

1

2

112

3529212

522491

117

3

114

831

152118

214

1

1114

8

1

1217

1

7

13839

4

2

1510

172340

2

27

4

11

4

8431

5321

413

422

1

17

2

431426

338

316

16

1

1

228

1

3320

11

3

1

8473650141

70

17102

48

324

12

41

12

1

114

9

2

113184

1

11

1

203259

85

1

24

33313

11

53099

5

6

1914

1

3

817

8

1

114

8116

2

1

11322

139

33488441

632210823002121

21104051612

23

36087

227

21

6431

3421214

329

421

1

134

1

1

13322443421

19

31

2237

25

6

22

6

1

2

52291

1

1

4

24

23

11

2

43

4

1

315

135

2

6

8

11

942

335

2524

4

611

291327

29

4

1411491

2

3

2

1

613

101275

23

4

7

4

1

16421

3641

711

26

92422

32312

1

619

19881951

83

31849

42011

122371

1

3

730

556

12

426

112561162

1

4

2

1225

2

1048

60482

6

1

1

41

1

1120

3836

7715

1

7

610

4

1

8

1

3

137276

12124

3

41711

214

42917

257

263973

1

26

24221

45

1413

114

14

1123

1

25165632

11

4

73

771

316

26

52781

413

73713

7

12

172196494218

921641

151

222

132341

2

4

4

2120

6

425

328631

593916

12

3

91010

112

332

1157

69221172

5

752022742

1

9

23135

1263

17

3469

89

27125

3427

5

113

229151

124

67

42186

51

26237

2

132

15

21

3

13343096727628182

401825

2

215

11

2

2822

21

1

21

14

96

1

541424753

2

471

93371

4

8

931

101037

5917

355131

91

13239

1

2929815155

4

2517

11891

2217

114

1

5

142

1056442

27110

136

25

42979

1

113101

21

2192492

553365282

11619

11

212

21122

11

51120

238

2110

10

217

110361

11

26315

2

12

1

31

51

13331

1

11832

230

164

2711

226

1043220198

2614

871601

2104698

8110832911

8691

1

22

4236

3518

8

1119207

21311

2315

2

109

1

3188

3412241

1

8286274

2

2

1

216

1

81444

9

1

9

1825163811

11

542

15

2

5

13

6232

1523231

16089

22

114

2

14

622

25

62108

397592344

23011

26121

31312

4

535

4

115

5

72032

36

15718

16

158

1122241

12

27

347171

5

833201499378

3

3

711

82124

8

31248

366

38

7

913117

631

61211478

9

21

7191

61316

2

42067

7

190

3102261

211

1743

13

2155

111686

11

3

436

15721

215

143154683

24519

12

12

78

6447

2142

12233372

2

129151

582258412

217189

8

17

151

216

2

71

136

2

210

2

1

639

8

1

1710

3

63

181

2398

7

4

21

15

911

1152

4

110

1149

6734

116

5581

1

39

34

128

1

9

3

258

18

8

252754773

403

6

343

1

14140

41783

217

62

626

33281

229

255

14

626141

14

6

11631

4

138

5013579567

9151

1

22

420

2129

238065204

24181251

116

88

98173

3

2139

8

2

119272

1

686243

15128743

4

1

4312

6125623

165

15

6

1352

1

1252

538159

19452

3

42874

61495

2270

4511

714

858170353857504791

184648083

15

222002

1431213

96

31

13

7536

48

15

26

210

18922

56

92

54

41136

1012101

1

14

2

1

2

19

1

3

1

1

4

1

2

1

5

1107

96

111

4928855098937150

2630

41291

114

5815

1244

2216

134

41

1

3

102

1512602

1

1219

18

76611

838231

1

25

8

1216

32218

66492

8179

5

2830

8297

1

245

1236

415

21

8544979327150

335

11

1102

142453

124

1

21

245

68

4

131

39

241

4

4911

119231

2

1

26

2

13

43

1

1

3

1

21

6525

13191

41026

44

1

441

3

2

23837

4

6127

70362

1759661

218

1

5151

1

16406

249

1

27921263

112461

2

1

2

131

4222

1546242

323015734

18

12

2311

612

8

11

211

215

12112

12117

2220343

13

24

926248

24

4768

6

102357125434641

2

12

1

13611

22

1019

1

518822131

21

156

1

1

14610

1

114110

4

236531

21491

1

32

1310

30

1035

17

58

162

1

1

19

1

2347

4

335

27

13

3

376693

46623489868853187862

2748113

1

91

1

40

1

1

1

2

1

7726

184

1

2

1

1

161

24

1

1

1

1

14

20

19763513877

1229

1

13220

1

146

433391

733

2

82018

314

28

6402

793

1

211

1121

134632

2

4084

111712

163257

1

28

11

2

2733751

362651

44

7

1110

7491741

172246

61320

214712

424

63

23

179

31143

1

78

1138

31248

2

2

2719

423119920

1

3

3540

71

17

32044

111

1235

520

1

4

2

313

8

1

1

133

11

1

7

1

13

311514411

153328

12

4

1

151

14

182

110

198

1810

15

13

1114

3765

2

1251

7

331

17

23

11

2

1328

6

8351111

4

422866

12345

3434619662

5

2711

8

11450

3

48

720

2120

1

1

14

129

1736

21

117

225

18

1

169

44

11

11

501

3328462

33636

922

54

8

6

2116

635411075101

130

1

211

114

3449

29193

1

12

5643331

266319021

21179

3304131

29

814

48

22811

830331

37

40

835

114191

1402001042621923

114

71096

152

1120

8452

2

2110

262

91771

8

3

26

225

363

25661

1

3

59

343

5228

280

14632321921

26

4

5

3

1

6

2

313

22520

1113

32

1914

51616

11

16581

3

11

1115848610165757

18

3

31

115

51150

37172

621510165757

7

31144

1

251

216

9

15

11

18

355794812

11

322

2

4

7

519

6

917

7

5

7

18

271

1048782

6137

31615

21138

211

1

22

2

2

412

1

139983991112

712

1054

182211

14

12

1213

136

1520

217

4

252218

1869

2

12

1

488818121

1

1452831

54494951

2

2

652

7

247

5891

1

584211

151

23

151553

41052

771

91442438645

222311

32620

1106

11714

44

2621

449

610111

2233971

214845

115

2158

2

2

3115

112

112

2

350

22925

2387

1

67491

107172

5533358

1

1

116

1

1

3

2128108

1715

1

2

9

2214

1

33

11

27

5

153

681245991

2

6218

173

6

321

2112

8

499

239

5114

41

123

3

2

2

11

1

33

2154

11

4624

4

1

1

510117

13

1

31017
